# Network Pharmacology Study and Experimental Confirmation Revealing the Ameliorative Effects of Decursin on Chemotherapy-Induced Alopecia

**DOI:** 10.3390/ph14111150

**Published:** 2021-11-11

**Authors:** Mi Hye Kim, Sang Jun Park, Woong Mo Yang

**Affiliations:** Department of Convergence Korean Medical Science, College of Korean Medicine, Kyung Hee University, 26 Kyungheedae-ro, Dongdaemun-gu, Seoul 02447, Korea; kimmihye526@khu.ac.kr (M.H.K.); everythingisok@khu.ac.kr (S.J.P.)

**Keywords:** Decursin, chemotherapy-induced alopecia, hair follicles, network pharmacology

## Abstract

Decursin, a pyranocoumarin compound from the root of *Angelica gigas* Nakai as a main constituent, has been reported to have various biological activities, including anti-inflammatory, anticancer, and antioxidant effects. This study aimed to predict and confirm the pharmacological relevance of Decursin on chemotherapy-induced alopecia (CIA) with the underlying molecular mechanisms. Decursin-targeted genes were compared with the gene set of alopecia and investigated through functional enrichment analysis. CIA was induced in C57BL/6J mice by injection of cyclophosphamide, and 1, 10, and 100 μM of Decursin were topically treated to depilated dorsal skin. KGF^+^ expression was detected in the dorsal skin tissues. Based on the predicted results, caspase, PIK3/AKT, and MAPKs protein expressions by Decursin were analyzed in the TNF-α-induced keratinocytes. The Decursin network had 60.20% overlapped genes with the network of alopecia. Biological processes, such as cellular response to chemical stimulus, apoptosis, PI3K-AKT signaling pathway, and MAPK signaling pathway, were derived from the Decursin network. In the Decursin-treated skin, there was morphological hair growth and histological restoration of hair follicles in the CIA mice. The KGF^+^ fluorescence and protein expressions were significantly increased by Decursin treatment. In addition, caspase-3, -7, and -8 expressions, induced by TNF-α, were dose-dependently decreased along with the inhibition of PI3K, AKT, ERK, and p38 expressions in Decursin-treated keratinocytes. These findings indicated that Decursin would be a potent therapeutic option for hair loss, in response to chemotherapy.

## 1. Introduction

A hair follicle is the essential organ for the generation of hair, along with physical protection, thermal insulation, camouflage, sebaceous dispersion, sensory perception, and social interactions [1]. The hair cycle, dominated by hair follicles, consists of three stages, including anagen, the growth phase; catagen, the transition phase; and telogen, the resting phase. In healthy conditions of hair growth, the hair is constantly going to recycle the growing, regression, and resting phases, respectively [2]. Anti-cancer agents, such as doxorubicin, anthracycline, taxanes, and cyclophosphamide, are reported to induce hair follicles to shift into the resting telogen stage because of massive apoptosis in hair matrix keratinocytes. As a consequence of chemotherapy-induced abnormal hair cycle, serious hair loss occurs [3]. In chemotherapy-induced alopecia (CIA), the activity of the matrix keratinocyte is disrupted and divided into cellular subsets, leading to damaged follicular miniaturization and dystrophy of hair bulb [4]. Although CIA is not regarded as a life-threatening disease, it is a major problem to manage in clinical oncology by causing decline of self-confidence and -esteem [5].

Multiple treatments have been developed and used for CIA. Scalp cooling during chemotherapy is a primary method approved by U.S. Food and Drug Administration to reduce blood flow to hair follicles [6]. Unfortunately, its success rate for CIA varies from person to person, from 10% to 100% of the time. Scalp cooling, in addition, is reported to inhibit even tumor drug uptake [7]. Even if Minoxidil, a medication used for the treatment of androgenic alopecia, is effective in shortening the telogen stage of hair follicles after CIA, it is well known that Minoxidil would not be recommended for CIA prevention due to its clinical insufficient efficacy [8]. Prostaglandin analogue, calcitriol and miscellaneous, has been proposed and researched for prevention and treatment of CIA; nevertheless, the results have been unsatisfactory in several studies [9]. Recently, natural plant extracts containing sweet flag, have been found in the hair growth effect [10]. In addition, standardized Cinnamon bark extract was reported to prevent side effects of cancer chemotherapy, such as weight loss and alopecia [11]. Based on the previous literatures, natural product derived from traditional medicine with efficacy and low side effects, might be a new strategy for developing treatment of CIA.

Decursin has been extensively investigated to exert various biological activities including anti-inflammatory, anticancer, and antioxidant effects [12]. It is a pyranocoumarin compound, extracted from root of *Angelica gigas* Nakai as a main constituent. In our previous study, the root of *Angelica sinensis* inhibited apoptosis-derived catagen regression, inducing hair regrowth in depilated mice [13]. Additionally, Decursin and *A. gigas* root extract promoted hair growth by regulating pro- and/or anti-inflammatory cytokines in mice [14]. Recently, network pharmacology has been used to estimate the complicated association with drugs and diseases in in silico level. This analysis provides a new perspective to develop drugs by constructing candidates-target-disease network. In this study, we explored the pharmacological relevance of Decursin on alopecia with the underlying molecular mechanisms via network pharmacology analysis. Then, the potential effectiveness and therapeutic mechanism of Decursin, in the treatment of alopecia related to chemotherapy, was verified in CIA mice based on the predicted results.

## 2. Results

### 2.1. Gene Comparison Indicates the Close Relationship between Decursin and Alopecia

**Figure 1:**
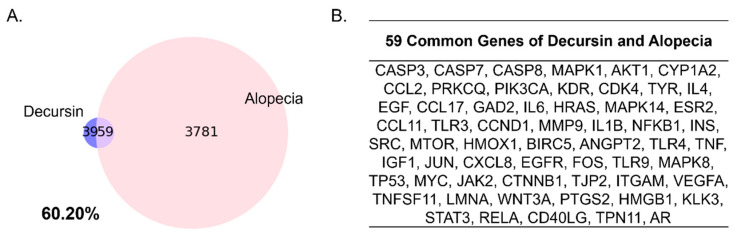
A comparison between Decursin and alopecia. (**A**) Venn diagram of intersection targets between the Decursin network and the gene sets of alopecia. (**B**) Common genes of Decursin and alopecia.

Decursin had 100 genes co-occurrences, gathered from PubChem database in literatures. Two genes were sorted out due to low confidence and relevance, resulting in construction of the Decursin network, consisting of 98 target genes. A total of 3840 genes composes the gene set of alopecia, derived from GeneCards database with the relevance score. As a result, to compare between the Decursin Network and the gene set of alopecia, based on its related genes respectively, 59 genes were overlapped between Decursin and the alopecia network. The Decursin network included 59 intersecting genes of the 98 whole genes between alopecia, leading to about 60.20% matching (Figure 1A). Screened common genes of Decursin and alopecia gene sets were CASP3, CASP7, CASP8, MAPK1, AKT1, and others (Figure 1B).

### 2.2. Functional Enrichment Analysis Shows That Underlying Mechanism of Decursin Is Predicted to Be Related to Apoptosis Pathway in CIA

**Figure 2:**
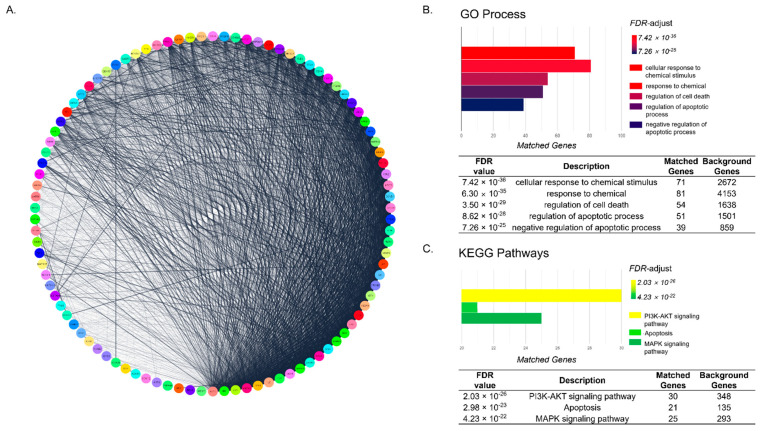
Biological processes related to targets of Decursin. (**A**) Network of Decursin with 98 nodes and 2690 edges. (**B**) Enrichment analysis extracted from GO of target genes of the Decursin network. (**C**) Enrichment analysis extracted from KEGG of target genes of the Decursin network.

The Decursin network was constructed from 98 genes. The network was composed of 98 nodes, which are target genes of Decursin, and 7595 edges, which show the connections between nodes (Figure 2A). Based on FDR-value, CIA-related pathways of the network in GO Process and KEGG Pathway, were detected. GO annotation of functional enrichment analysis revealed that Decursin-targeted genes were mainly associated with cellular response to chemical stimulus, response to chemical, regulation of cell death, regulation of apoptotic process, and negative regulation of apoptotic process (Figure 2B), with high significant FDR values. Among 98 genes of the Decursin network, 71, 81, 54, 51, and 39 genes (72.44%, 82.65%, 55.10%, 52.04%, and 39.80%, respectively) were matched with the terms of GO process; cellular response to chemical stimulus, response to chemical, regulation of cell death, regulation of apoptotic process, and negative regulation of apoptotic process, respectively. In addition, KEGG enrichment analysis showed that PI3K-AKT signaling pathway, apoptosis, and MAPK signaling pathway, were predicted as pathways of the Decursin network (Figure 2C). Moreover, 30, 21, and 25 genes of Decursin-targeted genes were overlapped with the KEGG PI3K-AKT signaling pathway, apoptosis, and MAPK signaling pathway, showing the 30.61%, 21.43%, and 25.51% matching, respectively.

### 2.3. Decursin Promotes the Growth of Hair Fiber in CIA Mice

**Figure 3:**
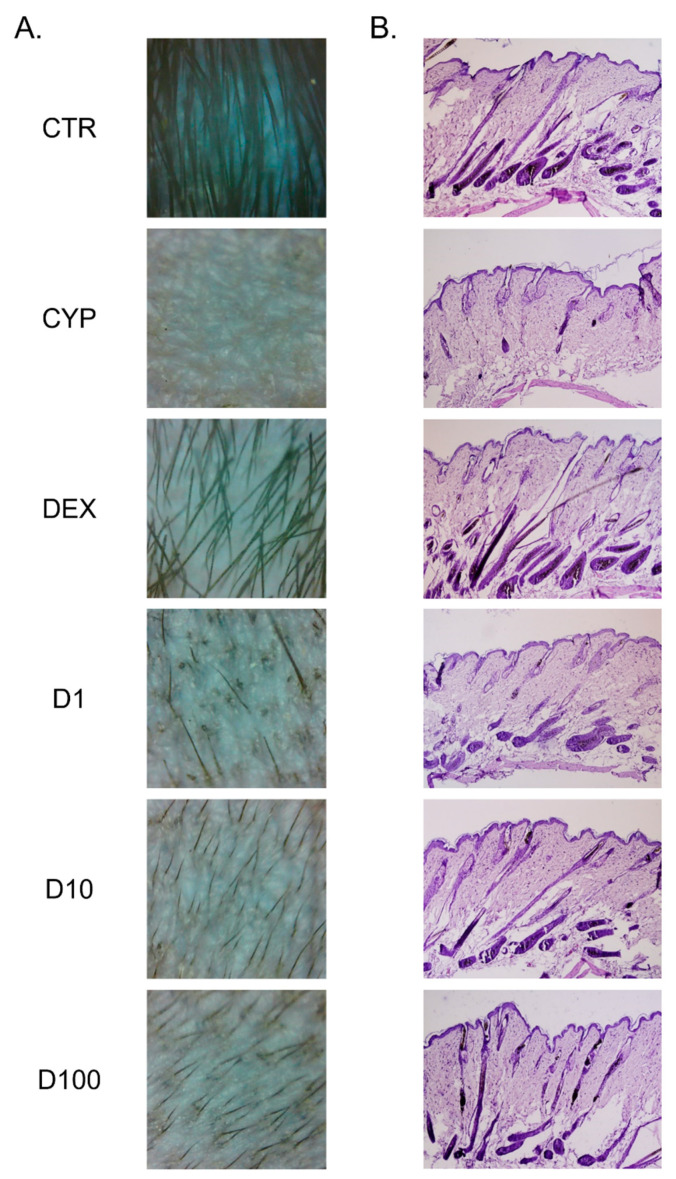
Effects of Decursin on hair growth in chemotherapy-induced alopecic mice. (**A**) Morphological changes of hair growth photographed by dermatoscope; the magnification is 10×. (**B**) Histological findings of dorsal skin tissues stained by hematoxylin and eosin; the magnification is 100×. CYP, cyclophosphamide; DEX, dexamethasone; D1, 1 μM of decursin; D10, 10 μM of decursin; D100, 100 μM of decursin.

Using a digital dermatoscope, we observed that cyclophosphamide (CYP) injection led to the dystrophic changes of hair growth in depilated skin. Topical treatment of Decursin induced newly growing hair fiber through epidermis. At all concentrations of Decursin, as well as dexamethasone (DEX) treatment, hair growth was shown in dorsum compared to mice in the CYP group (Figure 3A). Histological examination revealed morphological changes in the hair follicles after Decursin treatment. Compared to non-treated and non-injected CTR group, the hair follicles were presumed to be in the dystrophic catagen phase after one week of CYP injection. The size of bulge in the hair follicles shrunk by CYP. There appeared to be a number of hair follicles in the subcutaneous layer after Decursin treatment, indicating that hair follicles were in the anagen phase (Figure 3B). The appearance of hair bulge in the follicles was normalized in the Decursin-treated groups, compared to the CYP group. The hair shaft straightened and emerged into the surface of skin tissues.

### 2.4. Decursin Increases KGF Expression in Skin Tissues of CIA Mice and TNF-α-Stimulated Keratinocytes

**Figure 4:**
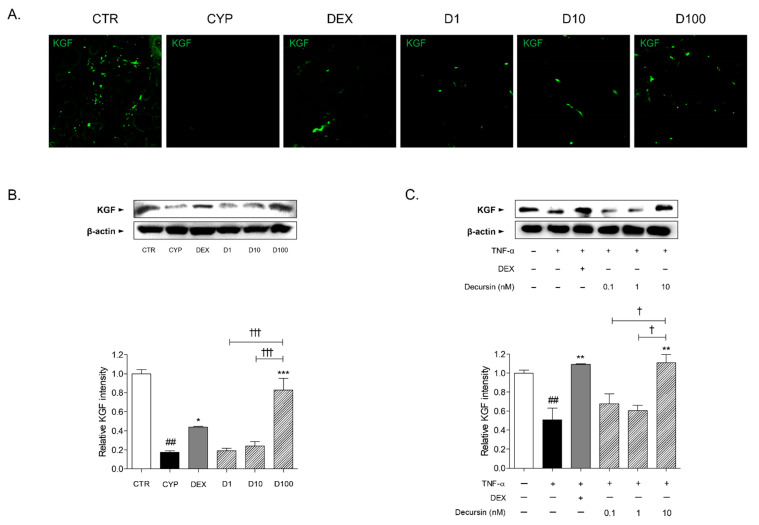
Effects of Decursin on KGF expression in chemotherapy-induced alopecic mice and keratinocytes. (**A**) Immunofluorescence images of KGF^+^ expression in hair follicles of dorsal skin tissues. Green fluorescence intensities indicate KGF; the magnification is 400×. (**B**) Protein expressions of KGF in skin tissues. Results are presented as mean ± standard error of the mean and Tukey’s multiple comparison tests were used to determine the statistical significance. ^##^ *p* < 0.01 vs. CTR group; * *p* < 0.05 and *** *p* < 0.001 vs. CYP group; ^†††^ *p* < 0.001 vs. experimental groups (D1, D10 and D100). KGF, keratinocyte growth factor; CYP, cyclophosphamide; DEX, dexamethasone; D1, 1 μM of decursin; D10, 10 μM of decursin; D100, 100 μM of decursin. (C) Protein expressions of KGF in HaCaT keratinocytes. Results are presented as mean ± standard error of the mean and Tukey’s multiple comparison tests were used to determine the statistical significance. ^##^ *p* < 0.01 vs. non-treated cells; ** *p* < 0.01 vs. TNF-α-sensitized cells; ^†^ *p* < 0.05 vs. experimental TNF-α-sensitized and Decursin-treated cells. TNF-α, tumor necrosis factor-α.

CYP injection led to reduction of keratinocyte (KGF)^+^ expression in the skin tissues, compared to skin tissues of the CTR group. Concentrations of 1, 10, and 100 μM of Decursin had an increasing effect on KGF^+^ expression of skin tissues in CIA mice (Figure 4A). In addition, the protein expression of KGF was significantly increased in the 100 μM of Decursin-treated skin tissues (Figure 4B). In the 10 nM of Decursin-treated cells, the KGF protein expression was markedly incremented, when compared with tumor necrosis factor-α (TNF-α)-sensitized HaCaT keratinocytes (Figure 4C).

### 2.5. Decursin Decreased the Expressions of Apoptosis Factors including Caspase-3, -7, and -8 in TNF-α-Stimulated Keratinocytes

**Figure 5:**
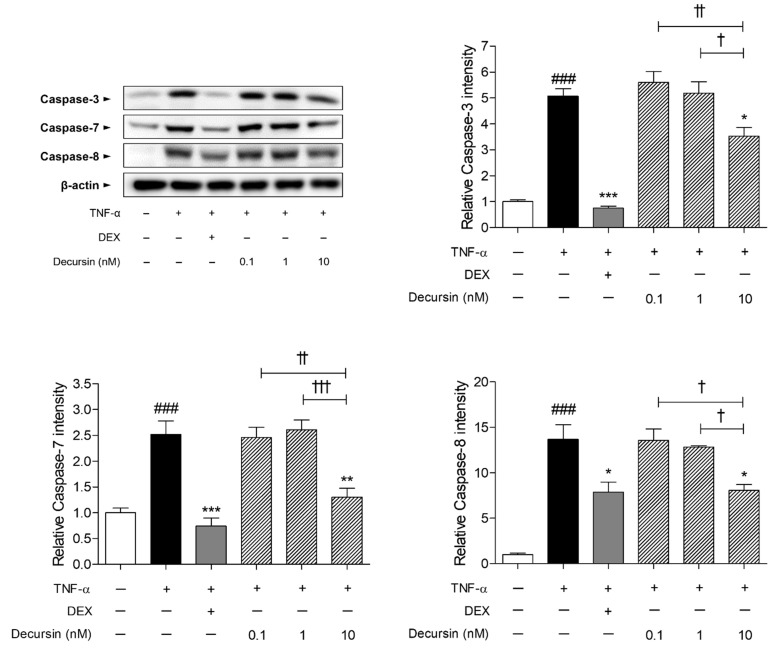
Effects of Decursin on apoptosis-related factors in keratinocytes. Results are presented as mean ± standard error of the mean and Tukey’s multiple comparison tests were used to determine the statistical significance. ^###^ *p* < 0.001 vs. non-treated cells; * *p* < 0.05, ** *p* < 0.01, and *** *p* < 0.001 vs. TNF-α-sensitized cells; ^†^ *p* < 0.05, ^††^ *p* < 0.01, and ^†††^ *p* < 0.001 vs. experimental TNF-α-sensitized and Decursin-treated cells. TNF-α, tumor necrosis factor-α.

The caspase expressions in the HaCaT keratinocytes were significantly higher than that in the non-treated cells. Increased rates of caspase-3, -7, and -8 in the TNF-α-sensitized cells were 5.07, 2.52, and 13.70, respectively, compared to the non-treated cells. Treatment with 10 nM of Decursin dose-dependently decreased the increment of protein levels of caspase-3, -7, and -8, induced by TNF-α in HaCaT cells (Figure 5). The percentages of decrease in caspase-3, -7, and -8 were 30.41%, 48.27%, and 41.07%, respectively.

### 2.6. Decursin Decreased the Expressions of PI3K/AKT Signaling and MAPKs Signaling Pathway in TNF-α-Stimulated Keratinocytes

**Figure 6:**
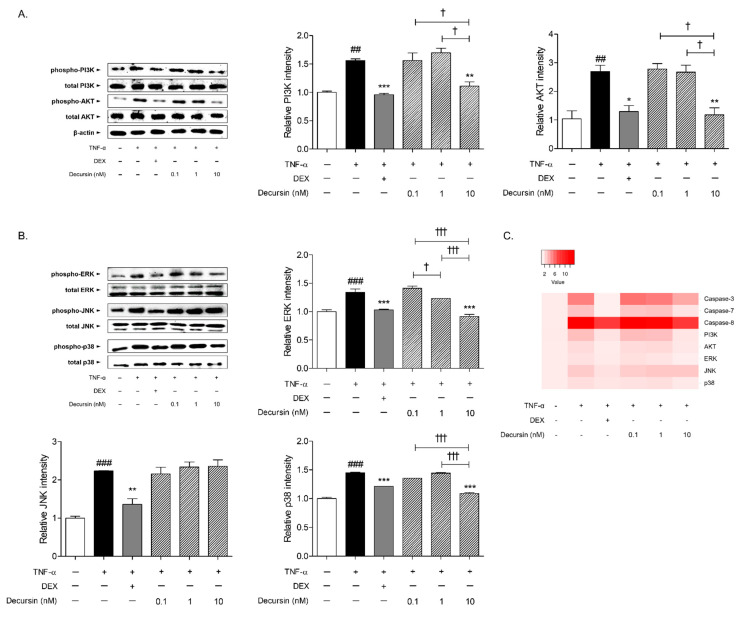
Effects of Decursin on PI3K/AKT and MAPKs pathway in keratinocytes. (**A**) Protein expressions of PI3K and AKT in TNF-α-sensitized HaCaT keratinocytes. (**B**) Protein expressions of ERK, JNK, and p38 in TNF-α-sensitized HaCaT keratinocytes. Results are presented as mean ± standard error of the mean and Tukey’s multiple comparison tests were used to determine the statistical significance. ^##^ *p* < 0.01 and ^###^ *p* < 0.001 vs. non-treated cells; * *p* < 0.05, ** *p* < 0.01, and *** *p* < 0.001 vs. TNF-α-sensitized cells; ^†^ *p* < 0.05 and ^†††^ *p* < 0.001 vs. experimental TNF-α-sensitized and Decursin-treated cells. (**C**) Heatmap color intensity of protein expressions.

About 1.56 and 2.70 times of phosphoinositide 3-kinase (PI3K) and protein kinase B (AKT) were phosphorylated in the HaCaT cells by TNF-α sensitization, compared to non-treated cells. The protein levels of phosphorylated PI3K and AKT were significantly decreased by 10 nM of Decursin treatment, by 28.79% and 56.34%, respectively (Figure 6A). The phosphorylated mitogen-activated protein kinase (MAPK)s, including extracellular signal regulated kinase (ERK), c-Jun N-terminal kinase (JNK), and p38 expressions, were increased in the presence of TNF-α in HaCaT keratinocytes. Decursin, at the 10 nM concentration, significantly diminished the protein expressions of ERK and p38 (Figure 6B). There was no significant alteration of JNK phosphorylated protein level by Decursin treatment.

## 3. Discussion

There was a significant correlation between Decursin and alopecia, showing 60.20% intersecting common rate of Decursin-targeted genes with alopecia-related genes. Through functional enrichment analysis of the Decursin network composing 98 nodes and 7595 edges, biological terms, including cellular response to chemical stimulus, response to chemical, regulation of cell death, negative regulation of apoptotic process, and regulation of apoptotic process, were derived from GO Process. From those results, the potential anti-CIA effects of Decursin were predicted.

Chemotherapy is reported to induce damaged hair follicles with matrix deterioration; as a result, dystrophic catagen is formed and hair shedding is increased [15]. Especially, premature follicle regression caused by massive apoptosis, is considered as one of the devastating and undesirable effects of chemotherapy [16]. In this study, after 9 days of depilation, hair follicles reached the early anagen VI. At that time, injection of CYP immediately led to entering hair follicles into dystrophic catagen until 16–17 days of the experiment. Hair growth was observed in the treatment of Decursin at the concentrations of 1, 10, and 100 μM from the 9th to 16th day, in CYP-induced mice. While the CYP group showed unchanged hair loss in dorsal skin of mice, newly growing hair fiber appeared through the surface of skin by Decursin treatment. In addition, Decursin histologically recovered dystrophic hair follicles, induced by CYP. Hair follicles without hair shaft in the dermis, not reaching to the dermal adipocyte layer, dystrophic catagen-like hair follicles, were induced in the CYP group. Topical application of 10 and 100 μM of Decursin exhibited the restoration of anagen-like hair follicles, which were straight to the surface of skin and emerging to the epidermis. It is well known that chemotherapy deconstructs the keratinocyte matrix and declines the growth factors, including KGF, epidermal growth factor, transforming growth factor, and parathyroid hormone-related protein, leading to the impairment of follicular proliferation and differentiation [17,18,19,20]. Especially, KGF has been shown to stimulate follicular proliferation and normalization in CIA models [17]. Based on the previous research, we further confirmed the hair growth effects of Decursin on CIA by analyzing KGF expression in hair follicles of skin tissues. Our data indicates that the Decursin-treated skin tissues showed increases of KGF^+^ immunofluorescence expression and protein level when compared with the skin tissue of the CYP group. Additionally, the protein expression of KGF was significantly increased in the TNF-α-sensitized and Decursin-treated HaCaT keratinocytes. These results suggest that Decursin could effectively restore the damaged hair cycle and induce hair regrowth in CIA, and support the prediction that Decursin has a relative connection to alopecia, particularly the chemical stimulus response.

To detect coherent pathways of Decursin on CIA, functional enrichment analysis was performed on KEGG Pathways. As a result, PI3K-AKT signaling pathway, apoptosis, and MAPK signaling pathway were predicted as pathways of the Decursin network with the high FDR-value. Taken together with the GO Process and KEGG Pathways, the pathways imply that Decursin might affect apoptosis and cell death process. At the stage of catagenic hair follicle, apoptosis-driven involution is activated by caspase production [21]. Cell death-related proteins have been reported to inhibit the proliferation of dermal papilla cells, resulting in pre-maturation of hair follicles and further hair loss [22]. For that reason, inhibition of caspase activation would be helpful to reverse the abnormal hair cycle entered into dystrophic catagen and anagen phases, and induce hair regrowth in response to chemotherapy [23]. Among the 21 matched genes from apoptosis KEGG Pathways, we specifically investigated whether Decursin manipulated the apoptotic factors such as caspase-3, -7, and -8. In the TNF-α-sensitized HaCaT keratinocytes, an in vitro condition of massive apoptosis in the hair follicles [24] and the expressions of caspase-3, -7, and -8, were significantly decreased by 10 nM of Decursin. Consistent with findings from network pharmacology, Decursin treatment decreased the protein expressions of apoptotic factors, including caspase-3, -7, and -8 in keratinocytes.

In the current study, KEGG enrichment analysis of the Decursin network revealed that the biological terms; PI3K-AKT signaling pathway, apoptosis, and MAPK signaling pathway, showed high matching rates with Decursin-targeted genes. The PI3K/AKT signaling pathway is regarded as one of the important factors to initiate and promote the primordial follicle development [25]. Chemotherapy-induced PI3K/AKT pathway activation has been demonstrated to undergo apoptosis of hair follicles [26]. Furthermore, growth factors are regulated by PI3K/AKT cascade or MAPKs pathway [27]. Due to the well-established research that CYP induced the phosphorylation of MAPKs (ERK, JNK and p38 proteins) [28], the expressions of MAPKs were analyzed in this study. Treatment of TNF-α-induced HaCaT keratinocytes with 10 nM of Decursin, significantly decreased the phosphorylated protein levels of PI3K and AKT. Also, the phospho-ERK and p38 expressions, not JNK in keratinocytes, were down-regulated in response to Decursin.

## 4. Materials and Methods

### 4.1. Network Construction and Comparison of Common Genes between Decursin and Alopecia-Targeted Genes

To construct a Decursin Network, genes related to Decursin were collected from PubChem database (PubChem; https://pubchem.ncbi.nlm.nih.gov/; accessed on 11 May 2021), which is an open database of accessible chemical information, including physical properties, biological activities, literature citations, and more. One hundred genes, which had co-occurrences in the literature with Decursin, were gathered from PubChem. The confidence score was set > 0.7 and max add 20 relevance through STRING database (STRING; http://www.string-db.org/; accessed on 15 May 2021) based on the previous literatures [29]. As a result, 98 genes were derived (Table S1). Using GeneCards database (GeneCards; http://www.genecards.org/; accessed on 15 May 2021), genes related to alopecia were collected to create an alopecia gene set (Table S2). The Decursin Network was examined to detect shared elements with the gene set of alopecia. Total 98 genes in the Decursin Network were compared with the 3840 genes in the gene set of alopecia, to assess a correlation between Decursin and alopecia. Common genes in Decursin and alopecia were used to construct a network to investigate detailed related pathways of Decursin related to CIA.

### 4.2. Functional Enrichment Analysis

Functional enrichment analysis was conducted to predict the potential molecular functions and molecular interactions between the gene set of Decursin on alopecia, by confirming GO Process and KEGG Pathways’ terms, using Cytoscape String App. The GO Process and KEGG Pathways were sorted by FDR value, which is widely used to investigate the association between genetic molecules and diseases [13].

### 4.3. Animal Experiments

All animal procedures were approved by Committee on Care and Use of Laboratory Animals of the Kyung Hee Univ. (KHUASP(SE)-13-046). Male C57BL/6J mice aged 5 weeks were purchased from RAONBIO Inc. (Yongin, Korea). All mice were maintained in plastic cages at 22 ± 1 °C temperature and 50 ± 5% humidity with 12 h light/dark cycle. After 1 week of adaptation, mice were randomly assigned to 6 groups; (1) CTR, normal control group; (2) CYP, CYP-induced alopecia group; (3) DEX, DEX-treated in CYP-induced alopecia mice as a positive control group; (4) D1, 1 μM of Decursin-treated in CYP-induced alopecia mice; (5) D10, 10 μM of Decursin-treated in CYP-induced alopecia mice; and (6) D100, 100 μM of Decursin-treated in CYP-induced alopecia mice. CYP, DEX, and Decursin were purchased from Sigma-Aldrich (St. Louis, MO, USA). To synchronize the phase of hair growth cycle, black fur of dorsal skin in all mice, except the CTR group, was shaved using shaving cream on Day 0. After 9 days, 150 mg/kg of CYP was dissolved in saline and administered intraperitoneally once to all mice, except the CTR group. Injection of CYP induces the phase of hair follicles into dystrophic catagen. From Day 9 to Day 16, 100 μL of 1, 10, and 100 μM of Decursin in saline containing 0.1% dimethyl sulfoxide, were topically treated to shaved dorsal skin, once a day. An amount of 0.1% DEX, as a positive control, was topically administered to the depilated region on Days 1, 3, 5, 7, and 9 to 16 [14]. CTR and CYP groups received normal saline during the experiment. At the start of the experiment on Day 17, all mice were sacrificed under anesthesia.

### 4.4. Skin Monitoring by Digital Dermatoscope

The digital dermatoscope used in this study was Smart Microscope Pro (Kangjin Technology, Ltd., Seoul, Korea), with 10× magnification. Dermoscopic images were acquired in the same area; the central region of the dermal skin treated samples, in all mice.

### 4.5. Histology

After sacrifice, dorsal skin tissues of all mice were collected and incubated in 10% neutralized formalin for 24 h. It was dehydrated in ethanol and xylene, and then embedded in paraffin. The skin specimens were sliced into 7 μm thick sections and stained with hematoxylin and eosin (H&E) solution.

### 4.6. Immunofluorescence

Paraffin tissue sections were hydrated with xylene and ethanol, and then blocked in 1% bovine serum albumin. The slides were incubated with primary anti-rabbit KGF antibody (Cell Signaling Technology, Danvers, MA, USA) overnight at 4 °C. Secondary horseradish peroxidase (HRP)-conjugated fluorescence antibody (Abcam, Cambridge, UK) was treated in the tissue slides at room temperature for 1 h. The stained skin tissues were observed under a fluorescence microscope (LSM 5 PASCAL; Carl Zeiss, Oberkochen, Germany).

### 4.7. Cell Treatment

Human keratinocyte HaCaT cells were grown in Dulbecco’s modified Eagle’s medium (DMEM) (Gibco; Thermo Fisher Scientific, Inc., Waltham, MA, USA), supplemented with 10% *v*/*v* fetal bovine serum (Gibco), 2 mM glutamine, 100 IU/mL penicillin, and 100 μg/mL streptomycin (Gibco), at 37 °C in an atmosphere containing 5% CO_2_ with 95% humidity. The 1 × 10^6^ cells/well HaCaT cells were seeded in 6 well plates. After stabilization, cells were co-treated with 1 μM of DEX, and 0.1, 1, and 10 nM of Decursin, in the presence of 100 ng/mL of TNF-α (PeproTech, Cranbury, NJ, USA), for 24 h.

### 4.8. Immunoblotting Analysis and Heatmap

Proteins were extracted from the collected skin tissues and HaCaT cells, using radioimmunoprecipitation assay lysis buffer (Tech & Innovation, Gangwon, Korea) containing protease inhibitor cocktail (Hoffmann-La Roche, Basel, Swiss). The lysates were quantified by Bradford method and denatured with sodium dodecylsulfate buffer at 98 °C for 5 min. The protein was electrotransferred onto a polyvinylidene fluoride membrane. The membranes were consecutively incubated with primary anti-KGF, caspase-3, -7, -8, PI3K, AKT, ERK, JNK, and p38 antibodies (Santa Cruz Biotechnology, Inc., Dallas, TX, USA), and then secondary HRP-conjugated anti-rabbit and anti-mouse antibodies (Santa Cruz Biotechnology, Inc., Dallas, TX, USA). Relative band densities were observed under ImageQuant TL (IQTL) software (GE Healthcare, Chicago, IL, USA) and quantified using a computerized densitometry system (Image J, National Institutes of Health, Bethesda, Rockville, MD, USA). Heatmap was designed to visualize mean values from experiments, based on clustering methods, in range from 1 to 13.57, which were the lowest and highest value.

### 4.9. Statistical Analysis

Significance was determined by one-way analysis of variance (ANOVA) and Tukey’s multiple comparison tests. In all analyses, *p* < 0.05 was taken to indicate statistical significance.

## 5. Conclusions

The findings of the present study focused on the prediction of efficacy and the underlying mechanism of Decursin on CIA through the network pharmacology, and the subsequent confirmation via in vivo and in vitro. The Decursin-targeted genes exerted high matched rate with alopecia, indicating the relevance of Decursin and alopecia disease. Decursin showed ameliorative effects on hair loss in CYP-treated mice. Through the enrichment analysis, cellular response to chemical stimulus, apoptosis, PI3K/AKT signaling pathway, and MAPKs signaling pathway were expected to have a correlation with the underlying mechanism of Decursin. Correspondingly, Decursin significantly inhibited the apoptotic factors, such as caspase-3, -7, and -8, and the phosphorylation of PI3K, AKT, ERK, and p38 protein expressions. In conclusion, Decursin might be one of leading candidates for CIA by inhibiting apoptosis, PI3K/AKT, and MAPKs signaling. Based on our investigation, the cross-work of prediction derived from network pharmacology analysis and confirmation in cell and rodent models, would provide effective therapeutic strategies to develop new drugs.

## Data Availability

The data presented in this study are available on request from the corresponding author. The data are not publicly available due to policy of the Kyung Hee University.

## Supplementary Materials

The following are available online at https://www.mdpi.com/article/10.3390/ph14111150/s1. Table S1: Gene targets of Decursin derived from PubChem open database, Table S2: Gene targets of alopecia derived from GeneCards open database.
