# Supplementary material for: Network Pharmacology Study and Experimental Confirmation Revealing the Ameliorative Effects of Decursin on Chemotherapy-Induced Alopecia
Source: Pharmaceuticals (Basel). 2021 Nov 11;14(11):1150. doi: 10.3390/ph14111150 (PMC8618121; doi:10.3390/ph14111150)
Supplement: Supplementary file 1 [file pharmaceuticals-14-01150-s001.zip › TableS1.pdf]

Table S1. Gene targets of Decursin derived from PubChem open database.

Compound Name	Gene Symbol	Gene Name
Decursin	CYCD1;1	cyclin-d1-1
	VEGFA	vascular endothelial growth factor a
	CASP3.2.S	caspase-3
	PARP	poly (adp-ribose) polymerase
	BCL2	bcl2 apoptosis regulator
	AR	androgen receptor
	KDR	kinase insert domain receptor
	BIRC5	baculoviral iap repeat containing 5
	CASP8	caspase 8
	IL-6	interleukin-6
	EC:2.7.11.24	mitogen-activated protein kinase
	PRKCQ	protein kinase c theta
	HMOX1	heme oxygenase 1
	EC:1.14.14.1	unspecific monooxygenase
	STAT3	signal transducer and activator of transcription 3
	MSMP	microseminoprotein, prostate associated
	EC:1.14.13.11	trans-cinnamate 4-monooxygenase
	CYP2J2	cytochrome p450 family 2 subfamily j member 2
	EC:2.4.2.30	nad(+) adp-ribosyltransferase
	EC:3.4.21.77	semenogelase
	CASP9	caspase 9
	MYC	myc proto-oncogene, bhlh transcription factor
	TNF	tumor necrosis factor
	MAPK14	mitogen-activated protein kinase 14
	CTNNB1	catenin beta 1
	WNT3A	wnt family member 3a
	AKT1	akt serine/threonine kinase 1
	MAPK8	mitogen-activated protein kinase 8
	NFATC1	nuclear factor of activated t cells 1
	CXCL8	c-x-c motif chemokine ligand 8
	CCL11	c-c motif chemokine ligand 11
	MAOA	monoamine oxidase a
	ATF4	activating transcription factor 4
	EC:4.3.1.24	phenylalanine ammonia-lyase
	EC:1.11.1.9	glutathione peroxidase
	CASP7	caspase 7
	PIK3CA	phosphatidylinositol-4,5-bisphosphate 3-kinase catalytic subunit alpha
	CDK4	cyclin dependent kinase 4
	HMGB1	high mobility group box 1
	TNFSF11	tnf superfamily member 11
	CYP1A2	cytochrome p450 family 1 subfamily a member 2
	PRPF4B	pre-mrna processing factor 4b
	FASN	fatty acid synthase
	EC:3.4.24.35	gelatinase b

	IL13	interleukin 13
	JAK2	janus kinase 2
	CYP2D15	cytochrome p450 2d15
	TYR	tyrosinase
	MCP-1	corticostatin-3
	EC:1.15.1.1	superoxide dismutase
	EC:2.4.1.21	starch synthase
	BCL2L1	bcl2 like 1
	MAPK15	mitogen-activated protein kinase 15
	TLR4	toll like receptor 4
	HIF1A	hypoxia inducible factor 1 subunit alpha
	CYP3A12	cytochrome p450 3a12
	PTGS2	prostaglandin-endoperoxide synthase 2
	MAPK4	mitogen-activated protein kinase 4
	IL4	interleukin 4
	CCK	cholecystokinin
	DCSTAMP	dendrocyte expressed seven transmembrane protein
	LATS1	large tumor suppressor kinase 1
	NKX3-1	nk3 homeobox 1
	MTOR	mechanistic target of rapamycin kinase
	ITGAM	integrin subunit alpha m
	TJP2	tight junction protein 2
	EC:1.13.12.8	watasenia-luciferin 2-monooxygenase
	LOC443319	chorionic somatomammotropin hormone
	CES2	carboxylesterase 2
	2A6	1-aminocyclopropane-1-carboxylate oxidase homolog 5
	KAT3	potassium channel kat3
	CCL17	c-c motif chemokine ligand 17
	OSM1	fumarate reductase
	CES1	carboxylesterase 1
	MITF	melanocyte inducing transcription factor
	FLK	flagellar regulator flk
	LMNA	lamin a/c
	ANGPT2	angiopoietin 2
	DDX53	dead-box helicase 53
	CDK-4	cyclin-dependent kinase 4 homolog
	CDK6	cyclin dependent kinase 6
	PIN1	peptidylprolyl cis/trans isomerase, nima-interacting 1
	NRK	nik related kinase
	ACAT1	acetyl-coa acetyltransferase 1
	EC:1.6.3.1	nad(p)h oxidase (h(2)o(2)-forming)
	TEK	tek receptor tyrosine kinase
	EIF2AK2	eukaryotic translation initiation factor 2 alpha kinase 2
	PKCDELTA	putative protein kinase c delta type homolog
	GAD2	glutamate decarboxylase 2
	TLR9	toll like receptor 9
	PTPN11	protein tyrosine phosphatase non-receptor type 11
	KIP1	kinesin-like protein kip1
	TNFRSF10B	tnf receptor superfamily member 10b
	EC:2.3.1.85	fatty-acid synthase

	TLR3	toll like receptor 3
	TPM3	tropomyosin 3
	RETN	resistin
	RPS6KB1	ribosomal protein s6 kinase b1
