# Supplementary material for: Network Pharmacology Study and Experimental Confirmation Revealing the Ameliorative Effects of Decursin on Chemotherapy-Induced Alopecia
Source: Pharmaceuticals (Basel). 2021 Nov 11;14(11):1150. doi: 10.3390/ph14111150 (PMC8618121; doi:10.3390/ph14111150)
Supplement: Supplementary file 1 [file pharmaceuticals-14-01150-s001.zip › TableS2.pdf]

Table S2. Gene targets of alopecia derived from GeneCards open database.

Gene Symbol	Description	Relevance Score
HR	HR Lysine Demethylase And Nuclear Receptor Corepressor	83.67
FOXN1	Forkhead Box N1	80.41
DCAF17	DDB1 And CUL4 Associated Factor 17	61.38
VDR	Vitamin D Receptor	39.28
AIRE	Autoimmune Regulator	38.26
GJA1	Gap Junction Protein Alpha 1	34.88
LSS	Lanosterol Synthase	34.25
MBTPS2	Membrane Bound Transcription Factor Peptidase, Site 2	33.94
RBM28	RNA Binding Motif Protein 28	30.71
IL2RA	Interleukin 2 Receptor Subunit Alpha	30.7
KRT83	Keratin 83	30.67
DSG4	Desmoglein 4	30.56
IL10	Interleukin 10	30.3
GJB2	Gap Junction Protein Beta 2	30.12
AHSG	Alpha 2-HS Glycoprotein	30.1
LIPH	Lipase H	29.78
CTLA4	Cytotoxic T-Lymphocyte Associated Protein 4	29.49
HLA-DRB1	Major Histocompatibility Complex, Class II, DR Beta 1	29.33
PTPN22	Protein Tyrosine Phosphatase Non-Receptor Type 22	29.01
LPAR6	Lysophosphatidic Acid Receptor 6	28.34
FOXP3	Forkhead Box P3	27.89
HLA-B	Major Histocompatibility Complex, Class I, B	27.57
TP63	Tumor Protein P63	27.32
ALX4	ALX Homeobox 4	26.58
KRT16	Keratin 16	26.48
HLA-DQB1	Major Histocompatibility Complex, Class II, DQ Beta 1	26.43
RIN2	Ras And Rab Interactor 2	26
AA1	Alopecia Areata 1	25.43
HOXC13	Homeobox C13	25.35
EDNRA	Endothelin Receptor Type A	25.27
DSP	Desmoplakin	25.14
PADI3	Peptidyl Arginine Deiminase 3	25
KRT14	Keratin 14	24.73
GJB4	Gap Junction Protein Beta 4	24.35
AA2	Alopecia Areata 2	24.33
KRT86	Keratin 86	24.1
GJB3	Gap Junction Protein Beta 3	24

HLA-DQA1	Major Histocompatibility Complex, Class II, DQ Alpha 1	23.92
FLG	Filaggrin	23.48
TRPS1	Transcriptional Repressor GATA Binding 1	23.32
TNFRSF1B	TNF Receptor Superfamily Member 1B	23.24
KRT74	Keratin 74	22.88
SASH1	SAM And SH3 Domain Containing 1	21.7
COL7A1	Collagen Type VII Alpha 1 Chain	21.64
EDAR	Ectodysplasin A Receptor	20.98
JAK3	Janus Kinase 3	20.66
KRT85	Keratin 85	20.55
LMNA	Lamin A/C	20.5
AR	Androgen Receptor	20.32
AGA2	Alopecia, Androgenetic, 2	20.16
AGA3	Alopecia, Androgenetic, 3	20.16
IFNG	Interferon Gamma	20
AFA1	Alopecia, Androgenetic	19.69
KRT81	Keratin 81	19.61
HTRA1	HtrA Serine Peptidase 1	19.37
CASR	Calcium Sensing Receptor	19.33
ANTXR1	ANTXR Cell Adhesion Molecule 1	19.19
CLDN1	Claudin 1	19.17
TGM1	Transglutaminase 1	18.86
SLC30A2	Solute Carrier Family 30 Member 2	18.75
TNF	Tumor Necrosis Factor	18.41
BTD	Biotinidase	18.33
GJB6	Gap Junction Protein Beta 6	17.84
TRPV3	Transient Receptor Potential Cation Channel Subfamily V Member 3	17.71
WNT10A	Wnt Family Member 10A	17.55
IKBKG	Inhibitor Of Nuclear Factor Kappa B Kinase Regulatory Subunit Gamma	17.4
SLC39A4	Solute Carrier Family 39 Member 4	17.34
IL2	Interleukin 2	17.27
HLA-A	Major Histocompatibility Complex, Class I, A	16.75
IL4	Interleukin 4	16.75
ULBP3	UL16 Binding Protein 3	16.45
EDARADD	EDAR Associated Death Domain	15.98
APMR1	Alopecia-Mental Retardation Syndrome	15.87
NFKB2	Nuclear Factor Kappa B Subunit 2	15.81
COL17A1	Collagen Type XVII Alpha 1 Chain	15.56
PLEC	Plectin	15.54
TCHH	Trichohyalin	15.43

KLRK1	Killer Cell Lectin Like Receptor K1	15.38
IL1RN	Interleukin 1 Receptor Antagonist	15.36
IL6	Interleukin 6	15.21
SNRPE	Small Nuclear Ribonucleoprotein Polypeptide E	15.2
STX17	Syntaxin 17	15.18
KRT17	Keratin 17	15.18
SRD5A1	Steroid 5 Alpha-Reductase 1	15.18
PHEX	Phosphate Regulating Endopeptidase Homolog X-Linked	14.87
IL1B	Interleukin 1 Beta	14.79
ICOSLG	Inducible T Cell Costimulator Ligand	14.76
KRT25	Keratin 25	14.74
TTC7A	Tetratricopeptide Repeat Domain 7A	14.56
CD28	CD28 Molecule	14.48
DKC1	Dyskerin Pseudouridine Synthase 1	14.48
ZMPSTE24	Zinc Metallopeptidase STE24	14.48
CD4	CD4 Molecule	14.43
KRT6A	Keratin 6A	14.41
EBP	EBP Cholestenol Delta-Isomerase	14.31
IL1A	Interleukin 1 Alpha	14.29
JUP	Junction Plakoglobin	14.16
CXCL10	C-X-C Motif Chemokine Ligand 10	14.13
HLA-C	Major Histocompatibility Complex, Class I, C	14.08
UQCRFS1	Ubiquinol-Cytochrome C Reductase, Rieske Iron-Sulfur Polypeptide 1	14.07
IL17A	Interleukin 17A	14.03
ADA	Adenosine Deaminase	13.87
ALOXE3	Arachidonate Lipoxygenase 3	13.87
EPS8L3	EPS8 Like 3	13.87
CERS3	Ceramide Synthase 3	13.87
PTPRC	Protein Tyrosine Phosphatase Receptor Type C	13.77
SRD5A2	Steroid 5 Alpha-Reductase 2	13.77
IL18	Interleukin 18	13.71
ITGB4	Integrin Subunit Beta 4	13.71
HRAS	HRas Proto-Oncogene, GTPase	13.68
IL7R	Interleukin 7 Receptor	13.68
KRT6B	Keratin 6B	13.68
SLITRK1	SLIT And NTRK Like Family Member 1	13.68
CD8A	CD8a Molecule	13.63
FASLG	Fas Ligand	13.61
SHBG	Sex Hormone Binding Globulin	13.56
IL13	Interleukin 13	13.55

CXCL9	C-X-C Motif Chemokine Ligand 9	13.52
ICAM1	Intercellular Adhesion Molecule 1	13.47
HLCS	Holocarboxylase Synthetase	13.41
CYP21A2	Cytochrome P450 Family 21 Subfamily A Member 2	13.31
LAMA3	Laminin Subunit Alpha 3	13.27
ALOX12B	Arachidonate 12-Lipoxygenase, 12R Type	13.22
PNPLA1	Patatin Like Phospholipase Domain Containing 1	13.22
CNGA3	Cyclic Nucleotide Gated Channel Subunit Alpha 3	13.18
KRT2	Keratin 2	13.18
XPA	XPA, DNA Damage Recognition And Repair Factor	13.04
NECTIN1	Nectin Cell Adhesion Molecule 1	13.04
APMR2	Alopecia With Mental Retardation Syndrome 2	13.03
NOTCH4	Notch Receptor 4	12.86
CDSN	Corneodesmosin	12.84
ATR	ATR Serine/Threonine Kinase	12.77
KRT75	Keratin 75	12.74
MICA	MHC Class I Polypeptide-Related Sequence A	12.63
TPO	Thyroid Peroxidase	12.6
FAS	Fas Cell Surface Death Receptor	12.57
STAT1	Signal Transducer And Activator Of Transcription 1	12.57
LORICRIN	Loricrin Cornified Envelope Precursor Protein	12.57
STAT4	Signal Transducer And Activator Of Transcription 4	12.54
ABCA12	ATP Binding Cassette Subfamily A Member 12	12.54
NIPAL4	NIPA Like Domain Containing 4	12.54
MIF	Macrophage Migration Inhibitory Factor	12.51
ITGB6	Integrin Subunit Beta 6	12.48
NFKB1	Nuclear Factor Kappa B Subunit 1	12.35
KRAS	KRAS Proto-Oncogene, GTPase	12.35
LIG4	DNA Ligase 4	12.35
ERCC4	ERCC Excision Repair 4, Endonuclease Catalytic Subunit	12.35
RMRP	RNA Component Of Mitochondrial RNA Processing Endoribonuclease	12.35
CXCR3	C-X-C Motif Chemokine Receptor 3	12.2
APMR3	Alopecia-Mental Retardation Syndrome 3	12.19
LAMB3	Laminin Subunit Beta 3	12.08
IL1R1	Interleukin 1 Receptor Type 1	12.06
CLEC16A	C-Type Lectin Domain Containing 16A	12.04
CRH	Corticotropin Releasing Hormone	12.02
TLR2	Toll Like Receptor 2	11.93
METTL8	Methyltransferase Like 8	11.92
PRDX5	Peroxiredoxin 5	11.91

TGM3	Transglutaminase 3	11.88
IKZF4	IKAROS Family Zinc Finger 4	11.84
NSDHL	NAD(P) Dependent Steroid Dehydrogenase-Like	11.82
RIPK4	Receptor Interacting Serine/Threonine Kinase 4	11.75
MC2R	Melanocortin 2 Receptor	11.65
ZNF341	Zinc Finger Protein 341	11.64
USB1	U6 SnRNA Biogenesis Phosphodiesterase 1	11.64
RAG2	Recombination Activating 2	11.63
FGFR1	Fibroblast Growth Factor Receptor 1	11.63
PERP	P53 Apoptosis Effector Related To PMP22	11.63
RAG1	Recombination Activating 1	11.58
DCLRE1C	DNA Cross-Link Repair 1C	11.58
RECQL4	RecQ Like Helicase 4	11.52
CYP19A1	Cytochrome P450 Family 19 Subfamily A Member 1	11.49
LAMC2	Laminin Subunit Gamma 2	11.48
GZMB	Granzyme B	11.44
ERCC1	ERCC Excision Repair 1, Endonuclease Non-Catalytic Subunit	11.43
CYP4F22	Cytochrome P450 Family 4 Subfamily F Member 22	11.43
SULT2B1	Sulfotransferase Family 2B Member 1	11.24
H2AC18	H2A Clustered Histone 18	11.24
TLR4	Toll Like Receptor 4	11.11
KRT71	Keratin 71	11.07
KLHL24	Kelch Like Family Member 24	11.03
IGF1	Insulin Like Growth Factor 1	10.91
STS	Steroid Sulfatase	10.91
IL2RG	Interleukin 2 Receptor Subunit Gamma	10.85
TINF2	TERF1 Interacting Nuclear Factor 2	10.85
PKP1	Plakophilin 1	10.83
ALB	Albumin	10.81
PRL	Prolactin	10.81
BCS1L	BCS1 Homolog, Ubiquinol-Cytochrome C Reductase Complex Chaperone	10.8
KIT	KIT Proto-Oncogene, Receptor Tyrosine Kinase	10.66
IL23R	Interleukin 23 Receptor	10.66
SPP1	Secreted Phosphoprotein 1	10.64
EDA2R	Ectodysplasin A2 Receptor	10.6
MMP1	Matrix Metallopeptidase 1	10.23
ECM1	Extracellular Matrix Protein 1	10.23
CHD7	Chromodomain Helicase DNA Binding Protein 7	10.19
EDA	Ectodysplasin A	10.14
RAET1L	Retinoic Acid Early Transcript 1L	10.13

NECTIN4	Nectin Cell Adhesion Molecule 4	10.07
TGFB2	Transforming Growth Factor Beta 2	10.01
WNT5A	Wnt Family Member 5A	10
PEX7	Peroxisomal Biogenesis Factor 7	10
C4A	Complement C4A (Rodgers Blood Group)	9.96
CCR6	C-C Motif Chemokine Receptor 6	9.92
SDR9C7	Short Chain Dehydrogenase/Reductase Family 9C Member 7	9.92
KDM5C	Lysine Demethylase 5C	9.88
PORCN	Porcupine O-Acyltransferase	9.88
APCDD1	APC Down-Regulated 1	9.86
NOD2	Nucleotide Binding Oligomerization Domain Containing 2	9.74
BANF1	BAF Nuclear Assembly Factor 1	9.74
TNFRSF1A	TNF Receptor Superfamily Member 1A	9.69
XIAP	X-Linked Inhibitor Of Apoptosis	9.66
RHOA	Ras Homolog Family Member A	9.65
ERCC2	ERCC Excision Repair 2, TFIIH Core Complex Helicase Subunit	9.52
GTF2H5	General Transcription Factor IIH Subunit 5	9.52
LINC00536	Long Intergenic Non-Protein Coding RNA 536	9.47
ADAM10	ADAM Metallopeptidase Domain 10	9.46
CYP27B1	Cytochrome P450 Family 27 Subfamily B Member 1	9.43
KRT15	Keratin 15	9.39
SMARCA2	SWI/SNF Related, Matrix Associated, Actin Dependent Regulator Of Chromatin, Subfamily A, Member 2	9.38
JAK1	Janus Kinase 1	9.3
CFLAR	CASP8 And FADD Like Apoptosis Regulator	9.19
LCOR	Ligand Dependent Nuclear Receptor Corepressor	9.11
LBR	Lamin B Receptor	9.08
HLA-DRA	Major Histocompatibility Complex, Class II, DR Alpha	9.06
ABHD5	Abhydrolase Domain Containing 5, Lysophosphatidic Acid Acyltransferase	9.06
FAM111B	Family With Sequence Similarity 111 Member B	9.06
GNAS	GNAS Complex Locus	8.89
ITGAM	Integrin Subunit Alpha M	8.89
ST14	ST14 Transmembrane Serine Protease Matriptase	8.89
GJB5	Gap Junction Protein Beta 5	8.89
ECTD6	Ectodermal Dysplasia 6, Hair/Nail Type	8.89
CDH15	Cadherin 15	8.66
WDR11	WD Repeat Domain 11	8.66
INS	Insulin	8.62
ERCC3	ERCC Excision Repair 3, TFIIH Core Complex Helicase Subunit	8.45
RNF113A	Ring Finger Protein 113A	8.45
MPLKIP	M-Phase Specific PLK1 Interacting Protein	8.45

SCP2	Sterol Carrier Protein 2	8.43
IFNA1	Interferon Alpha 1	8.23
IL5	Interleukin 5	8.19
CSN1S1	Casein Alpha S1	8.19
CD40LG	CD40 Ligand	8.14
LIPN	Lipase Family Member N	8.14
AUTS2	Activator Of Transcription And Developmental Regulator AUTS2	8.11
NOTCH1	Notch Receptor 1	8
TERT	Telomerase Reverse Transcriptase	8
NRAS	NRAS Proto-Oncogene, GTPase	8
GNA11	G Protein Subunit Alpha 11	8
BMP2	Bone Morphogenetic Protein 2	8
ERCC5	ERCC Excision Repair 5, Endonuclease	8
UROD	Uroporphyrinogen Decarboxylase	8
MCCC2	Methylcrotonoyl-CoA Carboxylase 2	8
HFE	Homeostatic Iron Regulator	8
RPL21	Ribosomal Protein L21	8
KDSR	3-Ketodihydrosphingosine Reductase	8
ARHGAP31	Rho GTPase Activating Protein 31	8
SOX18	SRY-Box Transcription Factor 18	8
DOCK6	Dedicator Of Cytokinesis 6	8
TERC	Telomerase RNA Component	8
FGFR3	Fibroblast Growth Factor Receptor 3	7.95
PIK3CA	Phosphatidylinositol-4,5-Bisphosphate 3-Kinase Catalytic Subunit Alpha	7.95
TNFAIP3	TNF Alpha Induced Protein 3	7.95
FCGR3B	Fc Fragment Of IgG Receptor IIIb	7.95
IL17F	Interleukin 17F	7.95
DCAF8	DDB1 And CUL4 Associated Factor 8	7.95
MIR155	MicroRNA 155	7.95
MUHH2	Hypotrichosis, Hereditary, Marie Unna Type, 2	7.95
TARDBP	TAR DNA Binding Protein	7.9
PPARG	Peroxisome Proliferator Activated Receptor Gamma	7.86
CTNNB1	Catenin Beta 1	7.7
GRK5	G Protein-Coupled Receptor Kinase 5	7.7
SQLE	Squalene Epoxidase	7.7
TIMM8A	Translocase Of Inner Mitochondrial Membrane 8A	7.7
GCAT	Glycine C-Acetyltransferase	7.7
PPIC	Peptidylprolyl Isomerase C	7.7
LDHD	Lactate Dehydrogenase D	7.7
PTCRA	Pre T Cell Antigen Receptor Alpha	7.7

IFRD2	Interferon Related Developmental Regulator 2	7.7
CASS4	Cas Scaffold Protein Family Member 4	7.7
TXLNG	Taxilin Gamma	7.7
LPP	LIM Domain Containing Preferred Translocation Partner In Lipoma	7.69
IVL	Involucrin	7.67
PARN	Poly(A)-Specific Ribonuclease	7.54
CCL2	C-C Motif Chemokine Ligand 2	7.49
SOD1	Superoxide Dismutase 1	7.46
CD40	CD40 Molecule	7.46
TMPO	Thymopoietin	7.44
KDM4C	Lysine Demethylase 4C	7.44
HELLS	Helicase, Lymphoid Specific	7.4
IL12A	Interleukin 12A	7.39
CXCL8	C-X-C Motif Chemokine Ligand 8	7.2
COL3A1	Collagen Type III Alpha 1 Chain	7.19
GTF2E2	General Transcription Factor IIE Subunit 2	7.19
TARS1	Threonyl-TRNA Synthetase 1	7.19
TP53	Tumor Protein P53	7.17
CRP	C-Reactive Protein	7.17
KLK3	Kallikrein Related Peptidase 3	6.93
PNPLA6	Patatin Like Phospholipase Domain Containing 6	6.92
NUDT15	Nudix Hydrolase 15	6.9
IFNGR1	Interferon Gamma Receptor 1	6.89
PRKDC	Protein Kinase, DNA-Activated, Catalytic Subunit	6.89
BLM	BLM RecQ Like Helicase	6.89
CCR1	C-C Motif Chemokine Receptor 1	6.89
BCL10	BCL10 Immune Signaling Adaptor	6.89
ERCC6	ERCC Excision Repair 6, Chromatin Remodeling Factor	6.89
PSTPIP1	Proline-Serine-Threonine Phosphatase Interacting Protein 1	6.89
HAMP	Hepcidin Antimicrobial Peptide	6.89
IL12RB1	Interleukin 12 Receptor Subunit Beta 1	6.89
MEFV	MEFV Innate Immuity Regulator, Pyrin	6.89
PEX5	Peroxisomal Biogenesis Factor 5	6.89
C4B	Complement C4B (Chido Blood Group)	6.89
MIR17	MicroRNA 17	6.89
ARCI7	Ichthyosis, Congenital, Autosomal Recessive 7	6.89
ECTD5	Ectodermal Dysplasia 5, Hair/Nail Type	6.89
TGFB1	Transforming Growth Factor Beta 1	6.83
IFNA2	Interferon Alpha 2	6.82
CDKN2A	Cyclin Dependent Kinase Inhibitor 2A	6.8
WRN	WRN RecQ Like Helicase	6.78

CARD11	Caspase Recruitment Domain Family Member 11	6.77
SUOX	Sulfite Oxidase	6.76
CD80	CD80 Molecule	6.76
PRKACA	Protein Kinase CAMP-Activated Catalytic Subunit Alpha	6.73
IRAK1	Interleukin 1 Receptor Associated Kinase 1	6.73
FLI1	Fli-1 Proto-Oncogene, ETS Transcription Factor	6.73
ROR2	Receptor Tyrosine Kinase Like Orphan Receptor 2	6.73
FTL	Ferritin Light Chain	6.73
DDB2	Damage Specific DNA Binding Protein 2	6.73
COL18A1	Collagen Type XVIII Alpha 1 Chain	6.73
DLL4	Delta Like Canonical Notch Ligand 4	6.73
NHP2	NHP2 Ribonucleoprotein	6.73
UROS	Uroporphyrinogen III Synthase	6.73
NXN	Nucleoredoxin	6.73
NOP10	NOP10 Ribonucleoprotein	6.73
RTEL1	Regulator Of Telomere Elongation Helicase 1	6.73
BTNL2	Butyrophilin Like 2	6.73
DMXL2	Dmx Like 2	6.73
SMAD4	SMAD Family Member 4	6.64
NR1H2	Nuclear Receptor Subfamily 1 Group H Member 2	6.64
TNFSF4	TNF Superfamily Member 4	6.61
STAT3	Signal Transducer And Activator Of Transcription 3	6.59
HSPD1	Heat Shock Protein Family D (Hsp60) Member 1	6.59
ADIPOQ	Adiponectin, C1Q And Collagen Domain Containing	6.59
SERPINA3	Serpin Family A Member 3	6.59
TNFRSF8	TNF Receptor Superfamily Member 8	6.59
DACT1	Dishevelled Binding Antagonist Of Beta Catenin 1	6.59
RSPO4	R-Spondin 4	6.59
TNFSF13B	TNF Superfamily Member 13b	6.57
TRAC	T Cell Receptor Alpha Constant	6.55
DKK1	Dickkopf WNT Signaling Pathway Inhibitor 1	6.49
ATM	ATM Serine/Threonine Kinase	6.47
CSF3	Colony Stimulating Factor 3	6.47
CCL5	C-C Motif Chemokine Ligand 5	6.45
ERAP1	Endoplasmic Reticulum Aminopeptidase 1	6.43
ATXN2	Ataxin 2	6.43
HNRNPA3	Heterogeneous Nuclear Ribonucleoprotein A3	6.4
ODC1	Ornithine Decarboxylase 1	6.38
LEP	Leptin	6.37
CCL11	C-C Motif Chemokine Ligand 11	6.37
CCL17	C-C Motif Chemokine Ligand 17	6.37

ENPP1	Ectonucleotide Pyrophosphatase/Phosphodiesterase 1	6.29
ATP12A	ATPase H+/K+ Transporting Non-Gastric Alpha2 Subunit	6.29
ATP4A	ATPase H+/K+ Transporting Subunit Alpha	6.29
TIA1	TIA1 Cytotoxic Granule Associated RNA Binding Protein	6.28
LINC00708	Long Intergenic Non-Protein Coding RNA 708	6.06
SELE	Selectin E	6.02
LTA	Lymphotoxin Alpha	6.02
PTGDS	Prostaglandin D2 Synthase	6.01
TWIST2	Twist Family BHLH Transcription Factor 2	6.01
DST	Dystonin	6
PTEN	Phosphatase And Tensin Homolog	5.96
BMPR1A	Bone Morphogenetic Protein Receptor Type 1A	5.96
CD19	CD19 Molecule	5.96
PC	Pyruvate Carboxylase	5.96
PNPO	Pyridoxamine 5'-Phosphate Oxidase	5.96
MUTYH	MutY DNA Glycosylase	5.96
SLC5A6	Solute Carrier Family 5 Member 6	5.96
GJC1	Gap Junction Protein Gamma 1	5.96
IL7	Interleukin 7	5.96
GXYLT2	Glucoside Xylosyltransferase 2	5.96
HECTD4	HECT Domain E3 Ubiquitin Protein Ligase 4	5.96
KRT5	Keratin 5	5.94
FBLN5	Fibulin 5	5.94
CD86	CD86 Molecule	5.94
TLR9	Toll Like Receptor 9	5.93
PIK3R1	Phosphoinositide-3-Kinase Regulatory Subunit 1	5.89
POLG	DNA Polymerase Gamma, Catalytic Subunit	5.84
PDCD1	Programmed Cell Death 1	5.81
VEGFA	Vascular Endothelial Growth Factor A	5.81
CSF2	Colony Stimulating Factor 2	5.81
EVC2	EvC Ciliary Complex Subunit 2	5.81
EVC	EvC Ciliary Complex Subunit 1	5.81
LEMD3	LEM Domain Containing 3	5.76
IL21	Interleukin 21	5.75
KITLG	KIT Ligand	5.71
SLC30A6	Solute Carrier Family 30 Member 6	5.67
TYK2	Tyrosine Kinase 2	5.62
TLR3	Toll Like Receptor 3	5.62
BLK	BLK Proto-Oncogene, Src Family Tyrosine Kinase	5.62
STK11	Serine/Threonine Kinase 11	5.62
IRF5	Interferon Regulatory Factor 5	5.62

GJB1	Gap Junction Protein Beta 1	5.62
LRP1	LDL Receptor Related Protein 1	5.62
FCGR2B	Fc Fragment Of IgG Receptor IIb	5.62
EDN1	Endothelin 1	5.62
EDN3	Endothelin 3	5.62
ENO1	Enolase 1	5.62
PTH	Parathyroid Hormone	5.62
MYH7	Myosin Heavy Chain 7	5.62
GNAI3	G Protein Subunit Alpha I3	5.62
SLC40A1	Solute Carrier Family 40 Member 1	5.62
FCGR2A	Fc Fragment Of IgG Receptor IIa	5.62
UBE2L3	Ubiquitin Conjugating Enzyme E2 L3	5.62
CR2	Complement C3d Receptor 2	5.62
TFR2	Transferrin Receptor 2	5.62
XRCC5	X-Ray Repair Cross Complementing 5	5.62
CSF1	Colony Stimulating Factor 1	5.62
HAL	Histidine Ammonia-Lyase	5.62
DOCK8	Dedicator Of Cytokinesis 8	5.62
SULT1A1	Sulfotransferase Family 1A Member 1	5.62
TREX1	Three Prime Repair Exonuclease 1	5.62
C9orf72	C9orf72-SMCR8 Complex Subunit	5.62
DNASE1	Deoxyribonuclease 1	5.62
CDH17	Cadherin 17	5.62
TNIP1	TNFAIP3 Interacting Protein 1	5.62
AFAP1	Actin Filament Associated Protein 1	5.62
BMS1	BMS1 Ribosome Biogenesis Factor	5.62
HNRNPH1	Heterogeneous Nuclear Ribonucleoprotein H1	5.62
LGALS4	Galectin 4	5.62
PXK	PX Domain Containing Serine/Threonine Kinase Like	5.62
BANK1	B Cell Scaffold Protein With Ankyrin Repeats 1	5.62
HNRNPH2	Heterogeneous Nuclear Ribonucleoprotein H2	5.62
TUBA3C	Tubulin Alpha 3c	5.62
UBAC2	UBA Domain Containing 2	5.62
KLRC4	Killer Cell Lectin Like Receptor C4	5.62
KRT33B	Keratin 33B	5.62
HJV	Hemojuvelin BMP Co-Receptor	5.62
TMEM184C	Transmembrane Protein 184C	5.62
KRT28	Keratin 28	5.62
PRMT9	Protein Arginine Methyltransferase 9	5.62
CARMIL2	Capping Protein Regulator And Myosin 1 Linker 2	5.62
PEAK3	PEAK Family Member 3	5.62

MIR342	MicroRNA 342	5.62
IL12A-AS1	IL12A Antisense RNA 1	5.62
CANDF1	Candidiasis, Familial 1	5.62
SOD2	Superoxide Dismutase 2	5.62
CD34	CD34 Molecule	5.56
IL16	Interleukin 16	5.49
TLR1	Toll Like Receptor 1	5.48
EFNA3	Ephrin A3	5.48
MX1	MX Dynamin Like GTPase 1	5.48
CCL4	C-C Motif Chemokine Ligand 4	5.48
CCL3	C-C Motif Chemokine Ligand 3	5.48
CD200	CD200 Molecule	5.48
ELN	Elastin	5.45
KRT1	Keratin 1	5.45
IGHE	Immunoglobulin Heavy Constant Epsilon	5.45
ABCD1	ATP Binding Cassette Subfamily D Member 1	5.41
POMC	Proopiomelanocortin	5.4
IRF4	Interferon Regulatory Factor 4	5.37
ALMS1	ALMS1 Centrosome And Basal Body Associated Protein	5.35
TXNRD2	Thioredoxin Reductase 2	5.32
FGF5	Fibroblast Growth Factor 5	5.3
NR3C1	Nuclear Receptor Subfamily 3 Group C Member 1	5.29
DMPK	DM1 Protein Kinase	5.27
STUB1	STIP1 Homology And U-Box Containing Protein 1	5.27
OFD1	OFD1 Centriole And Centriolar Satellite Protein	5.27
IL2RB	Interleukin 2 Receptor Subunit Beta	5.26
THRA	Thyroid Hormone Receptor Alpha	5.26
WAS	WASP Actin Nucleation Promoting Factor	5.26
CD3D	CD3d Molecule	5.26
SLC11A1	Solute Carrier Family 11 Member 1	5.26
ACADS	Acyl-CoA Dehydrogenase Short Chain	5.26
CD79A	CD79a Molecule	5.26
HNRNPA1	Heterogeneous Nuclear Ribonucleoprotein A1	5.26
PCCB	Propionyl-CoA Carboxylase Subunit Beta	5.26
FUS	FUS RNA Binding Protein	5.26
CD2	CD2 Molecule	5.26
TP53BP1	Tumor Protein P53 Binding Protein 1	5.26
SLC39A13	Solute Carrier Family 39 Member 13	5.26
SLC39A14	Solute Carrier Family 39 Member 14	5.26
SLC39A8	Solute Carrier Family 39 Member 8	5.26
PURA	Purine Rich Element Binding Protein A	5.26

XRCC4	X-Ray Repair Cross Complementing 4	5.26
NCL	Nucleolin	5.26
UBQLN2	Ubiquilin 2	5.26
MATR3	Matrin 3	5.26
ERCC8	ERCC Excision Repair 8, CSA Ubiquitin Ligase Complex Subunit	5.26
UNC119	Unc-119 Lipid Binding Chaperone	5.26
RANGAP1	Ran GTPase Activating Protein 1	5.26
ADARB2	Adenosine Deaminase RNA Specific B2 (Inactive)	5.26
MADD	MAP Kinase Activating Death Domain	5.26
MOCS1	Molybdenum Cofactor Synthesis 1	5.26
IL15	Interleukin 15	5.26
CD7	CD7 Molecule	5.26
SLC39A1	Solute Carrier Family 39 Member 1	5.26
SUPT4H1	SPT4 Homolog, DSIF Elongation Factor Subunit	5.26
PDPR	Pyruvate Dehydrogenase Phosphatase Regulatory Subunit	5.26
NAT9	N-Acetyltransferase 9 (Putative)	5.26
SIGLEC5	Sialic Acid Binding Ig Like Lectin 5	5.26
PRND	Prion Like Protein Doppel	5.26
NUP54	Nucleoporin 54	5.26
TMX2	Thioredoxin Related Transmembrane Protein 2	5.26
WDR41	WD Repeat Domain 41	5.26
CASC3	CASC3 Exon Junction Complex Subunit	5.26
CRISP1	Cysteine Rich Secretory Protein 1	5.26
MMD	Monocyte To Macrophage Differentiation Associated	5.26
H2AX	H2A.X Variant Histone	5.26
KRT82	Keratin 82	5.26
KRT84	Keratin 84	5.26
SMCR8	SMCR8-C9orf72 Complex Subunit	5.26
KRTAP3-1	Keratin Associated Protein 3-1	5.26
VSIG8	V-Set And Immunoglobulin Domain Containing 8	5.26
SNHG28	Small Nucleolar RNA Host Gene 28	5.26
MTHFR	Methylenetetrahydrofolate Reductase	5.25
GAPDH	Glyceraldehyde-3-Phosphate Dehydrogenase	5.25
BGN	Biglycan	5.25
ESR1	Estrogen Receptor 1	5.18
ACTB	Actin Beta	5.18
PAX1	Paired Box 1	5.15
CHUK	Component Of Inhibitor Of Nuclear Factor Kappa B Kinase Complex	5.12
HLA-DQA2	Major Histocompatibility Complex, Class II, DQ Alpha 2	5.11
BTK	Bruton Tyrosine Kinase	5.09

ACVR1	Activin A Receptor Type 1	5.09
NPM1	Nucleophosmin 1	5.09
FZD2	Frizzled Class Receptor 2	5.09
DVL1	Dishevelled Segment Polarity Protein 1	5.09
RBPJ	Recombination Signal Binding Protein For Immunoglobulin Kappa J Region	5.09
CSTB	Cystatin B	5.09
DVL3	Dishevelled Segment Polarity Protein 3	5.09
XPC	XPC Complex Subunit, DNA Damage Recognition And Repair Factor	5.09
AP1B1	Adaptor Related Protein Complex 1 Subunit Beta 1	5.09
SLC29A3	Solute Carrier Family 29 Member 3	5.09
UBR1	Ubiquitin Protein Ligase E3 Component N-Recognin 1	5.09
MGP	Matrix Gla Protein	5.09
EXOSC2	Exosome Component 2	5.09
WRAP53	WD Repeat Containing Antisense To TP53	5.09
AEBP1	AE Binding Protein 1	5.09
EOGT	EGF Domain Specific O-Linked N-Acetylglucosamine Transferase	5.09
CTC1	CST Telomere Replication Complex Component 1	5.09
RNU4ATAC	RNA, U4atac Small Nuclear (U12-Dependent Splicing)	5.09
CALCA	Calcitonin Related Polypeptide Alpha	5.08
APOH	Apolipoprotein H	5.06
IL31RA	Interleukin 31 Receptor A	5.06
KRT10	Keratin 10	5.06
SLC30A4	Solute Carrier Family 30 Member 4	5.06
SLC39A2	Solute Carrier Family 39 Member 2	5.06
CXCL12	C-X-C Motif Chemokine Ligand 12	4.94
PTCH2	Patched 2	4.94
FCGR1A	Fc Fragment Of IgG Receptor Ia	4.94
DSG1	Desmoglein 1	4.93
CDH3	Cadherin 3	4.88
ZAP70	Zeta Chain Of T Cell Receptor Associated Protein Kinase 70	4.87
SIRT1	Sirtuin 1	4.87
AK2	Adenylate Kinase 2	4.87
CD3G	CD3g Molecule	4.87
CP	Ceruloplasmin	4.87
SH2D1A	SH2 Domain Containing 1A	4.87
PNP	Purine Nucleoside Phosphorylase	4.87
TLR5	Toll Like Receptor 5	4.87
WNT3A	Wnt Family Member 3A	4.87
GNPAT	Glyceronephosphate O-Acyltransferase	4.87
FGF23	Fibroblast Growth Factor 23	4.87

LAMA1	Laminin Subunit Alpha 1	4.87
HSPG2	Heparan Sulfate Proteoglycan 2	4.87
CORO1A	Coronin 1A	4.87
PGM3	Phosphoglucomutase 3	4.87
KRT4	Keratin 4	4.87
ICMT	Isoprenylcysteine Carboxyl Methyltransferase	4.87
NHEJ1	Non-Homologous End Joining Factor 1	4.87
SLC30A5	Solute Carrier Family 30 Member 5	4.87
SLC39A7	Solute Carrier Family 39 Member 7	4.87
BGLAP	Bone Gamma-Carboxyglutamate Protein	4.87
SLC39A10	Solute Carrier Family 39 Member 10	4.87
SLC39A5	Solute Carrier Family 39 Member 5	4.87
SLC39A6	Solute Carrier Family 39 Member 6	4.87
SLC30A1	Solute Carrier Family 30 Member 1	4.87
SLC30A7	Solute Carrier Family 30 Member 7	4.87
SLC39A12	Solute Carrier Family 39 Member 12	4.87
MIA2	MIA SH3 Domain ER Export Factor 2	4.87
KRT35	Keratin 35	4.87
H3-2	H3.2 Histone (Putative)	4.87
MIR500A	MicroRNA 500a	4.87
FEN1	Flap Structure-Specific Endonuclease 1	4.78
IRF8	Interferon Regulatory Factor 8	4.78
CRYAA	Crystallin Alpha A	4.78
TRAF3IP2	TRAF3 Interacting Protein 2	4.78
IRF6	Interferon Regulatory Factor 6	4.78
FGF7	Fibroblast Growth Factor 7	4.76
DPP4	Dipeptidyl Peptidase 4	4.63
PAH	Phenylalanine Hydroxylase	4.63
CASP14	Caspase 14	4.63
HDAC4	Histone Deacetylase 4	4.62
WNT3	Wnt Family Member 3	4.58
ALDH1L1	Aldehyde Dehydrogenase 1 Family Member L1	4.58
SHH	Sonic Hedgehog Signaling Molecule	4.57
IHH	Indian Hedgehog Signaling Molecule	4.57
CCR4	C-C Motif Chemokine Receptor 4	4.57
TNFRSF10A	TNF Receptor Superfamily Member 10a	4.57
RBP1	Retinol Binding Protein 1	4.54
MFN2	Mitofusin 2	4.5
EGF	Epidermal Growth Factor	4.5
AKT1	AKT Serine/Threonine Kinase 1	4.44
CSF1R	Colony Stimulating Factor 1 Receptor	4.44

IKBKB	Inhibitor Of Nuclear Factor Kappa B Kinase Subunit Beta	4.44
MYC	MYC Proto-Oncogene, BHLH Transcription Factor	4.44
G6PD	Glucose-6-Phosphate Dehydrogenase	4.44
DNMT1	DNA Methyltransferase 1	4.44
MYB	MYB Proto-Oncogene, Transcription Factor	4.44
MPO	Myeloperoxidase	4.44
ROCK1	Rho Associated Coiled-Coil Containing Protein Kinase 1	4.44
LPL	Lipoprotein Lipase	4.44
BUB1B	BUB1 Mitotic Checkpoint Serine/Threonine Kinase B	4.44
STAT5B	Signal Transducer And Activator Of Transcription 5B	4.44
RAC1	Rac Family Small GTPase 1	4.44
CYCS	Cytochrome C, Somatic	4.44
IGF2	Insulin Like Growth Factor 2	4.44
SIRT2	Sirtuin 2	4.44
SIRT3	Sirtuin 3	4.44
CCR3	C-C Motif Chemokine Receptor 3	4.44
HADH	Hydroxyacyl-CoA Dehydrogenase	4.44
TNFRSF11B	TNF Receptor Superfamily Member 11b	4.44
TRAF6	TNF Receptor Associated Factor 6	4.44
TLR8	Toll Like Receptor 8	4.44
LMNB1	Lamin B1	4.44
GAD2	Glutamate Decarboxylase 2	4.44
GGT1	Gamma-Glutamyltransferase 1	4.44
TLR7	Toll Like Receptor 7	4.44
PRPS1	Phosphoribosyl Pyrophosphate Synthetase 1	4.44
ACAN	Aggrecan	4.44
RFC1	Replication Factor C Subunit 1	4.44
EMD	Emerin	4.44
IL10RA	Interleukin 10 Receptor Subunit Alpha	4.44
SIRT5	Sirtuin 5	4.44
SIRT6	Sirtuin 6	4.44
RAN	RAN, Member RAS Oncogene Family	4.44
PPARA	Peroxisome Proliferator Activated Receptor Alpha	4.44
XRCC6	X-Ray Repair Cross Complementing 6	4.44
VCAM1	Vascular Cell Adhesion Molecule 1	4.44
CBX5	Chromobox 5	4.44
GMPS	Guanine Monophosphate Synthase	4.44
FCGR3A	Fc Fragment Of IgG Receptor IIIa	4.44
ANK3	Ankyrin 3	4.44
EIF2B4	Eukaryotic Translation Initiation Factor 2B Subunit Delta	4.44
RUVBL2	RuvB Like AAA ATPase 2	4.44

WIPF1	WAS/WASL Interacting Protein Family Member 1	4.44
LIPC	Lipase C, Hepatic Type	4.44
HIBCH	3-Hydroxyisobutyryl-CoA Hydrolase	4.44
SPG7	SPG7 Matrix AAA Peptidase Subunit, Paraplegin	4.44
RBBP4	RB Binding Protein 4, Chromatin Remodeling Factor	4.44
EFNA5	Ephrin A5	4.44
SUV39H1	Suppressor Of Variegation 3-9 Homolog 1	4.44
LMNB2	Lamin B2	4.44
DMP1	Dentin Matrix Acidic Phosphoprotein 1	4.44
SELL	Selectin L	4.44
SIRT7	Sirtuin 7	4.44
SLC34A3	Solute Carrier Family 34 Member 3	4.44
SYNE1	Spectrin Repeat Containing Nuclear Envelope Protein 1	4.44
UBA7	Ubiquitin Like Modifier Activating Enzyme 7	4.44
MAGT1	Magnesium Transporter 1	4.44
ADAMTS17	ADAM Metallopeptidase With Thrombospondin Type 1 Motif 17	4.44
SIRT4	Sirtuin 4	4.44
NUP153	Nucleoporin 153	4.44
VNN2	Vanin 2	4.44
RRAGD	Ras Related GTP Binding D	4.44
LEMD2	LEM Domain Nuclear Envelope Protein 2	4.44
TTC37	Tetratricopeptide Repeat Domain 37	4.44
SYNE2	Spectrin Repeat Containing Nuclear Envelope Protein 2	4.44
NAT10	N-Acetyltransferase 10	4.44
RCE1	Ras Converting CAAX Endopeptidase 1	4.44
DLGAP3	DLG Associated Protein 3	4.44
GPS1	G Protein Pathway Suppressor 1	4.44
PGGT1B	Protein Geranylgeranyltransferase Type I Subunit Beta	4.44
SUN1	Sad1 And UNC84 Domain Containing 1	4.44
SUN2	Sad1 And UNC84 Domain Containing 2	4.44
LYSMD4	LysM Domain Containing 4	4.44
SPATA22	Spermatogenesis Associated 22	4.44
TMEM41A	Transmembrane Protein 41A	4.44
SYNE3	Spectrin Repeat Containing Nuclear Envelope Family Member 3	4.44
PRSS54	Serine Protease 54	4.44
SYNE4	Spectrin Repeat Containing Nuclear Envelope Family Member 4	4.44
WFDC12	WAP Four-Disulfide Core Domain 12	4.44
FAM71E1	Family With Sequence Similarity 71 Member E1	4.44
SPATA46	Spermatogenesis Associated 46	4.44
LEXM	Lymphocyte Expansion Molecule	4.44
MIR9-1	MicroRNA 9-1	4.44

IGHV4-38-2	Immunoglobulin Heavy Variable 4-38-2	4.44
H6PD	Hexose-6-Phosphate Dehydrogenase/Glucose 1-Dehydrogenase	4.38
IFNB1	Interferon Beta 1	4.38
CST6	Cystatin E/M	4.38
SRY	Sex Determining Region Y	4.34
IL4R	Interleukin 4 Receptor	4.31
TRAF1	TNF Receptor Associated Factor 1	4.31
FCRL3	Fc Receptor Like 3	4.31
BRAF	B-Raf Proto-Oncogene, Serine/Threonine Kinase	4.3
ICOS	Inducible T Cell Costimulator	4.29
CIITA	Class II Major Histocompatibility Complex Transactivator	4.25
MICB	MHC Class I Polypeptide-Related Sequence B	4.25
ZNF577	Zinc Finger Protein 577	4.25
RMI2	RecQ Mediated Genome Instability 2	4.25
MIR223	MicroRNA 223	4.25
RAF1	Raf-1 Proto-Oncogene, Serine/Threonine Kinase	4.24
CASP3	Caspase 3	4.24
TF	Transferrin	4.17
FGFR2	Fibroblast Growth Factor Receptor 2	4.16
RAD51	RAD51 Recombinase	4.16
PTCH1	Patched 1	4.16
DSC2	Desmocollin 2	4.16
HMGA2	High Mobility Group AT-Hook 2	4.16
LOC107988021	IKBKG Recombination Region	4.16
LOC107988022	IKBKG Downstream Recombination Region	4.16
SLC5A11	Solute Carrier Family 5 Member 11	4.15
CXCL1	C-X-C Motif Chemokine Ligand 1	4.11
TOP1	DNA Topoisomerase I	4.07
CYP17A1	Cytochrome P450 Family 17 Subfamily A Member 1	4.04
LAMC1	Laminin Subunit Gamma 1	4.04
PTPN11	Protein Tyrosine Phosphatase Non-Receptor Type 11	3.98
CXCR4	C-X-C Motif Chemokine Receptor 4	3.98
GAD1	Glutamate Decarboxylase 1	3.98
CALR	Calreticulin	3.98
DNMT3A	DNA Methyltransferase 3 Alpha	3.98
RAC2	Rac Family Small GTPase 2	3.98
CAT	Catalase	3.98
DRD2	Dopamine Receptor D2	3.98
DNMT3B	DNA Methyltransferase 3 Beta	3.98
SYK	Spleen Associated Tyrosine Kinase	3.98
GATA3	GATA Binding Protein 3	3.98

ETS1	ETS Proto-Oncogene 1, Transcription Factor	3.98
ACE	Angiotensin I Converting Enzyme	3.98
RIPK1	Receptor Interacting Serine/Threonine Kinase 1	3.98
TTR	Transthyretin	3.98
AHR	Aryl Hydrocarbon Receptor	3.98
REN	Renin	3.98
POLH	DNA Polymerase Eta	3.98
FZD6	Frizzled Class Receptor 6	3.98
NLRP3	NLR Family Pyrin Domain Containing 3	3.98
SLC1A1	Solute Carrier Family 1 Member 1	3.98
CCR5	C-C Motif Chemokine Receptor 5	3.98
CLU	Clusterin	3.98
DRD4	Dopamine Receptor D4	3.98
GATA1	GATA Binding Protein 1	3.98
ENG	Endoglin	3.98
CCR7	C-C Motif Chemokine Receptor 7	3.98
APPL1	Adaptor Protein, Phosphotyrosine Interacting With PH Domain And Leucine Zipper 1	3.98
MECP2	Methyl-CpG Binding Protein 2	3.98
CYP1A2	Cytochrome P450 Family 1 Subfamily A Member 2	3.98
SAT1	Spermidine/Spermine N1-Acetyltransferase 1	3.98
NCAM1	Neural Cell Adhesion Molecule 1	3.98
SMAD9	SMAD Family Member 9	3.98
HSD11B2	Hydroxysteroid 11-Beta Dehydrogenase 2	3.98
SLC2A4	Solute Carrier Family 2 Member 4	3.98
TXK	TXK Tyrosine Kinase	3.98
STAT5A	Signal Transducer And Activator Of Transcription 5A	3.98
TRPM4	Transient Receptor Potential Cation Channel Subfamily M Member 4	3.98
PON1	Paraoxonase 1	3.98
ARID1B	AT-Rich Interaction Domain 1B	3.98
CLEC7A	C-Type Lectin Domain Containing 7A	3.98
MMP12	Matrix Metallopeptidase 12	3.98
DRD3	Dopamine Receptor D3	3.98
IL17RA	Interleukin 17 Receptor A	3.98
PRTN3	Proteinase 3	3.98
PNPLA2	Patatin Like Phospholipase Domain Containing 2	3.98
POFUT1	Protein O-Fucosyltransferase 1	3.98
TRAF2	TNF Receptor Associated Factor 2	3.98
LAMA4	Laminin Subunit Alpha 4	3.98
TLR6	Toll Like Receptor 6	3.98
THBD	Thrombomodulin	3.98

CARD14	Caspase Recruitment Domain Family Member 14	3.98
HOXA13	Homeobox A13	3.98
DLGAP1	DLG Associated Protein 1	3.98
PRSS8	Serine Protease 8	3.98
IL12RB2	Interleukin 12 Receptor Subunit Beta 2	3.98
IFNGR2	Interferon Gamma Receptor 2	3.98
LCT	Lactase	3.98
RBCK1	RANBP2-Type And C3HC4-Type Zinc Finger Containing 1	3.98
MUS81	MUS81 Structure-Specific Endonuclease Subunit	3.98
PIGQ	Phosphatidylinositol Glycan Anchor Biosynthesis Class Q	3.98
CCK	Cholecystokinin	3.98
CD5	CD5 Molecule	3.98
RNF31	Ring Finger Protein 31	3.98
SLC38A2	Solute Carrier Family 38 Member 2	3.98
RECQL	RecQ Like Helicase	3.98
ACSM1	Acyl-CoA Synthetase Medium Chain Family Member 1	3.98
CWC27	CWC27 Spliceosome Associated Cyclophilin	3.98
IL17RC	Interleukin 17 Receptor C	3.98
PQBP1	Polyglutamine Binding Protein 1	3.98
POGLUT1	Protein O-Glucosyltransferase 1	3.98
KCTD1	Potassium Channel Tetramerization Domain Containing 1	3.98
TLR10	Toll Like Receptor 10	3.98
IL33	Interleukin 33	3.98
SHARPIN	SHANK Associated RH Domain Interactor	3.98
PPP1R13L	Protein Phosphatase 1 Regulatory Subunit 13 Like	3.98
SLC15A4	Solute Carrier Family 15 Member 4	3.98
JAZF1	JAZF Zinc Finger 1	3.98
SNRNP70	Small Nuclear Ribonucleoprotein U1 Subunit 70	3.98
KIAA0319L	KIAA0319 Like	3.98
MRAP	Melanocortin 2 Receptor Accessory Protein	3.98
SLITRK5	SLIT And NTRK Like Family Member 5	3.98
PHLDA2	Pleckstrin Homology Like Domain Family A Member 2	3.98
KIR3DL2	Killer Cell Immunoglobulin Like Receptor, Three Ig Domains And Long Cytoplasmic Tail 2	3.98
HOXB8	Homeobox B8	3.98
SLITRK2	SLIT And NTRK Like Family Member 2	3.98
SLITRK3	SLIT And NTRK Like Family Member 3	3.98
TBL1X	Transducin Beta Like 1 X-Linked	3.98
MEPE	Matrix Extracellular Phosphoglycoprotein	3.98
CLNK	Cytokine Dependent Hematopoietic Cell Linker	3.98
MTCP1	Mature T Cell Proliferation 1	3.98

WDR20	WD Repeat Domain 20	3.98
ALX3	ALX Homeobox 3	3.98
SCGB2A1	Secretoglobin Family 2A Member 1	3.98
SPZ1	Spermatogenic Leucine Zipper 1	3.98
FOXQ1	Forkhead Box Q1	3.98
PRR4	Proline Rich 4	3.98
CATIP	Ciliogenesis Associated TTC17 Interacting Protein	3.98
TMEM238	Transmembrane Protein 238	3.98
APELA	Apelin Receptor Early Endogenous Ligand	3.98
MIR24-1	MicroRNA 24-1	3.98
CANDN1	Candidiasis, Nail 1 (With ICAM1 Deficiency)	3.98
CELIAC2	Celiac Disease 2	3.98
SLEB3	Systemic Lupus Erythematosus Susceptibility 3	3.98
LPRS	Leprosy, Susceptibility To 1	3.98
LPRS6	Leprosy, Susceptiblity To, 6	3.98
ATOD1	Dermatitis, Atopic	3.98
ATOD3	Dermatitis, Atopic, 3	3.98
ATOD5	Dermatitis, Atopic, 5	3.98
ATOD6	Dermatitis, Atopic, 6	3.98
ATOD7	Dermatitis, Atopic, Susceptibility To, 7	3.98
ATOD8	Dermatitis, Atopic, 8	3.98
ATOD9	Dermatitis, Atopic, 9	3.98
CELIAC10	Celiac Disease, Susceptibility To, 10	3.98
CELIAC11	Celiac Disease, Susceptibility To, 11	3.98
CELIAC12	Celiac Disease, Susceptibility To, 12	3.98
CELIAC13	Celiac Disease, Susceptibility To, 13	3.98
CELIAC5	Celiac Disease, Susceptibility To, 5	3.98
CELIAC6	Celiac Disease, Susceptibility To, 6	3.98
CELIAC7	Celiac Disease, Susceptibility To, 7	3.98
CELIAC8	Celiac Disease, Susceptibility To, 8	3.98
CELIAC9	Celiac Disease, Susceptibility To, 9	3.98
EDSS2	Ectodermal Dysplasia-Syndactyly Syndrome 2	3.98
DEL18P	Chromosome 18p Deletion Syndrome	3.98
SLEB12	Systemic Lupus Erythematosus, Susceptibility To, 12	3.98
SLEB13	Systemic Lupus Erythematosus, Susceptibility To, 13	3.98
SLEB14	Systemic Lupus Erythematosus, Susceptiblity To, 14	3.98
SLEB15	Systemic Lupus Erthematosus, Susceptibility To, 15	3.98
SLEB4	Systemic Lupus Erythematosus, Susceptibility To, 4	3.98
SLEB5	Systemic Lupus Erythematosus, Susceptibility To, 5	3.98
SLEB7	Systemic Lupus Erythematosus, Susceptibility To, 7	3.98
SLEB8	Systemic Lupus Erythematosus, Susceptibility To, 8	3.98

SS3	Sarcoidosis, Susceptibility To, 3	3.98
HYPT9	Hypotrichosis 9	3.98
LOC107988024	IKBKGP1 Recombination Region	3.98
LOC107988025	IKBKGP1 Upstream Recombination Region	3.98
DUSP22	Dual Specificity Phosphatase 22	3.95
NF1	Neurofibromin 1	3.85
PSIP1	PC4 And SFRS1 Interacting Protein 1	3.8
NOTCH3	Notch Receptor 3	3.73
IL36RN	Interleukin 36 Receptor Antagonist	3.72
HLA-DRB3	Major Histocompatibility Complex, Class II, DR Beta 3	3.72
DDC	Dopa Decarboxylase	3.71
TH	Tyrosine Hydroxylase	3.71
TPH1	Tryptophan Hydroxylase 1	3.71
APEX1	Apurinic/Apyrimidinic Endodeoxyribonuclease 1	3.68
XRCC1	X-Ray Repair Cross Complementing 1	3.68
GLI1	GLI Family Zinc Finger 1	3.63
TRIM21	Tripartite Motif Containing 21	3.63
KDM5A	Lysine Demethylase 5A	3.63
INCENP	Inner Centromere Protein	3.63
KRT24	Keratin 24	3.63
IGKC	Immunoglobulin Kappa Constant	3.63
PLCG1	Phospholipase C Gamma 1	3.61
CYP27A1	Cytochrome P450 Family 27 Subfamily A Member 1	3.61
C1R	Complement C1r	3.61
C1S	Complement C1s	3.61
PTHLH	Parathyroid Hormone Like Hormone	3.61
CTPS1	CTP Synthase 1	3.61
PEX14	Peroxisomal Biogenesis Factor 14	3.61
SKIV2L	Ski2 Like RNA Helicase	3.61
ERAP2	Endoplasmic Reticulum Aminopeptidase 2	3.61
RPTN	Repetin	3.61
HRNR	Hornerin	3.61
TSPEAR	Thrombospondin Type Laminin G Domain And EAR Repeats	3.61
PSORS1C1	Psoriasis Susceptibility 1 Candidate 1	3.61
IL3	Interleukin 3	3.59
HDAC1	Histone Deacetylase 1	3.57
GH1	Growth Hormone 1	3.57
ITGB2	Integrin Subunit Beta 2	3.57
NR0B1	Nuclear Receptor Subfamily 0 Group B Member 1	3.57
CCL27	C-C Motif Chemokine Ligand 27	3.57
NOS3	Nitric Oxide Synthase 3	3.49

MUC1	Mucin 1, Cell Surface Associated	3.49
TAP1	Transporter 1, ATP Binding Cassette Subfamily B Member	3.49
C5	Complement C5	3.49
NKX2-1	NK2 Homeobox 1	3.49
KRT7	Keratin 7	3.49
GNLY	Granulysin	3.49
MIR30D	MicroRNA 30d	3.49
PTGDR2	Prostaglandin D2 Receptor 2	3.47
JAK2	Janus Kinase 2	3.44
AKT3	AKT Serine/Threonine Kinase 3	3.44
SLC2A1	Solute Carrier Family 2 Member 1	3.44
LCK	LCK Proto-Oncogene, Src Family Tyrosine Kinase	3.44
ALK	ALK Receptor Tyrosine Kinase	3.44
COMT	Catechol-O-Methyltransferase	3.44
PRKAR1A	Protein Kinase CAMP-Dependent Type I Regulatory Subunit Alpha	3.44
LYN	LYN Proto-Oncogene, Src Family Tyrosine Kinase	3.44
CD247	CD247 Molecule	3.44
WNT7A	Wnt Family Member 7A	3.44
EPAS1	Endothelial PAS Domain Protein 1	3.44
B2M	Beta-2-Microglobulin	3.44
F2	Coagulation Factor II, Thrombin	3.44
APOA1	Apolipoprotein A1	3.44
LRP5	LDL Receptor Related Protein 5	3.44
FANCA	FA Complementation Group A	3.44
ABCA1	ATP Binding Cassette Subfamily A Member 1	3.44
CYP11A1	Cytochrome P450 Family 11 Subfamily A Member 1	3.44
MVK	Mevalonate Kinase	3.44
CD36	CD36 Molecule	3.44
EGLN1	Egl-9 Family Hypoxia Inducible Factor 1	3.44
TNNT2	Troponin T2, Cardiac Type	3.44
HSP90AA1	Heat Shock Protein 90 Alpha Family Class A Member 1	3.44
TFRC	Transferrin Receptor	3.44
C3	Complement C3	3.44
MEF2A	Myocyte Enhancer Factor 2A	3.44
MBL2	Mannose Binding Lectin 2	3.44
MALT1	MALT1 Paracaspase	3.44
HADHB	Hydroxyacyl-CoA Dehydrogenase Trifunctional Multienzyme Complex Subunit Beta	3.44
NCF2	Neutrophil Cytosolic Factor 2	3.44
OTC	Ornithine Carbamoyltransferase	3.44
LIPE	Lipase E, Hormone Sensitive Type	3.44

FST	Follistatin	3.44
CEL	Carboxyl Ester Lipase	3.44
HSD17B4	Hydroxysteroid 17-Beta Dehydrogenase 4	3.44
F5	Coagulation Factor V	3.44
FECH	Ferrochelatase	3.44
AICDA	Activation Induced Cytidine Deaminase	3.44
BCL6	BCL6 Transcription Repressor	3.44
ADH5	Alcohol Dehydrogenase 5 (Class III), Chi Polypeptide	3.44
CCNH	Cyclin H	3.44
RORC	RAR Related Orphan Receptor C	3.44
CD27	CD27 Molecule	3.44
ELOVL4	ELOVL Fatty Acid Elongase 4	3.44
SMARCB1	SWI/SNF Related, Matrix Associated, Actin Dependent Regulator Of Chromatin, Subfamily B, Member 1	3.44
TBX21	T-Box Transcription Factor 21	3.44
OXTR	Oxytocin Receptor	3.44
SELP	Selectin P	3.44
TIMP1	TIMP Metallopeptidase Inhibitor 1	3.44
ADAR	Adenosine Deaminase RNA Specific	3.44
FOXC2	Forkhead Box C2	3.44
HP	Haptoglobin	3.44
CNDP1	Carnosine Dipeptidase 1	3.44
HMGB1	High Mobility Group Box 1	3.44
CDC45	Cell Division Cycle 45	3.44
PEX1	Peroxisomal Biogenesis Factor 1	3.44
SNRPB	Small Nuclear Ribonucleoprotein Polypeptides B And B1	3.44
NLRP1	NLR Family Pyrin Domain Containing 1	3.44
ITGAX	Integrin Subunit Alpha X	3.44
DLX5	Distal-Less Homeobox 5	3.44
AZGP1	Alpha-2-Glycoprotein 1, Zinc-Binding	3.44
LRPAP1	LDL Receptor Related Protein Associated Protein 1	3.44
BMP6	Bone Morphogenetic Protein 6	3.44
EXO1	Exonuclease 1	3.44
DHRS3	Dehydrogenase/Reductase 3	3.44
PEX2	Peroxisomal Biogenesis Factor 2	3.44
OCA2	OCA2 Melanosomal Transmembrane Protein	3.44
IL9	Interleukin 9	3.44
LCN2	Lipocalin 2	3.44
RANBP1	RAN Binding Protein 1	3.44
KCNA4	Potassium Voltage-Gated Channel Subfamily A Member 4	3.44
SUFU	SUFU Negative Regulator Of Hedgehog Signaling	3.44

PEX6	Peroxisomal Biogenesis Factor 6	3.44
HIRA	Histone Cell Cycle Regulator	3.44
TOP3A	DNA Topoisomerase III Alpha	3.44
TBX1	T-Box Transcription Factor 1	3.44
CD1D	CD1d Molecule	3.44
TG	Thyroglobulin	3.44
ARFGEF2	ADP Ribosylation Factor Guanine Nucleotide Exchange Factor 2	3.44
CLTCL1	Clathrin Heavy Chain Like 1	3.44
HCCS	Holocytochrome C Synthase	3.44
DNA2	DNA Replication Helicase/Nuclease 2	3.44
ELOVL1	ELOVL Fatty Acid Elongase 1	3.44
SPINK5	Serine Peptidase Inhibitor Kazal Type 5	3.44
PLS3	Plastin 3	3.44
SLC24A5	Solute Carrier Family 24 Member 5	3.44
KLK5	Kallikrein Related Peptidase 5	3.44
RAD52	RAD52 Homolog, DNA Repair Protein	3.44
CD1A	CD1a Molecule	3.44
RCHY1	Ring Finger And CHY Zinc Finger Domain Containing 1	3.44
ALDH1L2	Aldehyde Dehydrogenase 1 Family Member L2	3.44
GTF2H4	General Transcription Factor IIH Subunit 4	3.44
CEP120	Centrosomal Protein 120	3.44
CEP290	Centrosomal Protein 290	3.44
CD69	CD69 Molecule	3.44
PEX13	Peroxisomal Biogenesis Factor 13	3.44
SLC45A2	Solute Carrier Family 45 Member 2	3.44
TOX	Thymocyte Selection Associated High Mobility Group Box	3.44
TAF5L	TATA-Box Binding Protein Associated Factor 5 Like	3.44
KLK7	Kallikrein Related Peptidase 7	3.44
KIR3DL1	Killer Cell Immunoglobulin Like Receptor, Three Ig Domains And Long Cytoplasmic Tail 1	3.44
OXT	Oxytocin/Neurophysin I Prepropeptide	3.44
DYNC1I1	Dynein Cytoplasmic 1 Intermediate Chain 1	3.44
HOXC4	Homeobox C4	3.44
GTF2H2	General Transcription Factor IIH Subunit 2	3.44
SLC15A2	Solute Carrier Family 15 Member 2	3.44
SUPT5H	SPT5 Homolog, DSIF Elongation Factor Subunit	3.44
KANK2	KN Motif And Ankyrin Repeat Domains 2	3.44
MYCBP2	MYC Binding Protein 2	3.44
CD1B	CD1b Molecule	3.44
CD1C	CD1c Molecule	3.44
ITGAE	Integrin Subunit Alpha E	3.44

RECQL5	RecQ Like Helicase 5	3.44
WLS	Wnt Ligand Secretion Mediator	3.44
CCL22	C-C Motif Chemokine Ligand 22	3.44
PES1	Pescadillo Ribosomal Biogenesis Factor 1	3.44
CCR10	C-C Motif Chemokine Receptor 10	3.44
ACKR2	Atypical Chemokine Receptor 2	3.44
ACOT9	Acyl-CoA Thioesterase 9	3.44
RMI1	RecQ Mediated Genome Instability 1	3.44
DEFB4A	Defensin Beta 4A	3.44
DCLK3	Doublecortin Like Kinase 3	3.44
SLC16A12	Solute Carrier Family 16 Member 12	3.44
SLC15A3	Solute Carrier Family 15 Member 3	3.44
IQSEC2	IQ Motif And Sec7 Domain ArfGEF 2	3.44
TRAPPC10	Trafficking Protein Particle Complex 10	3.44
IL37	Interleukin 37	3.44
CCL18	C-C Motif Chemokine Ligand 18	3.44
AMELX	Amelogenin X-Linked	3.44
NOTUM	Notum, Palmitoleoyl-Protein Carboxylesterase	3.44
SNRPD3	Small Nuclear Ribonucleoprotein D3 Polypeptide	3.44
LAD1	Ladinin 1	3.44
TSPYL2	TSPY Like 2	3.44
PIF1	PIF1 5'-To-3' DNA Helicase	3.44
HOXC12	Homeobox C12	3.44
RNPC3	RNA Binding Region (RNP1, RRM) Containing 3	3.44
EMC1	ER Membrane Protein Complex Subunit 1	3.44
NELFCD	Negative Elongation Factor Complex Member C/D	3.44
SPATA17	Spermatogenesis Associated 17	3.44
RARS1	Arginyl-TRNA Synthetase 1	3.44
NAA25	N-Alpha-Acetyltransferase 25, NatB Auxiliary Subunit	3.44
KRT38	Keratin 38	3.44
GTSF1	Gametocyte Specific Factor 1	3.44
PRDM10	PR/SET Domain 10	3.44
R3HDM1	R3H Domain Containing 1	3.44
CWC15	CWC15 Spliceosome Associated Protein Homolog	3.44
OR3A3	Olfactory Receptor Family 3 Subfamily A Member 3	3.44
KRT27	Keratin 27	3.44
ZNF500	Zinc Finger Protein 500	3.44
FAM71D	Family With Sequence Similarity 71 Member D	3.44
OR12D3	Olfactory Receptor Family 12 Subfamily D Member 3	3.44
PWWP2B	PWWP Domain Containing 2B	3.44
C12orf40	Chromosome 12 Open Reading Frame 40	3.44

DEFB103B	Defensin Beta 103B	3.44
PRSS37	Serine Protease 37	3.44
CARNMT1	Carnosine N-Methyltransferase 1	3.44
LRP5L	LDL Receptor Related Protein 5 Like	3.44
OR13H1	Olfactory Receptor Family 13 Subfamily H Member 1	3.44
TMEM251	Transmembrane Protein 251	3.44
TMPRSS11F	Transmembrane Serine Protease 11F	3.44
FAM221B	Family With Sequence Similarity 221 Member B	3.44
KRTAP4-8	Keratin Associated Protein 4-8	3.44
RETREG3	Reticulophagy Regulator Family Member 3	3.44
MIR34A	MicroRNA 34a	3.44
HOXC-AS2	HOXC Cluster Antisense RNA 2	3.44
TRB	T Cell Receptor Beta Locus	3.44
MIR4647	MicroRNA 4647	3.44
MIR3689C	MicroRNA 3689c	3.44
ARCI9	Ichthyosis, Congenital, Autosomal Recessive 9	3.44
LOC112577531	Sharpr-MPRA Regulatory Region 5904	3.44
C2	Complement C2	3.41
SLAMF6	SLAM Family Member 6	3.41
SNRPD1	Small Nuclear Ribonucleoprotein D1 Polypeptide	3.41
TNFSF13	TNF Superfamily Member 13	3.3
EGFR	Epidermal Growth Factor Receptor	3.27
ALPL	Alkaline Phosphatase, Biomineralization Associated	3.22
CDKN1B	Cyclin Dependent Kinase Inhibitor 1B	3.22
CTSL	Cathepsin L	3.22
IFIH1	Interferon Induced With Helicase C Domain 1	3.14
CYP24A1	Cytochrome P450 Family 24 Subfamily A Member 1	3.14
MAPT	Microtubule Associated Protein Tau	3.12
ABCB6	ATP Binding Cassette Subfamily B Member 6 (Langereis Blood Group)	3.12
PRDM1	PR/SET Domain 1	3.12
TUBA4A	Tubulin Alpha 4a	3.12
ITPR2	Inositol 1,4,5-Trisphosphate Receptor Type 2	3.12
RAD23B	RAD23 Homolog B, Nucleotide Excision Repair Protein	3.12
WARS2	Tryptophanyl TRNA Synthetase 2, Mitochondrial	3.12
PREP	Prolyl Endopeptidase	3.12
WNT6	Wnt Family Member 6	3.12
MBNL1	Muscleblind Like Splicing Regulator 1	3.12
PRDM8	PR/SET Domain 8	3.12
MIR629	MicroRNA 629	3.12
LINC01432	Long Intergenic Non-Protein Coding RNA 1432	3.12

KDR	Kinase Insert Domain Receptor	3.08
ITGA6	Integrin Subunit Alpha 6	3.08
EPO	Erythropoietin	3.08
MAPK1	Mitogen-Activated Protein Kinase 1	3.02
BCR	BCR Activator Of RhoGEF And GTPase	3
PROS1	Protein S	3
SLC27A4	Solute Carrier Family 27 Member 4	3
SLC19A1	Solute Carrier Family 19 Member 1	3
TACR1	Tachykinin Receptor 1	3
ABCD3	ATP Binding Cassette Subfamily D Member 3	3
SSB	Small RNA Binding Exonuclease Protection Factor La	3
RO60	Ro60, Y RNA Binding Protein	3
TPMT	Thiopurine S-Methyltransferase	2.98
ZNF462	Zinc Finger Protein 462	2.98
NTF3	Neurotrophin 3	2.98
CCDC88B	Coiled-Coil Domain Containing 88B	2.98
HDAC9	Histone Deacetylase 9	2.88
SMAD3	SMAD Family Member 3	2.83
AKT2	AKT Serine/Threonine Kinase 2	2.81
MTOR	Mechanistic Target Of Rapamycin Kinase	2.81
PIK3CD	Phosphatidylinositol-4,5-Bisphosphate 3-Kinase Catalytic Subunit Delta	2.81
CCND1	Cyclin D1	2.81
HMOX1	Heme Oxygenase 1	2.81
MMP9	Matrix Metallopeptidase 9	2.81
FGFR4	Fibroblast Growth Factor Receptor 4	2.81
PRKG1	Protein Kinase CGMP-Dependent 1	2.81
SRC	SRC Proto-Oncogene, Non-Receptor Tyrosine Kinase	2.81
TGFBR2	Transforming Growth Factor Beta Receptor 2	2.81
CASP1	Caspase 1	2.81
BRCA1	BRCA1 DNA Repair Associated	2.81
CDKN1A	Cyclin Dependent Kinase Inhibitor 1A	2.81
MYD88	MYD88 Innate Immune Signal Transduction Adaptor	2.81
RARA	Retinoic Acid Receptor Alpha	2.81
STAT6	Signal Transducer And Activator Of Transcription 6	2.81
PRKCA	Protein Kinase C Alpha	2.81
BMP4	Bone Morphogenetic Protein 4	2.81
BRCA2	BRCA2 DNA Repair Associated	2.81
GNAQ	G Protein Subunit Alpha Q	2.81
SERPINA1	Serpin Family A Member 1	2.81
RB1	RB Transcriptional Corepressor 1	2.81

KCNH2	Potassium Voltage-Gated Channel Subfamily H Member 2	2.81
ADRB2	Adrenoceptor Beta 2	2.81
ABCA3	ATP Binding Cassette Subfamily A Member 3	2.81
ACACA	Acetyl-CoA Carboxylase Alpha	2.81
CYP3A4	Cytochrome P450 Family 3 Subfamily A Member 4	2.81
CREB1	CAMP Responsive Element Binding Protein 1	2.81
CSK	C-Terminal Src Kinase	2.81
CXCR2	C-X-C Motif Chemokine Receptor 2	2.81
HPRT1	Hypoxanthine Phosphoribosyltransferase 1	2.81
SERPINC1	Serpin Family C Member 1	2.81
RPS19	Ribosomal Protein S19	2.81
PI4KA	Phosphatidylinositol 4-Kinase Alpha	2.81
PIK3CG	Phosphatidylinositol-4,5-Bisphosphate 3-Kinase Catalytic Subunit Gamma	2.81
GBA	Glucosylceramidase Beta	2.81
FAH	Fumarylacetoacetate Hydrolase	2.81
MECOM	MDS1 And EVI1 Complex Locus	2.81
ACADM	Acyl-CoA Dehydrogenase Medium Chain	2.81
HIF1A	Hypoxia Inducible Factor 1 Subunit Alpha	2.81
DDX58	DExD/H-Box Helicase 58	2.81
RYR1	Ryanodine Receptor 1	2.81
RPL5	Ribosomal Protein L5	2.81
ISG15	ISG15 Ubiquitin Like Modifier	2.81
TRAF3	TNF Receptor Associated Factor 3	2.81
TTN	Titin	2.81
TUBB1	Tubulin Beta 1 Class VI	2.81
TYR	Tyrosinase	2.81
IRF1	Interferon Regulatory Factor 1	2.81
ALAD	Aminolevulinate Dehydratase	2.81
GNAT1	G Protein Subunit Alpha Transducin 1	2.81
ANXA5	Annexin A5	2.81
ALPP	Alkaline Phosphatase, Placental	2.81
FURIN	Furin, Paired Basic Amino Acid Cleaving Enzyme	2.81
FXN	Frataxin	2.81
MYBPC3	Myosin Binding Protein C3	2.81
LIG1	DNA Ligase 1	2.81
UNG	Uracil DNA Glycosylase	2.81
RASGRP1	RAS Guanyl Releasing Protein 1	2.81
DAG1	Dystroglycan 1	2.81
ALAS2	5'-Aminolevulinate Synthase 2	2.81
GALNT3	Polypeptide N-Acetylgalactosaminyltransferase 3	2.81

LRP2	LDL Receptor Related Protein 2	2.81
FCER2	Fc Fragment Of IgE Receptor II	2.81
ALDH3A2	Aldehyde Dehydrogenase 3 Family Member A2	2.81
ACHE	Acetylcholinesterase (Cartwright Blood Group)	2.81
ACOX1	Acyl-CoA Oxidase 1	2.81
ABCC6	ATP Binding Cassette Subfamily C Member 6	2.81
ADAMTS13	ADAM Metallopeptidase With Thrombospondin Type 1 Motif 13	2.81
ACAT2	Acetyl-CoA Acetyltransferase 2	2.81
MGLL	Monoglyceride Lipase	2.81
C1QA	Complement C1q A Chain	2.81
CYP2R1	Cytochrome P450 Family 2 Subfamily R Member 1	2.81
HADHA	Hydroxyacyl-CoA Dehydrogenase Trifunctional Multienzyme Complex Subunit Alpha	2.81
HBB	Hemoglobin Subunit Beta	2.81
MSX1	Msh Homeobox 1	2.81
RPL10	Ribosomal Protein L10	2.81
CD38	CD38 Molecule	2.81
PCCA	Propionyl-CoA Carboxylase Subunit Alpha	2.81
SLC6A8	Solute Carrier Family 6 Member 8	2.81
PHYH	Phytanoyl-CoA 2-Hydroxylase	2.81
RAD54L	RAD54 Like	2.81
CYP26B1	Cytochrome P450 Family 26 Subfamily B Member 1	2.81
DLL1	Delta Like Canonical Notch Ligand 1	2.81
ITGAL	Integrin Subunit Alpha L	2.81
KLF4	Kruppel Like Factor 4	2.81
C1QB	Complement C1q B Chain	2.81
C1QC	Complement C1q C Chain	2.81
LRAT	Lecithin Retinol Acyltransferase	2.81
AGER	Advanced Glycosylation End-Product Specific Receptor	2.81
MCCC1	Methylcrotonoyl-CoA Carboxylase 1	2.81
ABCA4	ATP Binding Cassette Subfamily A Member 4	2.81
ADCY2	Adenylate Cyclase 2	2.81
LOX	Lysyl Oxidase	2.81
CX3CR1	C-X3-C Motif Chemokine Receptor 1	2.81
CTSG	Cathepsin G	2.81
CR1	Complement C3b/C4b Receptor 1 (Knops Blood Group)	2.81
CST3	Cystatin C	2.81
CSTA	Cystatin A	2.81
HOXA1	Homeobox A1	2.81
ETFDH	Electron Transfer Flavoprotein Dehydrogenase	2.81
HLA-G	Major Histocompatibility Complex, Class I, G	2.81

HMBS	Hydroxymethylbilane Synthase	2.81
CKM	Creatine Kinase, M-Type	2.81
HABP2	Hyaluronan Binding Protein 2	2.81
MYO5B	Myosin VB	2.81
CD274	CD274 Molecule	2.81
GAS6	Growth Arrest Specific 6	2.81
IL10RB	Interleukin 10 Receptor Subunit Beta	2.81
SOCS3	Suppressor Of Cytokine Signaling 3	2.81
SNRPN	Small Nuclear Ribonucleoprotein Polypeptide N	2.81
SAG	S-Antigen Visual Arrestin	2.81
PLIN1	Perilipin 1	2.81
OCRL	OCRL Inositol Polyphosphate-5-Phosphatase	2.81
SLC34A1	Solute Carrier Family 34 Member 1	2.81
SLC25A15	Solute Carrier Family 25 Member 15	2.81
TNFRSF13C	TNF Receptor Superfamily Member 13C	2.81
RBP4	Retinol Binding Protein 4	2.81
KL	Klotho	2.81
CYP26A1	Cytochrome P450 Family 26 Subfamily A Member 1	2.81
ORAI1	ORAI Calcium Release-Activated Calcium Modulator 1	2.81
ABCB7	ATP Binding Cassette Subfamily B Member 7	2.81
ARF4	ADP Ribosylation Factor 4	2.81
FBL	Fibrillarin	2.81
ALOX12	Arachidonate 12-Lipoxygenase, 12S Type	2.81
AGPS	Alkylglycerone Phosphate Synthase	2.81
ADAMTS18	ADAM Metallopeptidase With Thrombospondin Type 1 Motif 18	2.81
ACAA2	Acetyl-CoA Acyltransferase 2	2.81
CRABP2	Cellular Retinoic Acid Binding Protein 2	2.81
CPOX	Coproporphyrinogen Oxidase	2.81
GC	GC Vitamin D Binding Protein	2.81
CMA1	Chymase 1	2.81
HAVCR2	Hepatitis A Virus Cellular Receptor 2	2.81
CHI3L1	Chitinase 3 Like 1	2.81
CHIT1	Chitinase 1	2.81
DGAT2	Diacylglycerol O-Acyltransferase 2	2.81
DNM3	Dynamin 3	2.81
NAT2	N-Acetyltransferase 2	2.81
CD2AP	CD2 Associated Protein	2.81
CD70	CD70 Molecule	2.81
HLA-DPB1	Major Histocompatibility Complex, Class II, DP Beta 1	2.81
PEX19	Peroxisomal Biogenesis Factor 19	2.81
IL18R1	Interleukin 18 Receptor 1	2.81

SFRP4	Secreted Frizzled Related Protein 4	2.81
S100A9	S100 Calcium Binding Protein A9	2.81
SMAD7	SMAD Family Member 7	2.81
PLIN2	Perilipin 2	2.81
TNFRSF25	TNF Receptor Superfamily Member 25	2.81
UGCG	UDP-Glucose Ceramide Glucosyltransferase	2.81
TAC1	Tachykinin Precursor 1	2.81
RAB2A	RAB2A, Member RAS Oncogene Family	2.81
ITM2B	Integral Membrane Protein 2B	2.81
TXNL4A	Thioredoxin Like 4A	2.81
DNASE1L3	Deoxyribonuclease 1 Like 3	2.81
FANCM	FA Complementation Group M	2.81
APOL1	Apolipoprotein L1	2.81
CX3CL1	C-X3-C Motif Chemokine Ligand 1	2.81
CYP4F3	Cytochrome P450 Family 4 Subfamily F Member 3	2.81
CLCN5	Chloride Voltage-Gated Channel 5	2.81
RFXANK	Regulatory Factor X Associated Ankyrin Containing Protein	2.81
HBA2	Hemoglobin Subunit Alpha 2	2.81
PEX3	Peroxisomal Biogenesis Factor 3	2.81
INPP4A	Inositol Polyphosphate-4-Phosphatase Type I A	2.81
TNFRSF18	TNF Receptor Superfamily Member 18	2.81
KLK6	Kallikrein Related Peptidase 6	2.81
STX3	Syntaxin 3	2.81
GALNT11	Polypeptide N-Acetylgalactosaminyltransferase 11	2.81
GJA3	Gap Junction Protein Alpha 3	2.81
GJC2	Gap Junction Protein Gamma 2	2.81
CD164	CD164 Molecule	2.81
FCN3	Ficolin 3	2.81
ALOX15B	Arachidonate 15-Lipoxygenase Type B	2.81
ABCA2	ATP Binding Cassette Subfamily A Member 2	2.81
CXCL13	C-X-C Motif Chemokine Ligand 13	2.81
CRABP1	Cellular Retinoic Acid Binding Protein 1	2.81
CAMP	Cathelicidin Antimicrobial Peptide	2.81
HAVCR1	Hepatitis A Virus Cellular Receptor 1	2.81
CHKA	Choline Kinase Alpha	2.81
CXCR5	C-X-C Motif Chemokine Receptor 5	2.81
PEX10	Peroxisomal Biogenesis Factor 10	2.81
PEX26	Peroxisomal Biogenesis Factor 26	2.81
IL21R	Interleukin 21 Receptor	2.81
SHOC2	SHOC2 Leucine Rich Repeat Scaffold Protein	2.81
SAR1A	Secretion Associated Ras Related GTPase 1A	2.81

PLIN3	Perilipin 3	2.81
SRSF1	Serine And Arginine Rich Splicing Factor 1	2.81
U2AF1	U2 Small Nuclear RNA Auxiliary Factor 1	2.81
TERF1	Telomeric Repeat Binding Factor 1	2.81
PTX3	Pentraxin 3	2.81
POLI	DNA Polymerase Iota	2.81
HSPA4	Heat Shock Protein Family A (Hsp70) Member 4	2.81
TFF3	Trefoil Factor 3	2.81
TGM5	Transglutaminase 5	2.81
GHRH	Growth Hormone Releasing Hormone	2.81
FCN2	Ficolin 2	2.81
CXCL11	C-X-C Motif Chemokine Ligand 11	2.81
CYBRD1	Cytochrome B Reductase 1	2.81
CKAP5	Cytoskeleton Associated Protein 5	2.81
GRHL2	Grainyhead Like Transcription Factor 2	2.81
EME1	Essential Meiotic Structure-Specific Endonuclease 1	2.81
EIF6	Eukaryotic Translation Initiation Factor 6	2.81
HLA-DPA1	Major Histocompatibility Complex, Class II, DP Alpha 1	2.81
SNRPA	Small Nuclear Ribonucleoprotein Polypeptide A	2.81
UBA6	Ubiquitin Like Modifier Activating Enzyme 6	2.81
UPF1	UPF1 RNA Helicase And ATPase	2.81
STRA6	Signaling Receptor And Transporter Of Retinol STRA6	2.81
RBP3	Retinol Binding Protein 3	2.81
TERF2	Telomeric Repeat Binding Factor 2	2.81
UTRN	Utrophin	2.81
CCRL2	C-C Motif Chemokine Receptor Like 2	2.81
ATE1	Arginyltransferase 1	2.81
ABCA6	ATP Binding Cassette Subfamily A Member 6	2.81
ABCA8	ATP Binding Cassette Subfamily A Member 8	2.81
ADGRG1	Adhesion G Protein-Coupled Receptor G1	2.81
FRAS1	Fraser Extracellular Matrix Complex Subunit 1	2.81
LY9	Lymphocyte Antigen 9	2.81
DOLK	Dolichol Kinase	2.81
RPGRIP1L	RPGRIP1 Like	2.81
PEX12	Peroxisomal Biogenesis Factor 12	2.81
IL23A	Interleukin 23 Subunit Alpha	2.81
IFIT3	Interferon Induced Protein With Tetratricopeptide Repeats 3	2.81
NIPA1	NIPA Magnesium Transporter 1	2.81
SLX4	SLX4 Structure-Specific Endonuclease Subunit	2.81
SLC25A17	Solute Carrier Family 25 Member 17	2.81
SRSF6	Serine And Arginine Rich Splicing Factor 6	2.81

LALBA	Lactalbumin Alpha	2.81
CD1E	CD1e Molecule	2.81
ZFYVE26	Zinc Finger FYVE-Type Containing 26	2.81
WAC	WW Domain Containing Adaptor With Coiled-Coil	2.81
GIMAP4	GTPase, IMAP Family Member 4	2.81
RHOJ	Ras Homolog Family Member J	2.81
HNRNPAB	Heterogeneous Nuclear Ribonucleoprotein A/B	2.81
CMPK2	Cytidine/Uridine Monophosphate Kinase 2	2.81
IL17D	Interleukin 17D	2.81
PEX16	Peroxisomal Biogenesis Factor 16	2.81
SUMO4	Small Ubiquitin Like Modifier 4	2.81
MCM10	Minichromosome Maintenance 10 Replication Initiation Factor	2.81
CAPN12	Calpain 12	2.81
ADPRH	ADP-Ribosylarginine Hydrolase	2.81
ABHD4	Abhydrolase Domain Containing 4, N-Acyl Phospholipase B	2.81
DSPP	Dentin Sialophosphoprotein	2.81
DYNLT1	Dynein Light Chain Tctex-Type 1	2.81
IBSP	Integrin Binding Sialoprotein	2.81
RPL38	Ribosomal Protein L38	2.81
TRIM39	Tripartite Motif Containing 39	2.81
TRUB1	TruB Pseudouridine Synthase Family Member 1	2.81
LIN9	Lin-9 DREAM MuvB Core Complex Component	2.81
SYNM	Synemin	2.81
TCEA2	Transcription Elongation Factor A2	2.81
KLK14	Kallikrein Related Peptidase 14	2.81
NHS	NHS Actin Remodeling Regulator	2.81
THRSP	Thyroid Hormone Responsive	2.81
MBOAT2	Membrane Bound O-Acyltransferase Domain Containing 2	2.81
BLOC1S1	Biogenesis Of Lysosomal Organelles Complex 1 Subunit 1	2.81
GIMAP5	GTPase, IMAP Family Member 5	2.81
ERAS	ES Cell Expressed Ras	2.81
CD52	CD52 Molecule	2.81
NIPA2	NIPA Magnesium Transporter 2	2.81
KIR2DL1	Killer Cell Immunoglobulin Like Receptor, Two Ig Domains And Long Cytoplasmic Tail 1	2.81
ZNF750	Zinc Finger Protein 750	2.81
ACKR4	Atypical Chemokine Receptor 4	2.81
GIMAP2	GTPase, IMAP Family Member 2	2.81
EPSTI1	Epithelial Stromal Interaction 1	2.81
RGS9BP	Regulator Of G Protein Signaling 9 Binding Protein	2.81
CSN3	Casein Kappa	2.81

PEX5L	Peroxisomal Biogenesis Factor 5 Like	2.81
NPW	Neuropeptide W	2.81
SOX7	SRY-Box Transcription Factor 7	2.81
ENOSF1	Enolase Superfamily Member 1	2.81
PNPLA7	Patatin Like Phospholipase Domain Containing 7	2.81
NUDT10	Nudix Hydrolase 10	2.81
TUT1	Terminal Uridylyl Transferase 1, U6 SnRNA-Specific	2.81
UNK	Unk Zinc Finger	2.81
MTHFSD	Methenyltetrahydrofolate Synthetase Domain Containing	2.81
SCAF4	SR-Related CTD Associated Factor 4	2.81
IARS1	Isoleucyl-TRNA Synthetase 1	2.81
UVSSA	UV Stimulated Scaffold Protein A	2.81
LMLN	Leishmanolysin Like Peptidase	2.81
ACTRT3	Actin Related Protein T3	2.81
ADA2	Adenosine Deaminase 2	2.81
SNU13	Small Nuclear Ribonucleoprotein 13	2.81
NIPAL2	NIPA Like Domain Containing 2	2.81
PNPLA5	Patatin Like Phospholipase Domain Containing 5	2.81
TTC7B	Tetratricopeptide Repeat Domain 7B	2.81
KRT72	Keratin 72	2.81
AKNAD1	AKNA Domain Containing 1	2.81
LRRC14	Leucine Rich Repeat Containing 14	2.81
NAV3	Neuron Navigator 3	2.81
CNTD1	Cyclin N-Terminal Domain Containing 1	2.81
PRR16	Proline Rich 16	2.81
PLIN5	Perilipin 5	2.81
SHQ1	SHQ1, H/ACA Ribonucleoprotein Assembly Factor	2.81
JRKL	JRK Like	2.81
SYNC	Syncoilin, Intermediate Filament Protein	2.81
EFR3B	EFR3 Homolog B	2.81
ZFAND4	Zinc Finger AN1-Type Containing 4	2.81
VNN3	Vanin 3	2.81
CCDC180	Coiled-Coil Domain Containing 180	2.81
ABHD14A	Abhydrolase Domain Containing 14A	2.81
CWF19L2	CWF19 Like Cell Cycle Control Factor 2	2.81
PLPBP	Pyridoxal Phosphate Binding Protein	2.81
ISX	Intestine Specific Homeobox	2.81
STN1	STN1 Subunit Of CST Complex	2.81
R3HCC1L	R3H Domain And Coiled-Coil Containing 1 Like	2.81
H2AC20	H2A Clustered Histone 20	2.81
CGB3	Chorionic Gonadotropin Subunit Beta 3	2.81

H2BC21	H2B Clustered Histone 21	2.81
MT-ATP6	Mitochondrially Encoded ATP Synthase Membrane Subunit 6	2.81
OR2T1	Olfactory Receptor Family 2 Subfamily T Member 1	2.81
KPRP	Keratinocyte Proline Rich Protein	2.81
CLEC2A	C-Type Lectin Domain Family 2 Member A	2.81
OR2M2	Olfactory Receptor Family 2 Subfamily M Member 2	2.81
ABHD16B	Abhydrolase Domain Containing 16B	2.81
ANKRD20A1	Ankyrin Repeat Domain 20 Family Member A1	2.81
DEFB103A	Defensin Beta 103A	2.81
IL17REL	Interleukin 17 Receptor E Like	2.81
SPRR2B	Small Proline Rich Protein 2B	2.81
ERICH6B	Glutamate Rich 6B	2.81
C1orf141	Chromosome 1 Open Reading Frame 141	2.81
OR2T12	Olfactory Receptor Family 2 Subfamily T Member 12	2.81
UPK3B	Uroplakin 3B	2.81
RAB40AL	RAB40A Like	2.81
GPR33	G Protein-Coupled Receptor 33	2.81
LCE5A	Late Cornified Envelope 5A	2.81
TMEM244	Transmembrane Protein 244	2.81
MIR125A	MicroRNA 125a	2.81
MIR200C	MicroRNA 200c	2.81
MIRLET7A1	MicroRNA Let-7a-1	2.81
MIR142	MicroRNA 142	2.81
MIR199A1	MicroRNA 199a-1	2.81
MIR574	MicroRNA 574	2.81
SCARNA13	Small Cajal Body-Specific RNA 13	2.81
SPATA41	Spermatogenesis Associated 41	2.81
SNORA64	Small Nucleolar RNA, H/ACA Box 64	2.81
RNU6ATAC	RNA, U6atac Small Nuclear (U12-Dependent Splicing)	2.81
MIR493	MicroRNA 493	2.81
SNORA75	Small Nucleolar RNA, H/ACA Box 75	2.81
C4B_2	Complement Component 4B (Chido Blood Group), Copy 2	2.81
SCARNA23	Small Cajal Body-Specific RNA 23	2.81
DSG1-AS1	DSG1 Antisense RNA 1	2.81
MIR4451	MicroRNA 4451	2.81
MIR3671	MicroRNA 3671	2.81
MIR3975	MicroRNA 3975	2.81
SNHG31	Small Nucleolar RNA Host Gene 31	2.81
LOC102724404	Uncharacterized LOC102724404	2.81
THM	Thymoma	2.81
LOC110806306	Telomerase RNA Component (TERC) Promoter	2.81

ECTD7	Ectodermal Dysplasia 7, Hair/Nail Type	2.81
LI5	Lamellar Ichthyosis 5	2.81
LOC110806263	TERT 5' Regulatory Region	2.81
LOC107882126	TGM1 Promoter Region	2.81
PRKD1	Protein Kinase D1	2.79
FYN	FYN Proto-Oncogene, Src Family Tyrosine Kinase	2.79
FGF10	Fibroblast Growth Factor 10	2.79
PGD	Phosphogluconate Dehydrogenase	2.79
CYP2C19	Cytochrome P450 Family 2 Subfamily C Member 19	2.79
SH2B3	SH2B Adaptor Protein 3	2.79
PTPN2	Protein Tyrosine Phosphatase Non-Receptor Type 2	2.79
RASA1	RAS P21 Protein Activator 1	2.79
HRH1	Histamine Receptor H1	2.79
IGFBP3	Insulin Like Growth Factor Binding Protein 3	2.79
ATG5	Autophagy Related 5	2.79
IL12B	Interleukin 12B	2.79
TET2	Tet Methylcytosine Dioxygenase 2	2.79
SOCS1	Suppressor Of Cytokine Signaling 1	2.79
FOXE1	Forkhead Box E1	2.79
RPS26	Ribosomal Protein S26	2.79
FANCI	FA Complementation Group I	2.79
EMG1	EMG1 N1-Specific Pseudouridine Methyltransferase	2.79
IL22	Interleukin 22	2.79
LSM2	LSM2 Homolog, U6 Small Nuclear RNA And MRNA Degradation Associated	2.79
MDC1	Mediator Of DNA Damage Checkpoint 1	2.79
RASA2	RAS P21 Protein Activator 2	2.79
HEPH	Hephaestin	2.79
CDC5L	Cell Division Cycle 5 Like	2.79
PMEL	Premelanosome Protein	2.79
ADAD1	Adenosine Deaminase Domain Containing 1	2.79
KIAA1217	KIAA1217	2.79
CEP83	Centrosomal Protein 83	2.79
DCLRE1B	DNA Cross-Link Repair 1B	2.79
PDZRN3	PDZ Domain Containing Ring Finger 3	2.79
KIAA1109	KIAA1109	2.79
SPIN2B	Spindlin Family Member 2B	2.79
SPIN2A	Spindlin Family Member 2A	2.79
DSG3	Desmoglein 3	2.75
CDH1	Cadherin 1	2.72
VIM	Vimentin	2.72

PTGS2	Prostaglandin-Endoperoxide Synthase 2	2.72
SMAD1	SMAD Family Member 1	2.66
GPT	Glutamic--Pyruvic Transaminase	2.66
SMAD5	SMAD Family Member 5	2.66
CTH	Cystathionine Gamma-Lyase	2.65
CACNB4	Calcium Voltage-Gated Channel Auxiliary Subunit Beta 4	2.65
TRIO	Trio Rho Guanine Nucleotide Exchange Factor	2.65
ALOX5AP	Arachidonate 5-Lipoxygenase Activating Protein	2.65
SERPINB5	Serpin Family B Member 5	2.65
CDH13	Cadherin 13	2.65
BCL9	BCL9 Transcription Coactivator	2.65
ME3	Malic Enzyme 3	2.65
CDH7	Cadherin 7	2.65
DENND1A	DENN Domain Containing 1A	2.65
PCDH15	Protocadherin Related 15	2.65
STAM2	Signal Transducing Adaptor Molecule 2	2.65
CAMKMT	Calmodulin-Lysine N-Methyltransferase	2.65
PRPF18	Pre-MRNA Processing Factor 18	2.65
LCORL	Ligand Dependent Nuclear Receptor Corepressor Like	2.65
KLHL25	Kelch Like Family Member 25	2.65
TMEM132C	Transmembrane Protein 132C	2.65
C3orf56	Chromosome 3 Open Reading Frame 56	2.65
RPL10AP9	Ribosomal Protein L10a Pseudogene 9	2.65
TWIST1	Twist Family BHLH Transcription Factor 1	2.64
SLC14A2	Solute Carrier Family 14 Member 2	2.64
SETBP1	SET Binding Protein 1	2.64
C1orf127	Chromosome 1 Open Reading Frame 127	2.64
SSPN	Sarcospan	2.64
SPPL2C	Signal Peptide Peptidase Like 2C	2.64
MAPT-AS1	MAPT Antisense RNA 1	2.64
DCN	Decorin	2.58
LAMB1	Laminin Subunit Beta 1	2.58
OSMR	Oncostatin M Receptor	2.58
NR3C2	Nuclear Receptor Subfamily 3 Group C Member 2	2.56
PLCD3	Phospholipase C Delta 3	2.44
PRKCE	Protein Kinase C Epsilon	2.39
PGR	Progesterone Receptor	2.39
IL6R	Interleukin 6 Receptor	2.39
LGMN	Legumain	2.39
CTSV	Cathepsin V	2.39
HMU	Hypotrichosis, Marie Unna Type	2.39

NCOA1	Nuclear Receptor Coactivator 1	2.39
XDH	Xanthine Dehydrogenase	2.38
LOC102723878	Uncharacterized LOC102723878	2.38
ERBB2	Erb-B2 Receptor Tyrosine Kinase 2	2.36
LEF1	Lymphoid Enhancer Binding Factor 1	2.31
FOXA2	Forkhead Box A2	2.3
MED4	Mediator Complex Subunit 4	2.3
RAET1M	Retinoic Acid Early Transcript 1M Pseudogene	2.3
KRT18P39	Keratin 18 Pseudogene 39	2.3
SERPINE1	Serpin Family E Member 1	2.25
ABCC2	ATP Binding Cassette Subfamily C Member 2	2.25
MC4R	Melanocortin 4 Receptor	2.25
RPL41P1	Ribosomal Protein L41 Pseudogene 1	2.22
FLT3	Fms Related Receptor Tyrosine Kinase 3	2.18
NGF	Nerve Growth Factor	2.18
PARP1	Poly(ADP-Ribose) Polymerase 1	2.18
UGT1A1	UDP Glucuronosyltransferase Family 1 Member A1	2.18
BDNF	Brain Derived Neurotrophic Factor	2.18
HSPA8	Heat Shock Protein Family A (Hsp70) Member 8	2.18
ENTPD1	Ectonucleoside Triphosphate Diphosphohydrolase 1	2.18
F3	Coagulation Factor III, Tissue Factor	2.18
IDO1	Indoleamine 2,3-Dioxygenase 1	2.18
HMGCR	3-Hydroxy-3-Methylglutaryl-CoA Reductase	2.18
NTF4	Neurotrophin 4	2.18
VIP	Vasoactive Intestinal Peptide	2.18
RNF216	Ring Finger Protein 216	2.18
SST	Somatostatin	2.18
DSC3	Desmocollin 3	2.18
HLA-E	Major Histocompatibility Complex, Class I, E	2.18
XRCC3	X-Ray Repair Cross Complementing 3	2.18
ABCD2	ATP Binding Cassette Subfamily D Member 2	2.18
PRKN	Parkin RBR E3 Ubiquitin Protein Ligase	2.18
PMP22	Peripheral Myelin Protein 22	2.18
PTMA	Prothymosin Alpha	2.18
IL31	Interleukin 31	2.18
UTP4	UTP4 Small Subunit Processome Component	2.18
PDGFRA	Platelet Derived Growth Factor Receptor Alpha	1.99
MAP2K1	Mitogen-Activated Protein Kinase Kinase 1	1.99
IGF1R	Insulin Like Growth Factor 1 Receptor	1.99
MAP2K2	Mitogen-Activated Protein Kinase Kinase 2	1.99
CHEK2	Checkpoint Kinase 2	1.99

MMP2	Matrix Metallopeptidase 2	1.99
RET	Ret Proto-Oncogene	1.99
CASP8	Caspase 8	1.99
MDM2	MDM2 Proto-Oncogene	1.99
FLT4	Fms Related Receptor Tyrosine Kinase 4	1.99
CTSD	Cathepsin D	1.99
PSEN1	Presenilin 1	1.99
RPS6KA3	Ribosomal Protein S6 Kinase A3	1.99
MAPK14	Mitogen-Activated Protein Kinase 14	1.99
MAP3K7	Mitogen-Activated Protein Kinase Kinase Kinase 7	1.99
CTSB	Cathepsin B	1.99
APP	Amyloid Beta Precursor Protein	1.99
CFTR	CF Transmembrane Conductance Regulator	1.99
MMP3	Matrix Metallopeptidase 3	1.99
MMP14	Matrix Metallopeptidase 14	1.99
GRIN2A	Glutamate Ionotropic Receptor NMDA Type Subunit 2A	1.99
CDC42	Cell Division Cycle 42	1.99
PLCG2	Phospholipase C Gamma 2	1.99
PTPN1	Protein Tyrosine Phosphatase Non-Receptor Type 1	1.99
ITK	IL2 Inducible T Cell Kinase	1.99
CASP7	Caspase 7	1.99
FTH1	Ferritin Heavy Chain 1	1.99
GLUD1	Glutamate Dehydrogenase 1	1.99
MAPK8	Mitogen-Activated Protein Kinase 8	1.99
APOE	Apolipoprotein E	1.99
EP300	E1A Binding Protein P300	1.99
AHCY	Adenosylhomocysteinase	1.99
EPHA4	EPH Receptor A4	1.99
BMPR1B	Bone Morphogenetic Protein Receptor Type 1B	1.99
BMPR2	Bone Morphogenetic Protein Receptor Type 2	1.99
MERTK	MER Proto-Oncogene, Tyrosine Kinase	1.99
ACVRL1	Activin A Receptor Like Type 1	1.99
FOS	Fos Proto-Oncogene, AP-1 Transcription Factor Subunit	1.99
MGMT	O-6-Methylguanine-DNA Methyltransferase	1.99
FN1	Fibronectin 1	1.99
MME	Membrane Metalloendopeptidase	1.99
CHEK1	Checkpoint Kinase 1	1.99
MMP13	Matrix Metallopeptidase 13	1.99
GRM1	Glutamate Metabotropic Receptor 1	1.99
NOTCH2	Notch Receptor 2	1.99
SCN5A	Sodium Voltage-Gated Channel Alpha Subunit 5	1.99

NFKBIA	NFKB Inhibitor Alpha	1.99
RPS6KA1	Ribosomal Protein S6 Kinase A1	1.99
PTH1R	Parathyroid Hormone 1 Receptor	1.99
RELA	RELA Proto-Oncogene, NF-KB Subunit	1.99
COL1A1	Collagen Type I Alpha 1 Chain	1.99
MSH6	MutS Homolog 6	1.99
SNCA	Synuclein Alpha	1.99
JAG1	Jagged Canonical Notch Ligand 1	1.99
LRRK2	Leucine Rich Repeat Kinase 2	1.99
FLNA	Filamin A	1.99
GRB2	Growth Factor Receptor Bound Protein 2	1.99
NOS2	Nitric Oxide Synthase 2	1.99
PLK1	Polo Like Kinase 1	1.99
PIK3R2	Phosphoinositide-3-Kinase Regulatory Subunit 2	1.99
JUN	Jun Proto-Oncogene, AP-1 Transcription Factor Subunit	1.99
KCNQ1	Potassium Voltage-Gated Channel Subfamily Q Member 1	1.99
GATA4	GATA Binding Protein 4	1.99
CACNA1C	Calcium Voltage-Gated Channel Subunit Alpha1 C	1.99
AXIN2	Axin 2	1.99
ANXA2	Annexin A2	1.99
ATP2A1	ATPase Sarcoplasmic/Endoplasmic Reticulum Ca2+ Transporting 1	1.99
MDH2	Malate Dehydrogenase 2	1.99
ADCY1	Adenylate Cyclase 1	1.99
GALK1	Galactokinase 1	1.99
CYP2D6	Cytochrome P450 Family 2 Subfamily D Member 6	1.99
GLA	Galactosidase Alpha	1.99
GSR	Glutathione-Disulfide Reductase	1.99
GSN	Gelsolin	1.99
CHRNA4	Cholinergic Receptor Nicotinic Alpha 4 Subunit	1.99
MLH1	MutL Homolog 1	1.99
MSH2	MutS Homolog 2	1.99
RPL11	Ribosomal Protein L11	1.99
NCF1	Neutrophil Cytosolic Factor 1	1.99
PPP2CA	Protein Phosphatase 2 Catalytic Subunit Alpha	1.99
PDE4D	Phosphodiesterase 4D	1.99
PDE3A	Phosphodiesterase 3A	1.99
NFE2L2	Nuclear Factor, Erythroid 2 Like 2	1.99
SLC12A6	Solute Carrier Family 12 Member 6	1.99
TNNI3	Troponin I3, Cardiac Type	1.99
TUBA1A	Tubulin Alpha 1a	1.99
KCNN4	Potassium Calcium-Activated Channel Subfamily N Member 4	1.99

STIM1	Stromal Interaction Molecule 1	1.99
TGM2	Transglutaminase 2	1.99
VRK1	VRK Serine/Threonine Kinase 1	1.99
VWF	Von Willebrand Factor	1.99
GHR	Growth Hormone Receptor	1.99
AQP3	Aquaporin 3 (Gill Blood Group)	1.99
BACE1	Beta-Secretase 1	1.99
FADD	Fas Associated Via Death Domain	1.99
FGA	Fibrinogen Alpha Chain	1.99
ATP7B	ATPase Copper Transporting Beta	1.99
FANCC	FA Complementation Group C	1.99
FANCD2	FA Complementation Group D2	1.99
ADSL	Adenylosuccinate Lyase	1.99
BLNK	B Cell Linker	1.99
FGF8	Fibroblast Growth Factor 8	1.99
EPHX1	Epoxide Hydrolase 1	1.99
LYZ	Lysozyme	1.99
CYBA	Cytochrome B-245 Alpha Chain	1.99
CYBB	Cytochrome B-245 Beta Chain	1.99
CYP1A1	Cytochrome P450 Family 1 Subfamily A Member 1	1.99
CTNNA1	Catenin Alpha 1	1.99
CDKN2B	Cyclin Dependent Kinase Inhibitor 2B	1.99
CD3E	CD3e Molecule	1.99
CD44	CD44 Molecule (Indian Blood Group)	1.99
CD55	CD55 Molecule (Cromer Blood Group)	1.99
HMOX2	Heme Oxygenase 2	1.99
COMP	Cartilage Oligomeric Matrix Protein	1.99
MITF	Melanocyte Inducing Transcription Factor	1.99
GRM5	Glutamate Metabotropic Receptor 5	1.99
GATA2	GATA Binding Protein 2	1.99
IKZF1	IKAROS Family Zinc Finger 1	1.99
PRNP	Prion Protein	1.99
SOS1	SOS Ras/Rac Guanine Nucleotide Exchange Factor 1	1.99
RYR2	Ryanodine Receptor 2	1.99
RRM1	Ribonucleotide Reductase Catalytic Subunit M1	1.99
NOG	Noggin	1.99
ENO2	Enolase 2	1.99
OGG1	8-Oxoguanine DNA Glycosylase	1.99
PKLR	Pyruvate Kinase L/R	1.99
SLC11A2	Solute Carrier Family 11 Member 2	1.99

PIK3C2A	Phosphatidylinositol-4-Phosphate 3-Kinase Catalytic Subunit Type 2 Alpha	1.99
UBE2N	Ubiquitin Conjugating Enzyme E2 N	1.99
IRF3	Interferon Regulatory Factor 3	1.99
IRS1	Insulin Receptor Substrate 1	1.99
TAB2	TGF-Beta Activated Kinase 1 (MAP3K7) Binding Protein 2	1.99
PTS	6-Pyruvoyltetrahydropterin Synthase	1.99
STAT2	Signal Transducer And Activator Of Transcription 2	1.99
NBN	Nibrin	1.99
MSX2	Msh Homeobox 2	1.99
KCNJ1	Potassium Inwardly Rectifying Channel Subfamily J Member 1	1.99
KCNJ11	Potassium Inwardly Rectifying Channel Subfamily J Member 11	1.99
USP7	Ubiquitin Specific Peptidase 7	1.99
USP9X	Ubiquitin Specific Peptidase 9 X-Linked	1.99
F9	Coagulation Factor IX	1.99
MASP1	Mannan Binding Lectin Serine Peptidase 1	1.99
LTA4H	Leukotriene A4 Hydrolase	1.99
FKBP1A	FKBP Prolyl Isomerase 1A	1.99
ACTN4	Actinin Alpha 4	1.99
CD46	CD46 Molecule	1.99
RIPK2	Receptor Interacting Serine/Threonine Kinase 2	1.99
CFI	Complement Factor I	1.99
DMD	Dystrophin	1.99
ELANE	Elastase, Neutrophil Expressed	1.99
RPS6KA2	Ribosomal Protein S6 Kinase A2	1.99
TNFRSF13B	TNF Receptor Superfamily Member 13B	1.99
L1CAM	L1 Cell Adhesion Molecule	1.99
LFNG	LFNG O-Fucosylpeptide 3-Beta-N-Acetylglucosaminyltransferase	1.99
TYRP1	Tyrosinase Related Protein 1	1.99
IRF7	Interferon Regulatory Factor 7	1.99
KDM6A	Lysine Demethylase 6A	1.99
ITGA3	Integrin Subunit Alpha 3	1.99
PSMA6	Proteasome 20S Subunit Alpha 6	1.99
DCTN1	Dynactin Subunit 1	1.99
CDK7	Cyclin Dependent Kinase 7	1.99
AFP	Alpha Fetoprotein	1.99
FGF17	Fibroblast Growth Factor 17	1.99
CAMK4	Calcium/Calmodulin Dependent Protein Kinase IV	1.99
GJA8	Gap Junction Protein Alpha 8	1.99
CCR2	C-C Motif Chemokine Receptor 2	1.99
GOT2	Glutamic-Oxaloacetic Transaminase 2	1.99

FANCL	FA Complementation Group L	1.99
MAP2K7	Mitogen-Activated Protein Kinase Kinase 7	1.99
ATRX	ATRX Chromatin Remodeler	1.99
F2RL1	F2R Like Trypsin Receptor 1	1.99
ATP7A	ATPase Copper Transporting Alpha	1.99
BMP1	Bone Morphogenetic Protein 1	1.99
ACVR2A	Activin A Receptor Type 2A	1.99
ADCY7	Adenylate Cyclase 7	1.99
CYP2E1	Cytochrome P450 Family 2 Subfamily E Member 1	1.99
CTNND1	Catenin Delta 1	1.99
CTSC	Cathepsin C	1.99
HPD	4-Hydroxyphenylpyruvate Dioxygenase	1.99
EXTL3	Exostosin Like Glycosyltransferase 3	1.99
CFB	Complement Factor B	1.99
CFH	Complement Factor H	1.99
RPA1	Replication Protein A1	1.99
CD22	CD22 Molecule	1.99
CDK1	Cyclin Dependent Kinase 1	1.99
MOG	Myelin Oligodendrocyte Glycoprotein	1.99
COL5A1	Collagen Type V Alpha 1 Chain	1.99
NEFH	Neurofilament Heavy	1.99
NEK9	NIMA Related Kinase 9	1.99
HRH4	Histamine Receptor H4	1.99
SERPINH1	Serpin Family H Member 1	1.99
IFNAR1	Interferon Alpha And Beta Receptor Subunit 1	1.99
PAFAH1B1	Platelet Activating Factor Acetylhydrolase 1b Regulatory Subunit 1	1.99
PPP1CB	Protein Phosphatase 1 Catalytic Subunit Beta	1.99
PRODH	Proline Dehydrogenase 1	1.99
SOS2	SOS Ras/Rho Guanine Nucleotide Exchange Factor 2	1.99
PRF1	Perforin 1	1.99
SDC2	Syndecan 2	1.99
PML	PML Nuclear Body Scaffold	1.99
SLC27A2	Solute Carrier Family 27 Member 2	1.99
SLC29A2	Solute Carrier Family 29 Member 2	1.99
OAS1	2'-5'-Oligoadenylate Synthetase 1	1.99
TNFSF15	TNF Superfamily Member 15	1.99
LARS2	Leucyl-TRNA Synthetase 2, Mitochondrial	1.99
TNFRSF11A	TNF Receptor Superfamily Member 11a	1.99
TXN	Thioredoxin	1.99
UBE2A	Ubiquitin Conjugating Enzyme E2 A	1.99
UBE2B	Ubiquitin Conjugating Enzyme E2 B	1.99

UBE2D1	Ubiquitin Conjugating Enzyme E2 D1	1.99
RANBP2	RAN Binding Protein 2	1.99
TBX5	T-Box Transcription Factor 5	1.99
TEC	Tec Protein Tyrosine Kinase	1.99
STEAP3	STEAP3 Metalloreductase	1.99
TRPM6	Transient Receptor Potential Cation Channel Subfamily M Member 6	1.99
LAT	Linker For Activation Of T Cells	1.99
MYH6	Myosin Heavy Chain 6	1.99
MYO6	Myosin VI	1.99
POLD1	DNA Polymerase Delta 1, Catalytic Subunit	1.99
P2RX7	Purinergic Receptor P2X 7	1.99
ITCH	Itchy E3 Ubiquitin Protein Ligase	1.99
TGFBI	Transforming Growth Factor Beta Induced	1.99
TGIF1	TGFB Induced Factor Homeobox 1	1.99
VAPB	VAMP Associated Protein B And C	1.99
LMAN1	Lectin, Mannose Binding 1	1.99
ALAS1	5'-Aminolevulinate Synthase 1	1.99
CARD9	Caspase Recruitment Domain Family Member 9	1.99
CANX	Calnexin	1.99
APTX	Aprataxin	1.99
AOC3	Amine Oxidase Copper Containing 3	1.99
ALS2	Alsin Rho Guanine Nucleotide Exchange Factor ALS2	1.99
AMPH	Amphiphysin	1.99
MCOLN1	Mucolipin 1	1.99
BMX	BMX Non-Receptor Tyrosine Kinase	1.99
BCKDHA	Branched Chain Keto Acid Dehydrogenase E1 Subunit Alpha	1.99
ACO1	Aconitase 1	1.99
ADH4	Alcohol Dehydrogenase 4 (Class II), Pi Polypeptide	1.99
GADD45A	Growth Arrest And DNA Damage Inducible Alpha	1.99
FMR1	FMRP Translational Regulator 1	1.99
FUT2	Fucosyltransferase 2	1.99
MAD2L2	Mitotic Arrest Deficient 2 Like 2	1.99
APOA2	Apolipoprotein A2	1.99
RHAG	Rh Associated Glycoprotein	1.99
CISH	Cytokine Inducible SH2 Containing Protein	1.99
CLCN1	Chloride Voltage-Gated Channel 1	1.99
CLCNKB	Chloride Voltage-Gated Channel Kb	1.99
HDC	Histidine Decarboxylase	1.99
CFD	Complement Factor D	1.99
DNAJB1	DnaJ Heat Shock Protein Family (Hsp40) Member B1	1.99

RPL15	Ribosomal Protein L15	1.99
MYL3	Myosin Light Chain 3	1.99
RPA2	Replication Protein A2	1.99
GPNMB	Glycoprotein Nmb	1.99
EHHADH	Enoyl-CoA Hydratase And 3-Hydroxyacyl CoA Dehydrogenase	1.99
SFTPD	Surfactant Protein D	1.99
SGSH	N-Sulfoglucosamine Sulfohydrolase	1.99
PADI4	Peptidyl Arginine Deiminase 4	1.99
SP1	Sp1 Transcription Factor	1.99
SDHD	Succinate Dehydrogenase Complex Subunit D	1.99
RPL35A	Ribosomal Protein L35a	1.99
NFATC4	Nuclear Factor Of Activated T Cells 4	1.99
NKX2-5	NK2 Homeobox 5	1.99
RPS10	Ribosomal Protein S10	1.99
RPS14	Ribosomal Protein S14	1.99
SLC6A6	Solute Carrier Family 6 Member 6	1.99
OCLN	Occludin	1.99
SLC46A1	Solute Carrier Family 46 Member 1	1.99
OGT	O-Linked N-Acetylglucosamine (GlcNAc) Transferase	1.99
OPTN	Optineurin	1.99
NTHL1	Nth Like DNA Glycosylase 1	1.99
NPPB	Natriuretic Peptide B	1.99
NR1I3	Nuclear Receptor Subfamily 1 Group I Member 3	1.99
PTPRN	Protein Tyrosine Phosphatase Receptor Type N	1.99
TOR1A	Torsin Family 1 Member A	1.99
TNFRSF17	TNF Receptor Superfamily Member 17	1.99
TDG	Thymine DNA Glycosylase	1.99
TMPRSS6	Transmembrane Serine Protease 6	1.99
TRIM33	Tripartite Motif Containing 33	1.99
LGALS3	Galectin 3	1.99
LIG3	DNA Ligase 3	1.99
PVR	PVR Cell Adhesion Molecule	1.99
KCNN3	Potassium Calcium-Activated Channel Subfamily N Member 3	1.99
RBBP8	RB Binding Protein 8, Endonuclease	1.99
REV3L	REV3 Like, DNA Directed Polymerase Zeta Catalytic Subunit	1.99
TAZ	Tafazzin	1.99
TLE1	TLE Family Member 1, Transcriptional Corepressor	1.99
PSMD4	Proteasome 26S Subunit, Non-ATPase 4	1.99
PRKAR2B	Protein Kinase CAMP-Dependent Type II Regulatory Subunit Beta	1.99
HUWE1	HECT, UBA And WWE Domain Containing E3 Ubiquitin Protein Ligase 1	1.99

PDE6D	Phosphodiesterase 6D	1.99
PDE4A	Phosphodiesterase 4A	1.99
RPE65	Retinoid Isomerohydrolase RPE65	1.99
MYO9B	Myosin IXB	1.99
CYP51A1	Cytochrome P450 Family 51 Subfamily A Member 1	1.99
PON3	Paraoxonase 3	1.99
THY1	Thy-1 Cell Surface Antigen	1.99
USP1	Ubiquitin Specific Peptidase 1	1.99
FXYD2	FXYD Domain Containing Ion Transport Regulator 2	1.99
GHRL	Ghrelin And Obestatin Prepropeptide	1.99
CCL20	C-C Motif Chemokine Ligand 20	1.99
CCL21	C-C Motif Chemokine Ligand 21	1.99
GJA4	Gap Junction Protein Alpha 4	1.99
GMPR	Guanosine Monophosphate Reductase	1.99
FABP2	Fatty Acid Binding Protein 2	1.99
MAPKAP1	MAPK Associated Protein 1	1.99
FIG4	FIG4 Phosphoinositide 5-Phosphatase	1.99
MAFB	MAF BZIP Transcription Factor B	1.99
FAR1	Fatty Acyl-CoA Reductase 1	1.99
ATG16L1	Autophagy Related 16 Like 1	1.99
LTF	Lactotransferrin	1.99
LZTR1	Leucine Zipper Like Transcription Regulator 1	1.99
FANCG	FA Complementation Group G	1.99
AMD1	Adenosylmethionine Decarboxylase 1	1.99
AMH	Anti-Mullerian Hormone	1.99
AIMP1	Aminoacyl TRNA Synthetase Complex Interacting Multifunctional Protein 1	1.99
ACD	ACD Shelterin Complex Subunit And Telomerase Recruitment Factor	1.99
ABCD4	ATP Binding Cassette Subfamily D Member 4	1.99
ADH1B	Alcohol Dehydrogenase 1B (Class I), Beta Polypeptide	1.99
ACAA1	Acetyl-CoA Acyltransferase 1	1.99
C5AR1	Complement C5a Receptor 1	1.99
FOXP2	Forkhead Box P2	1.99
APBB1	Amyloid Beta Precursor Protein Binding Family B Member 1	1.99
FCER1A	Fc Fragment Of IgE Receptor Ia	1.99
CXADR	CXADR Ig-Like Cell Adhesion Molecule	1.99
CXCR1	C-X-C Motif Chemokine Receptor 1	1.99
CRBN	Cereblon	1.99
GAP43	Growth Associated Protein 43	1.99
CDKL5	Cyclin Dependent Kinase Like 5	1.99
GTF2H1	General Transcription Factor IIH Subunit 1	1.99

HEY1	Hes Related Family BHLH Transcription Factor With YRPW Motif 1	1.99
COL11A1	Collagen Type XI Alpha 1 Chain	1.99
DPP6	Dipeptidyl Peptidase Like 6	1.99
DYSF	Dysferlin	1.99
E2F1	E2F Transcription Factor 1	1.99
CD209	CD209 Molecule	1.99
CD244	CD244 Molecule	1.99
DDX11	DEAD/H-Box Helicase 11	1.99
DDX41	DEAD-Box Helicase 41	1.99
CYSLTR1	Cysteinyl Leukotriene Receptor 1	1.99
EIF4A3	Eukaryotic Translation Initiation Factor 4A3	1.99
RPL26	Ribosomal Protein L26	1.99
CPN1	Carboxypeptidase N Subunit 1	1.99
PRSS3	Serine Protease 3	1.99
PRPF3	Pre-MRNA Processing Factor 3	1.99
NOP56	NOP56 Ribonucleoprotein	1.99
NPHP1	Nephrocystin 1	1.99
PANK2	Pantothenate Kinase 2	1.99
PPOX	Protoporphyrinogen Oxidase	1.99
IGFBP1	Insulin Like Growth Factor Binding Protein 1	1.99
SOX17	SRY-Box Transcription Factor 17	1.99
SDC1	Syndecan 1	1.99
RRAS	RAS Related	1.99
RPS6KA6	Ribosomal Protein S6 Kinase A6	1.99
SBDS	SBDS Ribosome Maturation Factor	1.99
RPS24	Ribosomal Protein S24	1.99
NMT1	N-Myristoyltransferase 1	1.99
POLR3A	RNA Polymerase III Subunit A	1.99
SLC33A1	Solute Carrier Family 33 Member 1	1.99
SEPSECS	Sep (O-Phosphoserine) TRNA:Sec (Selenocysteine) TRNA Synthase	1.99
TNFRSF4	TNF Receptor Superfamily Member 4	1.99
TNFRSF6B	TNF Receptor Superfamily Member 6b	1.99
TRADD	TNFRSF1A Associated Via Death Domain	1.99
LAP3	Leucine Aminopeptidase 3	1.99
LGALS1	Galectin 1	1.99
TRIM24	Tripartite Motif Containing 24	1.99
UBE2V2	Ubiquitin Conjugating Enzyme E2 V2	1.99
UQCRB	Ubiquinol-Cytochrome C Reductase Binding Protein	1.99
SYP	Synaptophysin	1.99
KCNN2	Potassium Calcium-Activated Channel Subfamily N Member 2	1.99

TAPBP	TAP Binding Protein	1.99
JAM3	Junctional Adhesion Molecule 3	1.99
TJP1	Tight Junction Protein 1	1.99
SRR	Serine Racemase	1.99
IREB2	Iron Responsive Element Binding Protein 2	1.99
SAMHD1	SAM And HD Domain Containing Deoxynucleoside Triphosphate Triphosphohydrolase 1	1.99
MSH3	MutS Homolog 3	1.99
POLL	DNA Polymerase Lambda	1.99
PPBP	Pro-Platelet Basic Protein	1.99
POT1	Protection Of Telomeres 1	1.99
ID1	Inhibitor Of DNA Binding 1, HLH Protein	1.99
KREMEN1	Kringle Containing Transmembrane Protein 1	1.99
KRT13	Keratin 13	1.99
UBC	Ubiquitin C	1.99
MAN2A1	Mannosidase Alpha Class 2A Member 1	1.99
LPIN2	Lipin 2	1.99
ARFGAP1	ADP Ribosylation Factor GTPase Activating Protein 1	1.99
BCL11B	BAF Chromatin Remodeling Complex Subunit BCL11B	1.99
ADH1A	Alcohol Dehydrogenase 1A (Class I), Alpha Polypeptide	1.99
ATXN10	Ataxin 10	1.99
CTSE	Cathepsin E	1.99
HBA1	Hemoglobin Subunit Alpha 1	1.99
CETN2	Centrin 2	1.99
DNTT	DNA Nucleotidylexotransferase	1.99
NEDD9	Neural Precursor Cell Expressed, Developmentally Down-Regulated 9	1.99
PFAS	Phosphoribosylformylglycinamidine Synthase	1.99
PRPF31	Pre-MRNA Processing Factor 31	1.99
PRPF4	Pre-MRNA Processing Factor 4	1.99
SECISBP2	SECIS Binding Protein 2	1.99
IFI16	Interferon Gamma Inducible Protein 16	1.99
PROCR	Protein C Receptor	1.99
SAA1	Serum Amyloid A1	1.99
RPS17	Ribosomal Protein S17	1.99
SLC30A10	Solute Carrier Family 30 Member 10	1.99
SLC30A8	Solute Carrier Family 30 Member 8	1.99
SLC35A3	Solute Carrier Family 35 Member A3	1.99
SPRY1	Sprouty RTK Signaling Antagonist 1	1.99
PIGK	Phosphatidylinositol Glycan Anchor Biosynthesis Class K	1.99
TRAF5	TNF Receptor Associated Factor 5	1.99
TRH	Thyrotropin Releasing Hormone	1.99

RARS2	Arginyl-TRNA Synthetase 2, Mitochondrial	1.99
TCOF1	Treacle Ribosome Biogenesis Factor 1	1.99
TERF2IP	TERF2 Interacting Protein	1.99
RAD23A	RAD23 Homolog A, Nucleotide Excision Repair Protein	1.99
PYY	Peptide YY	1.99
PROX1	Prospero Homeobox 1	1.99
S100A8	S100 Calcium Binding Protein A8	1.99
NAGS	N-Acetylglutamate Synthase	1.99
SNAP29	Synaptosome Associated Protein 29	1.99
PON2	Paraoxonase 2	1.99
RAD17	RAD17 Checkpoint Clamp Loader Component	1.99
STX16	Syntaxin 16	1.99
JAG2	Jagged Canonical Notch Ligand 2	1.99
SPRED1	Sprouty Related EVH1 Domain Containing 1	1.99
ZIC2	Zic Family Member 2	1.99
WWC1	WW And C2 Domain Containing 1	1.99
LMO2	LIM Domain Only 2	1.99
BBS2	Bardet-Biedl Syndrome 2	1.99
BBS4	Bardet-Biedl Syndrome 4	1.99
GDF15	Growth Differentiation Factor 15	1.99
GCG	Glucagon	1.99
GCM2	Glial Cells Missing Transcription Factor 2	1.99
CCL7	C-C Motif Chemokine Ligand 7	1.99
CCL19	C-C Motif Chemokine Ligand 19	1.99
CCT3	Chaperonin Containing TCP1 Subunit 3	1.99
GNA15	G Protein Subunit Alpha 15	1.99
LTK	Leukocyte Receptor Tyrosine Kinase	1.99
ATRIP	ATR Interacting Protein	1.99
LRBA	LPS Responsive Beige-Like Anchor Protein	1.99
F2RL2	Coagulation Factor II Thrombin Receptor Like 2	1.99
APCS	Amyloid P Component, Serum	1.99
AP2S1	Adaptor Related Protein Complex 2 Subunit Sigma 1	1.99
LPA	Lipoprotein(A)	1.99
FBN2	Fibrillin 2	1.99
AIM2	Absent In Melanoma 2	1.99
AIMP2	Aminoacyl TRNA Synthetase Complex Interacting Multifunctional Protein 2	1.99
BMP15	Bone Morphogenetic Protein 15	1.99
BCAP31	B Cell Receptor Associated Protein 31	1.99
MFNG	MFNG O-Fucosylpeptide 3-Beta-N-Acetylglucosaminyltransferase	1.99
CRYBB3	Crystallin Beta B3	1.99

EPX	Eosinophil Peroxidase	1.99
CEP57	Centrosomal Protein 57	1.99
CELF2	CUGBP Elav-Like Family Member 2	1.99
HNRNPC	Heterogeneous Nuclear Ribonucleoprotein C	1.99
RHBDF2	Rhomboid 5 Homolog 2	1.99
CNBP	CCHC-Type Zinc Finger Nucleic Acid Binding Protein	1.99
CLTB	Clathrin Light Chain B	1.99
CLDN16	Claudin 16	1.99
RFX5	Regulatory Factor X5	1.99
MLC1	Modulator Of VRAC Current 1	1.99
CFHR1	Complement Factor H Related 1	1.99
MS4A2	Membrane Spanning 4-Domains A2	1.99
DLG3	Discs Large MAGUK Scaffold Protein 3	1.99
DDB1	Damage Specific DNA Binding Protein 1	1.99
MPZL1	Myelin Protein Zero Like 1	1.99
MR1	Major Histocompatibility Complex, Class I-Related	1.99
MRE11	MRE11 Homolog, Double Strand Break Repair Nuclease	1.99
RNF8	Ring Finger Protein 8	1.99
RPL34	Ribosomal Protein L34	1.99
CREM	CAMP Responsive Element Modulator	1.99
HLA-DMB	Major Histocompatibility Complex, Class II, DM Beta	1.99
IL17RB	Interleukin 17 Receptor B	1.99
PER1	Period Circadian Regulator 1	1.99
PRPF6	Pre-MRNA Processing Factor 6	1.99
IL1RL1	Interleukin 1 Receptor Like 1	1.99
IL1RL2	Interleukin 1 Receptor Like 2	1.99
SETX	Senataxin	1.99
PAPPA	Pappalysin 1	1.99
IARS2	Isoleucyl-TRNA Synthetase 2, Mitochondrial	1.99
ICAM3	Intercellular Adhesion Molecule 3	1.99
PPIG	Peptidylprolyl Isomerase G	1.99
PANK1	Pantothenate Kinase 1	1.99
PCNT	Pericentrin	1.99
SNRNP200	Small Nuclear Ribonucleoprotein U5 Subunit 200	1.99
NFKBIB	NFKB Inhibitor Beta	1.99
RPLP0	Ribosomal Protein Lateral Stalk Subunit P0	1.99
RPL7	Ribosomal Protein L7	1.99
POLR2L	RNA Polymerase II, I And III Subunit L	1.99
PLOD1	Procollagen-Lysine,2-Oxoglutarate 5-Dioxygenase 1	1.99
SLC4A5	Solute Carrier Family 4 Member 5	1.99
SLAMF7	SLAM Family Member 7	1.99

RPS3A	Ribosomal Protein S3A	1.99
SRM	Spermidine Synthase	1.99
TOPBP1	DNA Topoisomerase II Binding Protein 1	1.99
LGALS8	Galectin 8	1.99
TREM1	Triggering Receptor Expressed On Myeloid Cells 1	1.99
PUS1	Pseudouridine Synthase 1	1.99
JPH3	Junctophilin 3	1.99
RASGRP3	RAS Guanyl Releasing Protein 3	1.99
KLRD1	Killer Cell Lectin Like Receptor D1	1.99
REV1	REV1 DNA Directed Polymerase	1.99
RAD18	RAD18 E3 Ubiquitin Protein Ligase	1.99
PCSK5	Proprotein Convertase Subtilisin/Kexin Type 5	1.99
HUS1	HUS1 Checkpoint Clamp Component	1.99
PRPF8	Pre-MRNA Processing Factor 8	1.99
LDB3	LIM Domain Binding 3	1.99
MUC5B	Mucin 5B, Oligomeric Mucus/Gel-Forming	1.99
RPA3	Replication Protein A3	1.99
CLDN2	Claudin 2	1.99
RPS29	Ribosomal Protein S29	1.99
CUL4A	Cullin 4A	1.99
DLL3	Delta Like Canonical Notch Ligand 3	1.99
POLK	DNA Polymerase Kappa	1.99
ID3	Inhibitor Of DNA Binding 3, HLH Protein	1.99
KIF3A	Kinesin Family Member 3A	1.99
CARD10	Caspase Recruitment Domain Family Member 10	1.99
CADM3	Cell Adhesion Molecule 3	1.99
CALB2	Calbindin 2	1.99
GOLGA2	Golgin A2	1.99
GORASP1	Golgi Reassembly Stacking Protein 1	1.99
GORASP2	Golgi Reassembly Stacking Protein 2	1.99
CCR9	C-C Motif Chemokine Receptor 9	1.99
GP5	Glycoprotein V Platelet	1.99
BACH2	BTB Domain And CNC Homolog 2	1.99
BBS10	Bardet-Biedl Syndrome 10	1.99
ARFGAP3	ADP Ribosylation Factor GTPase Activating Protein 3	1.99
FCGRT	Fc Fragment Of IgG Receptor And Transporter	1.99
LSR	Lipolysis Stimulated Lipoprotein Receptor	1.99
LRRC4C	Leucine Rich Repeat Containing 4C	1.99
MAVS	Mitochondrial Antiviral Signaling Protein	1.99
MAZ	MYC Associated Zinc Finger Protein	1.99
MBD1	Methyl-CpG Binding Domain Protein 1	1.99

ACSBG1	Acyl-CoA Synthetase Bubblegum Family Member 1	1.99
BSND	Barttin CLCNK Type Accessory Subunit Beta	1.99
FLT3LG	Fms Related Receptor Tyrosine Kinase 3 Ligand	1.99
CXCL16	C-X-C Motif Chemokine Ligand 16	1.99
CXCL5	C-X-C Motif Chemokine Ligand 5	1.99
RNASE3	Ribonuclease A Family Member 3	1.99
CRYBA1	Crystallin Beta A1	1.99
CRYBB2	Crystallin Beta B2	1.99
CD68	CD68 Molecule	1.99
CEP164	Centrosomal Protein 164	1.99
RHCE	Rh Blood Group CcEe Antigens	1.99
CLPS	Colipase	1.99
CLSPN	Claspin	1.99
COIL	Coilin	1.99
CFHR5	Complement Factor H Related 5	1.99
CIAO1	Cytosolic Iron-Sulfur Assembly Component 1	1.99
DEPDC5	DEP Domain Containing 5, GATOR1 Subcomplex Subunit	1.99
DTX1	Deltex E3 Ubiquitin Ligase 1	1.99
RPGR	Retinitis Pigmentosa GTPase Regulator	1.99
NALCN	Sodium Leak Channel, Non-Selective	1.99
EEF1E1	Eukaryotic Translation Elongation Factor 1 Epsilon 1	1.99
GPR83	G Protein-Coupled Receptor 83	1.99
CD276	CD276 Molecule	1.99
GAST	Gastrin	1.99
COL27A1	Collagen Type XXVII Alpha 1 Chain	1.99
CXCR6	C-X-C Motif Chemokine Receptor 6	1.99
NEO1	Neogenin 1	1.99
HPR	Haptoglobin-Related Protein	1.99
CD72	CD72 Molecule	1.99
HLA-DMA	Major Histocompatibility Complex, Class II, DM Alpha	1.99
HLA-DOA	Major Histocompatibility Complex, Class II, DO Alpha	1.99
HLA-DOB	Major Histocompatibility Complex, Class II, DO Beta	1.99
PEX11B	Peroxisomal Biogenesis Factor 11 Beta	1.99
SIM1	SIM BHLH Transcription Factor 1	1.99
SERINC1	Serine Incorporator 1	1.99
SH3BP5	SH3 Domain Binding Protein 5	1.99
PPL	Periplakin	1.99
PRG2	Proteoglycan 2, Pro Eosinophil Major Basic Protein	1.99
SCGB1A1	Secretoglobin Family 1A Member 1	1.99
RS1	Retinoschisin 1	1.99
NFYB	Nuclear Transcription Factor Y Subunit Beta	1.99

NME8	NME/NM23 Family Member 8	1.99
SMCHD1	Structural Maintenance Of Chromosomes Flexible Hinge Domain Containing 1	1.99
PLEK	Pleckstrin	1.99
SLAMF1	Signaling Lymphocytic Activation Molecule Family Member 1	1.99
SENP3	SUMO Specific Peptidase 3	1.99
SRP72	Signal Recognition Particle 72	1.99
SSRP1	Structure Specific Recognition Protein 1	1.99
TMSB4X	Thymosin Beta 4 X-Linked	1.99
TNFSF12	TNF Superfamily Member 12	1.99
TCTN3	Tectonic Family Member 3	1.99
PUS7	Pseudouridine Synthase 7	1.99
KLRC1	Killer Cell Lectin Like Receptor C1	1.99
TBPL1	TATA-Box Binding Protein Like 1	1.99
STX5	Syntaxin 5	1.99
RAD9A	RAD9 Checkpoint Clamp Component A	1.99
HLA-DRB5	Major Histocompatibility Complex, Class II, DR Beta 5	1.99
S100A7	S100 Calcium Binding Protein A7	1.99
POLD2	DNA Polymerase Delta 2, Accessory Subunit	1.99
POLD3	DNA Polymerase Delta 3, Accessory Subunit	1.99
POLQ	DNA Polymerase Theta	1.99
IAPP	Islet Amyloid Polypeptide	1.99
IFT88	Intraflagellar Transport 88	1.99
TFCP2	Transcription Factor CP2	1.99
UNC13A	Unc-13 Homolog A	1.99
BMPER	BMP Binding Endothelial Regulator	1.99
BBS5	Bardet-Biedl Syndrome 5	1.99
CCN2	Cellular Communication Network Factor 2	1.99
GJC3	Gap Junction Protein Gamma 3	1.99
CC2D2A	Coiled-Coil And C2 Domain Containing 2A	1.99
GOSR1	Golgi SNAP Receptor Complex Member 1	1.99
EXOSC10	Exosome Component 10	1.99
ARMC9	Armadillo Repeat Containing 9	1.99
ARHGAP32	Rho GTPase Activating Protein 32	1.99
MAGEA4	MAGE Family Member A4	1.99
ANKRD26	Ankyrin Repeat Domain 26	1.99
AGO2	Argonaute RISC Catalytic Component 2	1.99
MATN1	Matrilin 1	1.99
A2ML1	Alpha-2-Macroglobulin Like 1	1.99
AANAT	Aralkylamine N-Acetyltransferase	1.99
AP1S1	Adaptor Related Protein Complex 1 Subunit Sigma 1	1.99

HPX	Hemopexin	1.99
CRLF2	Cytokine Receptor Like Factor 2	1.99
BTLA	B And T Lymphocyte Associated	1.99
GTF2H3	General Transcription Factor IIH Subunit 3	1.99
CELSR3	Cadherin EGF LAG Seven-Pass G-Type Receptor 3	1.99
RHOD	Ras Homolog Family Member D	1.99
HLTF	Helicase Like Transcription Factor	1.99
HNRNPDL	Heterogeneous Nuclear Ribonucleoprotein D Like	1.99
HBS1L	HBS1 Like Translational GTPase	1.99
MLLT1	MLLT1 Super Elongation Complex Subunit	1.99
DERA	Deoxyribose-Phosphate Aldolase	1.99
DHRS2	Dehydrogenase/Reductase 2	1.99
MYOM2	Myomesin 2	1.99
CD83	CD83 Molecule	1.99
DEFB1	Defensin Beta 1	1.99
NDRG4	NDRG Family Member 4	1.99
NEIL1	Nei Like DNA Glycosylase 1	1.99
ELP3	Elongator Acetyltransferase Complex Subunit 3	1.99
CRELD1	Cysteine Rich With EGF Like Domains 1	1.99
SPG11	SPG11 Vesicle Trafficking Associated, Spatacsin	1.99
IL17RE	Interleukin 17 Receptor E	1.99
IL18RAP	Interleukin 18 Receptor Accessory Protein	1.99
SPN	Sialophorin	1.99
SERPINA7	Serpin Family A Member 7	1.99
SIX5	SIX Homeobox 5	1.99
NPHP3	Nephrocystin 3	1.99
PPP1R3C	Protein Phosphatase 1 Regulatory Subunit 3C	1.99
PPIP5K2	Diphosphoinositol Pentakisphosphate Kinase 2	1.99
SAA4	Serum Amyloid A4, Constitutive	1.99
SCARA5	Scavenger Receptor Class A Member 5	1.99
NFRKB	Nuclear Factor Related To KappaB Binding Protein	1.99
ENAM	Enamelin	1.99
NLRC5	NLR Family CARD Domain Containing 5	1.99
NIT2	Nitrilase Family Member 2	1.99
PKN3	Protein Kinase N3	1.99
SLC25A37	Solute Carrier Family 25 Member 37	1.99
SLC38A3	Solute Carrier Family 38 Member 3	1.99
NT5C1A	5'-Nucleotidase, Cytosolic IA	1.99
RPS4X	Ribosomal Protein S4 X-Linked	1.99
SHOX2	Short Stature Homeobox 2	1.99
SHPRH	SNF2 Histone Linker PHD RING Helicase	1.99

PHIP	Pleckstrin Homology Domain Interacting Protein	1.99
TNFRSF19	TNF Receptor Superfamily Member 19	1.99
UBASH3A	Ubiquitin Associated And SH3 Domain Containing A	1.99
LILRA2	Leukocyte Immunoglobulin Like Receptor A2	1.99
UBR2	Ubiquitin Protein Ligase E3 Component N-Recognin 2	1.99
KCNN1	Potassium Calcium-Activated Channel Subfamily N Member 1	1.99
TEX14	Testis Expressed 14, Intercellular Bridge Forming Factor	1.99
TECTA	Tectorin Alpha	1.99
DLX2	Distal-Less Homeobox 2	1.99
DLX6	Distal-Less Homeobox 6	1.99
POLM	DNA Polymerase Mu	1.99
TGM6	Transglutaminase 6	1.99
KRT9	Keratin 9	1.99
YBX2	Y-Box Binding Protein 2	1.99
BBS7	Bardet-Biedl Syndrome 7	1.99
CCL1	C-C Motif Chemokine Ligand 1	1.99
CCL13	C-C Motif Chemokine Ligand 13	1.99
ATCAY	ATCAY Kinesin Light Chain Interacting Caytaxin	1.99
BBS1	Bardet-Biedl Syndrome 1	1.99
FIP1L1	Factor Interacting With PAPOLA And CPSF1	1.99
EXPH5	Exophilin 5	1.99
LRRC7	Leucine Rich Repeat Containing 7	1.99
GTPBP1	GTP Binding Protein 1	1.99
CEBPD	CCAAT Enhancer Binding Protein Delta	1.99
HEY2	Hes Related Family BHLH Transcription Factor With YRPW Motif 2	1.99
RGS1	Regulator Of G Protein Signaling 1	1.99
DYNC2H1	Dynein Cytoplasmic 2 Heavy Chain 1	1.99
IL25	Interleukin 25	1.99
NOP58	NOP58 Ribonucleoprotein	1.99
NPNT	Nephronectin	1.99
NLRP6	NLR Family Pyrin Domain Containing 6	1.99
SLC25A28	Solute Carrier Family 25 Member 28	1.99
SHOX	Short Stature Homeobox	1.99
NPRL3	NPR3 Like, GATOR1 Complex Subunit	1.99
PTGFRN	Prostaglandin F2 Receptor Inhibitor	1.99
TOR1B	Torsin Family 1 Member B	1.99
TSLP	Thymic Stromal Lymphopoietin	1.99
ST13	ST13 Hsp70 Interacting Protein	1.99
IRGM	Immunity Related GTPase M	1.99
RAB34	RAB34, Member RAS Oncogene Family	1.99

KARS1	Lysyl-TRNA Synthetase 1	1.99
PSMD5	Proteasome 26S Subunit, Non-ATPase 5	1.99
OTUB2	OTU Deubiquitinase, Ubiquitin Aldehyde Binding 2	1.99
SULT1A3	Sulfotransferase Family 1A Member 3	1.99
STK40	Serine/Threonine Kinase 40	1.99
WDR48	WD Repeat Domain 48	1.99
ZNF23	Zinc Finger Protein 23	1.99
XAB2	XPA Binding Protein 2	1.99
C1QTNF6	C1q And TNF Related 6	1.99
CCL26	C-C Motif Chemokine Ligand 26	1.99
GOLGB1	Golgin B1	1.99
FGGY	FGGY Carbohydrate Kinase Domain Containing	1.99
ARFRP1	ADP Ribosylation Factor Related Protein 1	1.99
MAGEA1	MAGE Family Member A1	1.99
MAGEA11	MAGE Family Member A11	1.99
LY86	Lymphocyte Antigen 86	1.99
ALYREF	Aly/REF Export Factor	1.99
AJUBA	Ajuba LIM Protein	1.99
MED28	Mediator Complex Subunit 28	1.99
MBNL2	Muscleblind Like Splicing Regulator 2	1.99
CLEC4E	C-Type Lectin Domain Family 4 Member E	1.99
HEPHL1	Hephaestin Like 1	1.99
CELA3B	Chymotrypsin Like Elastase 3B	1.99
CELF1	CUGBP Elav-Like Family Member 1	1.99
CENPB	Centromere Protein B	1.99
CDR2	Cerebellar Degeneration Related Protein 2	1.99
HES7	Hes Family BHLH Transcription Factor 7	1.99
CLPTM1	CLPTM1 Regulator Of GABA Type A Receptor Forward Trafficking	1.99
RFXAP	Regulatory Factor X Associated Protein	1.99
CFHR2	Complement Factor H Related 2	1.99
DPYSL5	Dihydropyrimidinase Like 5	1.99
GPN1	GPN-Loop GTPase 1	1.99
MRGPRX2	MAS Related GPR Family Member X2	1.99
RNF111	Ring Finger Protein 111	1.99
SPIN1	Spindlin 1	1.99
PEX11A	Peroxisomal Biogenesis Factor 11 Alpha	1.99
PRPF19	Pre-MRNA Processing Factor 19	1.99
SERINC3	Serine Incorporator 3	1.99
PPP1R3B	Protein Phosphatase 1 Regulatory Subunit 3B	1.99
IFT80	Intraflagellar Transport 80	1.99

SNRPB2	Small Nuclear Ribonucleoprotein Polypeptide B2	1.99
NLRP5	NLR Family Pyrin Domain Containing 5	1.99
PKP3	Plakophilin 3	1.99
OSBPL3	Oxysterol Binding Protein Like 3	1.99
POC1A	POC1 Centriolar Protein A	1.99
PLRG1	Pleiotropic Regulator 1	1.99
SENP6	SUMO Specific Peptidase 6	1.99
IL9R	Interleukin 9 Receptor	1.99
PTER	Phosphotriesterase Related	1.99
PIEZO1	Piezo Type Mechanosensitive Ion Channel Component 1	1.99
SRRT	Serrate, RNA Effector Molecule	1.99
TRIM68	Tripartite Motif Containing 68	1.99
TXNDC15	Thioredoxin Domain Containing 15	1.99
TAGAP	T Cell Activation RhoGTPase Activating Protein	1.99
KLRC2	Killer Cell Lectin Like Receptor C2	1.99
TMEM231	Transmembrane Protein 231	1.99
DYNC1LI1	Dynein Cytoplasmic 1 Light Intermediate Chain 1	1.99
DTL	Denticleless E3 Ubiquitin Protein Ligase Homolog	1.99
SAMM50	SAMM50 Sorting And Assembly Machinery Component	1.99
POLD4	DNA Polymerase Delta 4, Accessory Subunit	1.99
RAB3IP	RAB3A Interacting Protein	1.99
PACRG	Parkin Coregulated	1.99
IL4I1	Interleukin 4 Induced 1	1.99
TGOLN2	Trans-Golgi Network Protein 2	1.99
KRT6C	Keratin 6C	1.99
WDR72	WD Repeat Domain 72	1.99
GAR1	GAR1 Ribonucleoprotein	1.99
CCP110	Centriolar Coiled-Coil Protein 110	1.99
AQR	Aquarius Intron-Binding Spliceosomal Factor	1.99
LUC7L2	LUC7 Like 2, Pre-MRNA Splicing Factor	1.99
MAGEA3	MAGE Family Member A3	1.99
LRRC2	Leucine Rich Repeat Containing 2	1.99
ANKMY1	Ankyrin Repeat And MYND Domain Containing 1	1.99
EPRS1	Glutamyl-Prolyl-TRNA Synthetase 1	1.99
ERH	ERH MRNA Splicing And Mitosis Factor	1.99
AGBL1	ATP/GTP Binding Protein Like 1	1.99
MBNL3	Muscleblind Like Splicing Regulator 3	1.99
AARS1	Alanyl-TRNA Synthetase 1	1.99
CLEC6A	C-Type Lectin Domain Containing 6A	1.99
FOXJ1	Forkhead Box J1	1.99
GPR174	G Protein-Coupled Receptor 174	1.99

CELA1	Chymotrypsin Like Elastase 1	1.99
CLPTM1L	CLPTM1 Like	1.99
CLDN15	Claudin 15	1.99
MMEL1	Membrane Metalloendopeptidase Like 1	1.99
MIXL1	Mix Paired-Like Homeobox	1.99
HBQ1	Hemoglobin Subunit Theta 1	1.99
CEP89	Centrosomal Protein 89	1.99
MMS19	MMS19 Homolog, Cytosolic Iron-Sulfur Assembly Component	1.99
DMTN	Dematin Actin Binding Protein	1.99
MTUS1	Microtubule Associated Scaffold Protein 1	1.99
MORC3	MORC Family CW-Type Zinc Finger 3	1.99
RNF207	Ring Finger Protein 207	1.99
HARS1	Histidyl-TRNA Synthetase 1	1.99
CST2	Cystatin SA	1.99
NDUFAF5	NADH:Ubiquinone Oxidoreductase Complex Assembly Factor 5	1.99
NEK10	NIMA Related Kinase 10	1.99
IL17C	Interleukin 17C	1.99
SERINC2	Serine Incorporator 2	1.99
PAMR1	Peptidase Domain Containing Associated With Muscle Regeneration 1	1.99
SNX19	Sorting Nexin 19	1.99
PRICKLE4	Prickle Planar Cell Polarity Protein 4	1.99
SAA2	Serum Amyloid A2	1.99
SCCPDH	Saccharopine Dehydrogenase (Putative)	1.99
HSPBP1	HSPA (Hsp70) Binding Protein 1	1.99
PMCH	Pro-Melanin Concentrating Hormone	1.99
OGFOD1	2-Oxoglutarate And Iron Dependent Oxygenase Domain Containing 1	1.99
SENP7	SUMO Specific Peptidase 7	1.99
PHF11	PHD Finger Protein 11	1.99
LARS1	Leucyl-TRNA Synthetase 1	1.99
TRIM35	Tripartite Motif Containing 35	1.99
LECT2	Leukocyte Cell Derived Chemotaxin 2	1.99
KNTC1	Kinetochore Associated 1	1.99
KIR3DL3	Killer Cell Immunoglobulin Like Receptor, Three Ig Domains And Long Cytoplasmic Tail 3	1.99
TMEM216	Transmembrane Protein 216	1.99
TMEM138	Transmembrane Protein 138	1.99
E4F1	E4F Transcription Factor 1	1.99
NECTIN2	Nectin Cell Adhesion Molecule 2	1.99
POMGNT2	Protein O-Linked Mannose N-Acetylglucosaminyltransferase 2 (Beta 1,4-)	1.99

POPDC2	Popeye Domain Containing 2	1.99
SYCP1	Synaptonemal Complex Protein 1	1.99
TIMD4	T Cell Immunoglobulin And Mucin Domain Containing 4	1.99
WBP11	WW Domain Binding Protein 11	1.99
YARS1	Tyrosyl-TRNA Synthetase 1	1.99
UBR3	Ubiquitin Protein Ligase E3 Component N-Recognin 3	1.99
BBS12	Bardet-Biedl Syndrome 12	1.99
CABP7	Calcium Binding Protein 7	1.99
MARS1	Methionyl-TRNA Synthetase 1	1.99
EVPL	Envoplakin	1.99
EXOC6B	Exocyst Complex Component 6B	1.99
FAM107B	Family With Sequence Similarity 107 Member B	1.99
MAGEA10	MAGE Family Member A10	1.99
MAGEA6	MAGE Family Member A6	1.99
ALKBH1	AlkB Homolog 1, Histone H2A Dioxygenase	1.99
MESP2	Mesoderm Posterior BHLH Transcription Factor 2	1.99
CROCC	Ciliary Rootlet Coiled-Coil, Rootletin	1.99
GXYLT1	Glucoside Xylosyltransferase 1	1.99
COMMD6	COMM Domain Containing 6	1.99
CFHR4	Complement Factor H Related 4	1.99
DNAJC21	DnaJ Heat Shock Protein Family (Hsp40) Member C21	1.99
MRPL38	Mitochondrial Ribosomal Protein L38	1.99
DMWD	DM1 Locus, WD Repeat Containing	1.99
EFHC2	EF-Hand Domain Containing 2	1.99
GPKOW	G-Patch Domain And KOW Motifs	1.99
MOSPD1	Motile Sperm Domain Containing 1	1.99
MOB3B	MOB Kinase Activator 3B	1.99
DCLRE1A	DNA Cross-Link Repair 1A	1.99
CPNE2	Copine 2	1.99
SCT	Secretin	1.99
SCHIP1	Schwannomin Interacting Protein 1	1.99
SARS1	Seryl-TRNA Synthetase 1	1.99
NUP188	Nucleoporin 188	1.99
SLC22A15	Solute Carrier Family 22 Member 15	1.99
PHRF1	PHD And Ring Finger Domains 1	1.99
LACC1	Laccase Domain Containing 1	1.99
UBR7	Ubiquitin Protein Ligase E3 Component N-Recognin 7	1.99
TRIM40	Tripartite Motif Containing 40	1.99
TXNDC2	Thioredoxin Domain Containing 2	1.99
PTPA	Protein Phosphatase 2 Phosphatase Activator	1.99
PUS10	Pseudouridine Synthase 10	1.99

KCNRG	Potassium Channel Regulator	1.99
KIF24	Kinesin Family Member 24	1.99
TMEM79	Transmembrane Protein 79	1.99
CENPI	Centromere Protein I	1.99
WDFY4	WDFY Family Member 4	1.99
ZRSR2	Zinc Finger CCCH-Type, RNA Binding Motif And Serine/Arginine Rich 2	1.99
MAGEA8	MAGE Family Member A8	1.99
AFDN	Afadin, Adherens Junction Formation Factor	1.99
RMND5B	Required For Meiotic Nuclear Division 5 Homolog B	1.99
CBLIF	Cobalamin Binding Intrinsic Factor	1.99
CEP97	Centrosomal Protein 97	1.99
H3-3A	H3.3 Histone A	1.99
MTUS2	Microtubule Associated Scaffold Protein 2	1.99
RRS1	Ribosome Biogenesis Regulator 1 Homolog	1.99
SYAP1	Synapse Associated Protein 1	1.99
KIF19	Kinesin Family Member 19	1.99
NOL7	Nucleolar Protein 7	1.99
POLN	DNA Polymerase Nu	1.99
KRT73	Keratin 73	1.99
WDTC1	WD And Tetratricopeptide Repeats 1	1.99
ZFP3	ZFP3 Zinc Finger Protein	1.99
TRIM47	Tripartite Motif Containing 47	1.99
WIZ	WIZ Zinc Finger	1.99
C3orf52	Chromosome 3 Open Reading Frame 52	1.99
ATAD5	ATPase Family AAA Domain Containing 5	1.99
FBXL15	F-Box And Leucine Rich Repeat Protein 15	1.99
ANKRD55	Ankyrin Repeat Domain 55	1.99
METTL4	Methyltransferase Like 4	1.99
BPIFB1	BPI Fold Containing Family B Member 1	1.99
MELTF	Melanotransferrin	1.99
CTAGE1	Cutaneous T Cell Lymphoma-Associated Antigen 1	1.99
CDX4	Caudal Type Homeobox 4	1.99
HAUS3	HAUS Augmin Like Complex Subunit 3	1.99
H3-3B	H3.3 Histone B	1.99
H4-16	H4 Histone 16	1.99
EIF1AD	Eukaryotic Translation Initiation Factor 1A Domain Containing	1.99
NECTIN3	Nectin Cell Adhesion Molecule 3	1.99
EME2	Essential Meiotic Structure-Specific Endonuclease Subunit 2	1.99
SKOR1	SKI Family Transcriptional Corepressor 1	1.99
SNRNP48	Small Nuclear Ribonucleoprotein U11/U12 Subunit 48	1.99

ENDOU	Endonuclease, Poly(U) Specific	1.99
SLX1B	SLX1 Homolog B, Structure-Specific Endonuclease Subunit	1.99
POC5	POC5 Centriolar Protein	1.99
RPS4Y1	Ribosomal Protein S4 Y-Linked 1	1.99
TRIM42	Tripartite Motif Containing 42	1.99
TRIM60	Tripartite Motif Containing 60	1.99
TRUB2	TruB Pseudouridine Synthase Family Member 2	1.99
TSPY1	Testis Specific Protein Y-Linked 1	1.99
KDF1	Keratinocyte Differentiation Factor 1	1.99
KAZN	Kazrin, Periplakin Interacting Protein	1.99
TMIE	Transmembrane Inner Ear	1.99
PRIMPOL	Primase And DNA Directed Polymerase	1.99
NAF1	Nuclear Assembly Factor 1 Ribonucleoprotein	1.99
SCLT1	Sodium Channel And Clathrin Linker 1	1.99
NOL10	Nucleolar Protein 10	1.99
OTOG	Otogelin	1.99
PSMB11	Proteasome Subunit Beta 11	1.99
XXYLT1	Xyloside Xylosyltransferase 1	1.99
CARS1	Cysteinyl-TRNA Synthetase 1	1.99
ESX1	ESX Homeobox 1	1.99
MAGEA9	MAGE Family Member A9	1.99
CLEC4C	C-Type Lectin Domain Family 4 Member C	1.99
DMBX1	Diencephalon/Mesencephalon Homeobox 1	1.99
CD300LD	CD300 Molecule Like Family Member D	1.99
CSH2	Chorionic Somatomammotropin Hormone 2	1.99
SFTA3	Surfactant Associated 3	1.99
IGHG1	Immunoglobulin Heavy Constant Gamma 1 (G1m Marker)	1.99
ENDOV	Endonuclease V	1.99
OAF	Out At First Homolog	1.99
NPS	Neuropeptide S	1.99
NUDT18	Nudix Hydrolase 18	1.99
SPTSSB	Serine Palmitoyltransferase Small Subunit B	1.99
L3MBTL4	L3MBTL Histone Methyl-Lysine Binding Protein 4	1.99
TXNDC16	Thioredoxin Domain Containing 16	1.99
PRIMA1	Proline Rich Membrane Anchor 1	1.99
N4BP3	NEDD4 Binding Protein 3	1.99
VSIR	V-Set Immunoregulatory Receptor	1.99
CCDC122	Coiled-Coil Domain Containing 122	1.99
FABP12	Fatty Acid Binding Protein 12	1.99
MAGEA2	MAGE Family Member A2	1.99
MT-CYB	Mitochondrially Encoded Cytochrome B	1.99

MYZAP	Myocardial Zonula Adherens Protein	1.99
GPR148	G Protein-Coupled Receptor 148	1.99
IGHM	Immunoglobulin Heavy Constant Mu	1.99
RSPH6A	Radial Spoke Head 6 Homolog A	1.99
RTP1	Receptor Transporter Protein 1	1.99
SMR3B	Submaxillary Gland Androgen Regulated Protein 3B	1.99
OR6C2	Olfactory Receptor Family 6 Subfamily C Member 2	1.99
TRIM34	Tripartite Motif Containing 34	1.99
TVP23A	Trans-Golgi Network Vesicle Protein 23 Homolog A	1.99
TEPP	Testis, Prostate And Placenta Expressed	1.99
TECTB	Tectorin Beta	1.99
TMEM17	Transmembrane Protein 17	1.99
FAAP24	FA Core Complex Associated Protein 24	1.99
ARHGAP40	Rho GTPase Activating Protein 40	1.99
RNASE10	Ribonuclease A Family Member 10 (Inactive)	1.99
CDR1	Cerebellar Degeneration Related Protein 1	1.99
CGB7	Chorionic Gonadotropin Subunit Beta 7	1.99
CXorf21	Chromosome X Open Reading Frame 21	1.99
SLX1A	SLX1 Homolog A, Structure-Specific Endonuclease Subunit	1.99
JMJD8	Jumonji Domain Containing 8	1.99
TENT4A	Terminal Nucleotidyltransferase 4A	1.99
TEN1	TEN1 Subunit Of CST Complex	1.99
HLA-DRB4	Major Histocompatibility Complex, Class II, DR Beta 4	1.99
CACTIN	Cactin, Spliceosome C Complex Subunit	1.99
MCIDAS	Multiciliate Differentiation And DNA Synthesis Associated Cell Cycle Protein	1.99
CGAS	Cyclic GMP-AMP Synthase	1.99
H3C14	H3 Clustered Histone 14	1.99
CIAO2B	Cytosolic Iron-Sulfur Assembly Component 2B	1.99
GPR137C	G Protein-Coupled Receptor 137C	1.99
OR10H4	Olfactory Receptor Family 10 Subfamily H Member 4	1.99
TENT4B	Terminal Nucleotidyltransferase 4B	1.99
C10orf99	Chromosome 10 Open Reading Frame 99	1.99
MAGEA2B	MAGE Family Member A2B	1.99
CEP295	Centrosomal Protein 295	1.99
CPLANE1	Ciliogenesis And Planar Polarity Effector 1	1.99
KATNIP	Katanin Interacting Protein	1.99
DENND2B	DENN Domain Containing 2B	1.99
PIFO	Primary Cilia Formation	1.99
MAGEA5	MAGE Family Member A5	1.99
CGB8	Chorionic Gonadotropin Subunit Beta 8	1.99

PLPPR3	Phospholipid Phosphatase Related 3	1.99
ODAPH	Odontogenesis Associated Phosphoprotein	1.99
YIPF7	Yip1 Domain Family Member 7	1.99
ATXN8OS	ATXN8 Opposite Strand LncRNA	1.99
MUCL3	Mucin Like 3	1.99
SRARP	Steroid Receptor Associated And Regulated Protein	1.99
PEDS1	Plasmanylethanolamine Desaturase 1	1.99
RPEL1	Ribulose-5-Phosphate-3-Epimerase Like 1	1.99
MIR21	MicroRNA 21	1.99
MALAT1	Metastasis Associated Lung Adenocarcinoma Transcript 1	1.99
ERFE	Erythroferrone	1.99
MIR184	MicroRNA 184	1.99
NEAT1	Nuclear Paraspeckle Assembly Transcript 1	1.99
KRTAP19-2	Keratin Associated Protein 19-2	1.99
MIR146A	MicroRNA 146a	1.99
GNAS-AS1	GNAS Antisense RNA 1	1.99
MIR27A	MicroRNA 27a	1.99
MIR150	MicroRNA 150	1.99
MIR10A	MicroRNA 10a	1.99
MIR185	MicroRNA 185	1.99
MIR192	MicroRNA 192	1.99
MIR210	MicroRNA 210	1.99
MIR99A	MicroRNA 99a	1.99
MIR499A	MicroRNA 499a	1.99
MIR433	MicroRNA 433	1.99
MIR23A	MicroRNA 23a	1.99
MIR320A	MicroRNA 320a	1.99
MEG8	Maternally Expressed 8, Small Nucleolar RNA Host Gene	1.99
MIR30A	MicroRNA 30a	1.99
MIR615	MicroRNA 615	1.99
MIRLET7E	MicroRNA Let-7e	1.99
EGOT	Eosinophil Granule Ontogeny Transcript	1.99
MIR195	MicroRNA 195	1.99
MIR345	MicroRNA 345	1.99
MIR197	MicroRNA 197	1.99
MIR148A	MicroRNA 148a	1.99
MIR15B	MicroRNA 15b	1.99
MIR134	MicroRNA 134	1.99
MIR346	MicroRNA 346	1.99
MIR208A	MicroRNA 208a	1.99
MIR423	MicroRNA 423	1.99

MIR324	MicroRNA 324	1.99
C11orf98	Chromosome 11 Open Reading Frame 98	1.99
MIR33A	MicroRNA 33a	1.99
MIR484	MicroRNA 484	1.99
RNU6-1	RNA, U6 Small Nuclear 1	1.99
SCARNA8	Small Cajal Body-Specific RNA 8	1.99
MIR130B	MicroRNA 130b	1.99
MIR296	MicroRNA 296	1.99
MIR654	MicroRNA 654	1.99
MIR675	MicroRNA 675	1.99
MIR612	MicroRNA 612	1.99
MIR638	MicroRNA 638	1.99
MIR381	MicroRNA 381	1.99
MIR494	MicroRNA 494	1.99
SNORD63	Small Nucleolar RNA, C/D Box 63	1.99
RTEL1-TNFRSF6B	RTEL1-TNFRSF6B Readthrough (NMD Candidate)	1.99
TRA	T Cell Receptor Alpha Locus	1.99
MIR608	MicroRNA 608	1.99
SNORA56	Small Nucleolar RNA, H/ACA Box 56	1.99
MIR1304	MicroRNA 1304	1.99
FLG-AS1	FLG Antisense RNA 1	1.99
MIR198	MicroRNA 198	1.99
MIR518B	MicroRNA 518b	1.99
MIR557	MicroRNA 557	1.99
MIR575	MicroRNA 575	1.99
MIR601	MicroRNA 601	1.99
MIR657	MicroRNA 657	1.99
MIR637	MicroRNA 637	1.99
MIR642A	MicroRNA 642a	1.99
MIR92B	MicroRNA 92b	1.99
MIR769	MicroRNA 769	1.99
DLGAP1-AS1	DLGAP1 Antisense RNA 1	1.99
DLGAP1-AS2	DLGAP1 Antisense RNA 2	1.99
MIR486-1	MicroRNA 486-1	1.99
RNU5A-1	RNA, U5A Small Nuclear 1	1.99
RNU11	RNA, U11 Small Nuclear	1.99
SNORA15	Small Nucleolar RNA, H/ACA Box 15	1.99
SNORA24	Small Nucleolar RNA, H/ACA Box 24	1.99
SNORA67	Small Nucleolar RNA, H/ACA Box 67	1.99
SNORA49	Small Nucleolar RNA, H/ACA Box 49	1.99

MIR548AA1	MicroRNA 548aa-1	1.99
MIR583	MicroRNA 583	1.99
MIR662	MicroRNA 662	1.99
MIR663A	MicroRNA 663a	1.99
DUX4L7	Double Homeobox 4 Like 7 (Pseudogene)	1.99
MIR600	MicroRNA 600	1.99
MIR658	MicroRNA 658	1.99
MIR611	MicroRNA 611	1.99
SNORD28	Small Nucleolar RNA, C/D Box 28	1.99
PARTICL	Promoter Of MAT2A Antisense Radiation-Induced Circulating Long Non-Coding RNA	1.99
LINC00309	Long Intergenic Non-Protein Coding RNA 309	1.99
MIR4260	MicroRNA 4260	1.99
MIR325	MicroRNA 325	1.99
ALMS1P1	ALMS1 Pseudogene 1	1.99
MIR596	MicroRNA 596	1.99
MIR602	MicroRNA 602	1.99
MIR622	MicroRNA 622	1.99
LINC01193	Long Intergenic Non-Protein Coding RNA 1193	1.99
MIR663AHG	MIR663A Host Gene	1.99
MIR4673	MicroRNA 4673	1.99
TRG	T Cell Receptor Gamma Locus	1.99
LINC02551	Long Intergenic Non-Protein Coding RNA 2551	1.99
TRI-AAT4-1	TRNA-Ile (Anticodon AAT) 4-1	1.99
TRT-AGT1-2	TRNA-Thr (Anticodon AGT) 1-2	1.99
RNU12-2P	RNA, U12 Small Nuclear 2, Pseudogene	1.99
LOC105372273	Uncharacterized LOC105372273	1.99
TRS-AGA2-6	TRNA-Ser (Anticodon AGA) 2-6	1.99
ABCD1P4	ATP Binding Cassette Subfamily D Member 1 Pseudogene 4	1.99
ABCD1P2	ATP Binding Cassette Subfamily D Member 1 Pseudogene 2	1.99
ABCD1P3	ATP Binding Cassette Subfamily D Member 1 Pseudogene 3	1.99
ENSG00000207300		1.99
LOC108783645	HFE Antisense RNA	1.99
ENSG00000266919		1.99
ABCD1P1	ATP Binding Cassette Subfamily D Member 1 Pseudogene 1	1.99
IGES	Immunoglobulin E Concentration, Serum	1.99
PIRC2	Piwi-Interacting RNA Cluster 2	1.99
AGMX2	Agammaglobulinemia, X-Linked 2 (With Growth Hormone Deficiency)	1.99
EEC1	Ectrodactyly, Ectodermal Dysplasia And Cleft Lip/Palate Syndrome 1	1.99
SLEN3	Systemic Lupus Erythematosus With Nephritis 3	1.99

SLEH1	Systemic Lupus Erythematosus With Hemolytic Anemia 1	1.99
SLEN1	Systemic Lupus Erythematosus With Nephritis 1	1.99
SLEN2	Systemic Lupus Erythematosus With Nephritis 2	1.99
AOS	Adams-Oliver Syndrome	1.99
HYP10	Hypotrichosis 10	1.99
LOC113687175	Sharpr-MPRA Regulatory Region 4647	1.99
LOC108167311	Proximal ADA Alu-Mediated Recombination Region	1.99
LOC108167312	Distal ADA Alu-Mediated Recombination Region	1.99
LOC107303343	Adenosine Deaminase Intronic Regulatory Elements	1.99
THRB	Thyroid Hormone Receptor Beta	1.88
SOX9	SRY-Box Transcription Factor 9	1.75
RXRA	Retinoid X Receptor Alpha	1.73
PDPN	Podoplanin	1.73
RNASEH2CP1	Ribonuclease H2 Subunit C Pseudogene 1	1.73
NCOR1	Nuclear Receptor Corepressor 1	1.69
NR5A1	Nuclear Receptor Subfamily 5 Group A Member 1	1.64
NES	Nestin	1.64
UCN	Urocortin	1.64
ZDHHC13	Zinc Finger DHHC-Type Palmitoyltransferase 13	1.64
WT1	WT1 Transcription Factor	1.58
ADRA1A	Adrenoceptor Alpha 1A	1.58
BIRC5	Baculoviral IAP Repeat Containing 5	1.58
PTGS1	Prostaglandin-Endoperoxide Synthase 1	1.58
ICAM2	Intercellular Adhesion Molecule 2	1.58
TSHR	Thyroid Stimulating Hormone Receptor	1.5
NR4A1	Nuclear Receptor Subfamily 4 Group A Member 1	1.5
F8	Coagulation Factor VIII	1.5
TCF7L2	Transcription Factor 7 Like 2	1.5
ACP1	Acid Phosphatase 1	1.5
MED1	Mediator Complex Subunit 1	1.5
MCHR2	Melanin Concentrating Hormone Receptor 2	1.5
CXCL2	C-X-C Motif Chemokine Ligand 2	1.5
SPATA5	Spermatogenesis Associated 5	1.5
HSPA1B	Heat Shock Protein Family A (Hsp70) Member 1B	1.5
ZPR1	ZPR1 Zinc Finger	1.5
FCGR1B	Fc Fragment Of IgG Receptor Ib	1.5
TBXT	T-Box Transcription Factor T	1.5
MIR30B	MicroRNA 30b	1.5
BCL2	BCL2 Apoptosis Regulator	1.4
THADA	THADA Armadillo Repeat Containing	1.39
PTGES	Prostaglandin E Synthase	1.31

HSD11B1	Hydroxysteroid 11-Beta Dehydrogenase 1	1.21
FPR1	Formyl Peptide Receptor 1	1.21
FPR2	Formyl Peptide Receptor 2	1.21
LTBP1	Latent Transforming Growth Factor Beta Binding Protein 1	1.21
FPR3	Formyl Peptide Receptor 3	1.21
RUNX1	RUNX Family Transcription Factor 1	1.14
ANGPT1	Angiopoietin 1	1.14
DFFA	DNA Fragmentation Factor Subunit Alpha	1.14
COPB2	COPI Coat Complex Subunit Beta 2	1.14
KCNE1	Potassium Voltage-Gated Channel Subfamily E Regulatory Subunit 1	1.14
FAF1	Fas Associated Factor 1	1.14
DKK2	Dickkopf WNT Signaling Pathway Inhibitor 2	1.14
RUNX3	RUNX Family Transcription Factor 3	1.14
RSPO2	R-Spondin 2	1.14
PLCD4	Phospholipase C Delta 4	1.14
RCAN1	Regulator Of Calcineurin 1	1.14
TLE3	TLE Family Member 3, Transcriptional Corepressor	1.14
CDCA7	Cell Division Cycle Associated 7	1.14
PRRX1	Paired Related Homeobox 1	1.14
OPHN1	Oligophrenin 1	1.14
AADAC	Arylacetamide Deacetylase	1.14
MRPS22	Mitochondrial Ribosomal Protein S22	1.14
PLEKHM1	Pleckstrin Homology And RUN Domain Containing M1	1.14
TBX15	T-Box Transcription Factor 15	1.14
KLF15	Kruppel Like Factor 15	1.14
EBF1	EBF Transcription Factor 1	1.14
FAR2	Fatty Acyl-CoA Reductase 2	1.14
CHST15	Carbohydrate Sulfotransferase 15	1.14
RPLP1	Ribosomal Protein Lateral Stalk Subunit P1	1.14
SUCNR1	Succinate Receptor 1	1.14
EXOC2	Exocyst Complex Component 2	1.14
AKAP1	A-Kinase Anchoring Protein 1	1.14
BHLHE41	Basic Helix-Loop-Helix Family Member E41	1.14
CDK5R2	Cyclin Dependent Kinase 5 Regulatory Subunit 2	1.14
MTX2	Metaxin 2	1.14
CRTC3	CREB Regulated Transcription Coactivator 3	1.14
NR2C1	Nuclear Receptor Subfamily 2 Group C Member 1	1.14
LHPP	Phospholysine Phosphohistidine Inorganic Pyrophosphate Phosphatase	1.14
KANSL1	KAT8 Regulatory NSL Complex Subunit 1	1.14
USP37	Ubiquitin Specific Peptidase 37	1.14

MEMO1	Mediator Of Cell Motility 1	1.14
CLIC6	Chloride Intracellular Channel 6	1.14
EIF3E	Eukaryotic Translation Initiation Factor 3 Subunit E	1.14
OBSL1	Obscurin Like Cytoskeletal Adaptor 1	1.14
GORAB	Golgin, RAB6 Interacting	1.14
ASB1	Ankyrin Repeat And SOCS Box Containing 1	1.14
MKLN1	Muskelin 1	1.14
DPY30	Dpy-30 Histone Methyltransferase Complex Regulatory Subunit	1.14
KLF8	Kruppel Like Factor 8	1.14
SRRM1	Serine And Arginine Repetitive Matrix 1	1.14
CASZ1	Castor Zinc Finger 1	1.14
GINS2	GINS Complex Subunit 2	1.14
HOXD3	Homeobox D3	1.14
SERPINB12	Serpin Family B Member 12	1.14
ZRANB1	Zinc Finger RANBP2-Type Containing 1	1.14
DMRTA2	DMRT Like Family A2	1.14
ZNF142	Zinc Finger Protein 142	1.14
FAM53B	Family With Sequence Similarity 53 Member B	1.14
ANKZF1	Ankyrin Repeat And Zinc Finger Peptidyl TRNA Hydrolase 1	1.14
FOXD2	Forkhead Box D2	1.14
GSE1	Gse1 Coiled-Coil Protein	1.14
SPAG17	Sperm Associated Antigen 17	1.14
ZFAND2B	Zinc Finger AN1-Type Containing 2B	1.14
CENPW	Centromere Protein W	1.14
HUS1B	HUS1 Checkpoint Clamp Component B	1.14
SYF2	SYF2 Pre-MRNA Splicing Factor	1.14
EMC2	ER Membrane Protein Complex Subunit 2	1.14
OFCC1	Orofacial Cleft 1 Candidate 1	1.14
TMEM74	Transmembrane Protein 74	1.14
RCCD1	RCC1 Domain Containing 1	1.14
CNPPD1	Cyclin Pas1/PHO80 Domain Containing 1	1.14
ZXDA	Zinc Finger X-Linked Duplicated A	1.14
LRMDA	Leucine Rich Melanocyte Differentiation Associated	1.14
C12orf71	Chromosome 12 Open Reading Frame 71	1.14
RSRP1	Arginine And Serine Rich Protein 1	1.14
FAM104B	Family With Sequence Similarity 104 Member B	1.14
FOXL2NB	FOXL2 Neighbor	1.14
EEF1AKMT2	EEF1A Lysine Methyltransferase 2	1.14
PRR23C	Proline Rich 23C	1.14
CFAP299	Cilia And Flagella Associated Protein 299	1.14
PRR23A	Proline Rich 23A	1.14

HULC	Hepatocellular Carcinoma Up-Regulated Long Non-Coding RNA	1.14
HOXD-AS2	HOXD Cluster Antisense RNA 2	1.14
LINC00593	Long Intergenic Non-Protein Coding RNA 593	1.14
DRAIC	Downregulated RNA In Cancer, Inhibitor Of Cell Invasion And Migration	1.14
PCAT29	Prostate Cancer Associated Transcript 29	1.14
ALDH1L1-AS2	ALDH1L1 Antisense RNA 2	1.14
LINC01426	Long Intergenic Non-Protein Coding RNA 1426	1.14
LINC00670	Long Intergenic Non-Protein Coding RNA 670	1.14
FAM53B-AS1	FAM53B Antisense RNA 1	1.14
MIR4425	MicroRNA 4425	1.14
AADACL2-AS1	AADACL2 Antisense RNA 1	1.14
MKLN1-AS	MKLN1 Antisense RNA	1.14
MIR193BHG	MIR193B Host Gene	1.14
LINC01812	Long Intergenic Non-Protein Coding RNA 1812	1.14
LINC01247	Long Intergenic Non-Protein Coding RNA 1247	1.14
GORAB-AS1	GORAB Antisense RNA 1	1.14
EPS15P1	Epidermal Growth Factor Receptor Pathway Substrate 15 Pseudogene 1	1.14
ENSG00000229162		1.14
ENSG00000231953		1.14
ENSG00000256234		1.14
ENSG00000257258		1.14
ENSG00000258539		1.14
ENSG00000253811		1.14
ENSG00000253456		1.14
ENSG00000270460		1.14
ENSG00000205653		1.14
RPL36AP43	Ribosomal Protein L36a Pseudogene 43	1.14
ENSG00000255750		1.14
ENSG00000261025		1.14
LINC02419	Long Intergenic Non-Protein Coding RNA 2419	1.14
HAUS4P1	HAUS Augmin Like Complex Subunit 4 Pseudogene 1	1.14
ENSG00000239941		1.14
ENSG00000226992		1.14
ENSG00000238034		1.14
ENSG00000225718		1.14
ENSG00000232072		1.14
ENSG00000233689		1.14
LOC105376204	Uncharacterized LOC105376204	1.14
ENSG00000230575		1.14
ENSG00000255968		1.14

ENSG00000250934		1.14
TDGP1	Thymine-DNA Glycosylase Pseudogene 1	1.14
RNU6-229P	RNA, U6 Small Nuclear 229, Pseudogene	1.14
LINC01824	Long Intergenic Non-Protein Coding RNA 1824	1.14
lnc-RNF145-3		1.14
RNA5SP56	RNA, 5S Ribosomal Pseudogene 56	1.14
RNU6-187P	RNA, U6 Small Nuclear 187, Pseudogene	1.14
RNU6-832P	RNA, U6 Small Nuclear 832, Pseudogene	1.14
RNU6-394P	RNA, U6 Small Nuclear 394, Pseudogene	1.14
MYCLP2	MYCL Pseudogene 2	1.14
ENSG00000200857		1.14
LOC105373757	Uncharacterized LOC105373757	1.14
PSMC1P12	Proteasome 26S Subunit, ATPase 1 Pseudogene 12	1.14
ENSG00000255451		1.14
KRT8P17	Keratin 8 Pseudogene 17	1.14
ENSG00000253949		1.14
ENSG00000278095		1.14
ENSG00000284999		1.14
lnc-TLE3-15		1.14
AY509916		1.14
RF00017-6259		1.14
piR-48389-002		1.14
lnc-RUNX3-3		1.14
ENSG00000286215		1.14
ENSG00000285785		1.14
ENSG00000286364		1.14
ENSG00000286748		1.14
lnc-RNF145-4		1.14
ENSG00000286153		1.14
lnc-SYF2-5		1.14
lnc-FOXL2NB-8		1.14
HSALNG0047286		1.14
RNU6ATAC21P	RNA, U6atac Small Nuclear 21, Pseudogene	1.14
lnc-MEMO1-3		1.14
piR-54649		1.14
lnc-KLF15-3		1.14
NOLC1P1	NOLC1 Pseudogene 1	1.14
ENSG00000201075		1.14
HSALNG0073494		1.14
NONHSAG006279.2		1.14

LOC105369705	Uncharacterized LOC105369705	1.14
ENSG00000256625		1.14
ENSG00000279076		1.14
lnc-TLE3-16		1.14
ENSG00000285896		1.14
piR-39099-217		1.14
ENSG00000286466		1.14
lnc-RCAN1-5		1.14
lnc-ATG5-9		1.14
piR-58297-353		1.14
HSALNG0001769		1.14
piR-54420		1.14
ENSG00000237617		1.14
IFITM3P7	IFITM3 Pseudogene 7	1.14
LOC107984111	Uncharacterized LOC107984111	1.14
piR-38352-105		1.14
ENSG00000279193		1.14
ENSG00000271355		1.14
RF00017-6262		1.14
FJ601684-005		1.14
piR-57195-017		1.14
ENSG00000286417		1.14
HSALNG0050307		1.14
LOC102724584	Uncharacterized LOC102724584	1.14
LOC105373882	Uncharacterized LOC105373882	1.14
ENSG00000235968		1.14
LOC105369704	Uncharacterized LOC105369704	1.14
LOC107984788	Uncharacterized LOC107984788	1.14
piR-47086-011		1.14
piR-50444-402		1.14
ENSG00000278687		1.14
ENSG00000226280		1.14
PRKCQ	Protein Kinase C Theta	0.99
RORA	RAR Related Orphan Receptor A	0.99
CRHR1	Corticotropin Releasing Hormone Receptor 1	0.99
SLC17A5	Solute Carrier Family 17 Member 5	0.87
TYMP	Thymidine Phosphorylase	0.83
ERBB3	Erb-B2 Receptor Tyrosine Kinase 3	0.8
IDH2	Isocitrate Dehydrogenase (NADP(+)) 2	0.8
ALDH2	Aldehyde Dehydrogenase 2 Family Member	0.8
KCNMA1	Potassium Calcium-Activated Channel Subfamily M Alpha 1	0.8

CYP1B1	Cytochrome P450 Family 1 Subfamily B Member 1	0.8
NMNAT1	Nicotinamide Nucleotide Adenylyltransferase 1	0.8
CACNA1A	Calcium Voltage-Gated Channel Subunit Alpha1 A	0.8
ATP2B3	ATPase Plasma Membrane Ca2+ Transporting 3	0.8
MSN	Moesin	0.8
CSNK2B	Casein Kinase 2 Beta	0.8
POU5F1	POU Class 5 Homeobox 1	0.8
PAX3	Paired Box 3	0.8
SCNN1A	Sodium Channel Epithelial 1 Subunit Alpha	0.8
PLCB3	Phospholipase C Beta 3	0.8
TNFSF11	TNF Superfamily Member 11	0.8
PIK3CB	Phosphatidylinositol-4,5-Bisphosphate 3-Kinase Catalytic Subunit Beta	0.8
TCF12	Transcription Factor 12	0.8
USP15	Ubiquitin Specific Peptidase 15	0.8
ATF1	Activating Transcription Factor 1	0.8
PRKAR1B	Protein Kinase CAMP-Dependent Type I Regulatory Subunit Beta	0.8
TCF4	Transcription Factor 4	0.8
SUCLA2	Succinate-CoA Ligase ADP-Forming Subunit Beta	0.8
MCM3	Minichromosome Maintenance Complex Component 3	0.8
BCL2L11	BCL2 Like 11	0.8
CDKN2C	Cyclin Dependent Kinase Inhibitor 2C	0.8
CAST	Calpastatin	0.8
GRM8	Glutamate Metabotropic Receptor 8	0.8
NDRG1	N-Myc Downstream Regulated 1	0.8
PSMB7	Proteasome 20S Subunit Beta 7	0.8
TNFRSF21	TNF Receptor Superfamily Member 21	0.8
VDAC1	Voltage Dependent Anion Channel 1	0.8
MASP2	Mannan Binding Lectin Serine Peptidase 2	0.8
MAP3K14	Mitogen-Activated Protein Kinase Kinase Kinase 14	0.8
FADS1	Fatty Acid Desaturase 1	0.8
FADS2	Fatty Acid Desaturase 2	0.8
FZD10	Frizzled Class Receptor 10	0.8
CYP2C18	Cytochrome P450 Family 2 Subfamily C Member 18	0.8
CRADD	CASP2 And RIPK1 Domain Containing Adaptor With Death Domain	0.8
CHD4	Chromodomain Helicase DNA Binding Protein 4	0.8
DYRK2	Dual Specificity Tyrosine Phosphorylation Regulated Kinase 2	0.8
NANS	N-Acetylneuraminate Synthase	0.8
PDGFA	Platelet Derived Growth Factor Subunit A	0.8
PFKFB3	6-Phosphofructo-2-Kinase/Fructose-2,6-Biphosphatase 3	0.8
NLRC4	NLR Family CARD Domain Containing 4	0.8

SLC7A5	Solute Carrier Family 7 Member 5	0.8
NSF	N-Ethylmaleimide Sensitive Factor, Vesicle Fusing ATPase	0.8
TMPRSS2	Transmembrane Serine Protease 2	0.8
NDUFA12	NADH:Ubiquinone Oxidoreductase Subunit A12	0.8
UBE2G2	Ubiquitin Conjugating Enzyme E2 G2	0.8
VEGFB	Vascular Endothelial Growth Factor B	0.8
VAMP1	Vesicle Associated Membrane Protein 1	0.8
LITAF	Lipopolysaccharide Induced TNF Factor	0.8
ATG7	Autophagy Related 7	0.8
CD82	CD82 Molecule	0.8
PRKAG3	Protein Kinase AMP-Activated Non-Catalytic Subunit Gamma 3	0.8
IQGAP1	IQ Motif Containing GTPase Activating Protein 1	0.8
ST3GAL1	ST3 Beta-Galactoside Alpha-2,3-Sialyltransferase 1	0.8
STK36	Serine/Threonine Kinase 36	0.8
DCPS	Decapping Enzyme, Scavenger	0.8
EGR2	Early Growth Response 2	0.8
TFAP2C	Transcription Factor AP-2 Gamma	0.8
VAPA	VAMP Associated Protein A	0.8
GSC	Goosecoid Homeobox	0.8
EED	Embryonic Ectoderm Development	0.8
SP3	Sp3 Transcription Factor	0.8
RAB5B	RAB5B, Member RAS Oncogene Family	0.8
GALNT14	Polypeptide N-Acetylgalactosaminyltransferase 14	0.8
CAMK1D	Calcium/Calmodulin Dependent Protein Kinase ID	0.8
ARHGEF9	Cdc42 Guanine Nucleotide Exchange Factor 9	0.8
AP4B1	Adaptor Related Protein Complex 4 Subunit Beta 1	0.8
LPAR5	Lysophosphatidic Acid Receptor 5	0.8
LTBR	Lymphotoxin Beta Receptor	0.8
BIRC6	Baculoviral IAP Repeat Containing 6	0.8
HOXD13	Homeobox D13	0.8
CLIC4	Chloride Intracellular Channel 4	0.8
HHAT	Hedgehog Acyltransferase	0.8
MSI2	Musashi RNA Binding Protein 2	0.8
DGKH	Diacylglycerol Kinase Eta	0.8
IL15RA	Interleukin 15 Receptor Subunit Alpha	0.8
PCBP1	Poly(RC) Binding Protein 1	0.8
S100A11	S100 Calcium Binding Protein A11	0.8
ST3GAL4	ST3 Beta-Galactoside Alpha-2,3-Sialyltransferase 4	0.8
KIRREL3	Kirre Like Nephrin Family Adhesion Molecule 3	0.8
CIB1	Calcium And Integrin Binding 1	0.8
MSH5	MutS Homolog 5	0.8

TRIM27	Tripartite Motif Containing 27	0.8
VPS33B	VPS33B Late Endosome And Lysosome Associated	0.8
HOXD10	Homeobox D10	0.8
HOXD9	Homeobox D9	0.8
FOXL2	Forkhead Box L2	0.8
HIPK1	Homeodomain Interacting Protein Kinase 1	0.8
SPAST	Spastin	0.8
NCAPD2	Non-SMC Condensin I Complex Subunit D2	0.8
POLA2	DNA Polymerase Alpha 2, Accessory Subunit	0.8
NUBP1	Nucleotide Binding Protein 1	0.8
PHB2	Prohibitin 2	0.8
PHLPP1	PH Domain And Leucine Rich Repeat Protein Phosphatase 1	0.8
SYVN1	Synoviolin 1	0.8
SUPT3H	SPT3 Homolog, SAGA And STAGA Complex Component	0.8
DNAH5	Dynein Axonemal Heavy Chain 5	0.8
DHX16	DEAH-Box Helicase 16	0.8
CCDC6	Coiled-Coil Domain Containing 6	0.8
MAN1C1	Mannosidase Alpha Class 1C Member 1	0.8
FAIM	Fas Apoptotic Inhibitory Molecule	0.8
FAU	FAU Ubiquitin Like And Ribosomal Protein S30 Fusion	0.8
ABCF1	ATP Binding Cassette Subfamily F Member 1	0.8
HOXD8	Homeobox D8	0.8
HAS1	Hyaluronan Synthase 1	0.8
DIP2B	Disco Interacting Protein 2 Homolog B	0.8
DDX39B	DExD-Box Helicase 39B	0.8
CXXC4	CXXC Finger Protein 4	0.8
ELAVL4	ELAV Like RNA Binding Protein 4	0.8
PBX2	PBX Homeobox 2	0.8
PRC1	Protein Regulator Of Cytokinesis 1	0.8
RRAGB	Ras Related GTP Binding B	0.8
PLXNC1	Plexin C1	0.8
SLC52A3	Solute Carrier Family 52 Member 3	0.8
SLC16A9	Solute Carrier Family 16 Member 9	0.8
SEMA4B	Semaphorin 4B	0.8
ILKAP	ILK Associated Serine/Threonine Phosphatase	0.8
SRSF7	Serine And Arginine Rich Splicing Factor 7	0.8
ULBP2	UL16 Binding Protein 2	0.8
KLHL20	Kelch Like Family Member 20	0.8
LGR4	Leucine Rich Repeat Containing G Protein-Coupled Receptor 4	0.8
MYO10	Myosin X	0.8
CD180	CD180 Molecule	0.8

PRDM6	PR/SET Domain 6	0.8
VIL1	Villin 1	0.8
CCHCR1	Coiled-Coil Alpha-Helical Rod Protein 1	0.8
ARHGAP21	Rho GTPase Activating Protein 21	0.8
FEZ1	Fasciculation And Elongation Protein Zeta 1	0.8
COX7C	Cytochrome C Oxidase Subunit 7C	0.8
HEXIM1	HEXIM P-TEFb Complex Subunit 1	0.8
GRHL3	Grainyhead Like Transcription Factor 3	0.8
SF1	Splicing Factor 1	0.8
PPP1R10	Protein Phosphatase 1 Regulatory Subunit 10	0.8
RSPO3	R-Spondin 3	0.8
TNFSF18	TNF Superfamily Member 18	0.8
TNS3	Tensin 3	0.8
SCYL3	SCY1 Like Pseudokinase 3	0.8
FRMD4A	FERM Domain Containing 4A	0.8
C1D	C1D Nuclear Receptor Corepressor	0.8
FAAH2	Fatty Acid Amide Hydrolase 2	0.8
BANP	BTG3 Associated Nuclear Protein	0.8
ESPN	Espin	0.8
ANP32B	Acidic Nuclear Phosphoprotein 32 Family Member B	0.8
LPIN3	Lipin 3	0.8
FAM136A	Family With Sequence Similarity 136 Member A	0.8
ATF6B	Activating Transcription Factor 6 Beta	0.8
LRRC32	Leucine Rich Repeat Containing 32	0.8
ADO	2-Aminoethanethiol Dioxygenase	0.8
AKAP11	A-Kinase Anchoring Protein 11	0.8
ACBD4	Acyl-CoA Binding Domain Containing 4	0.8
FMNL1	Formin Like 1	0.8
HOXD11	Homeobox D11	0.8
CEBPZ	CCAAT Enhancer Binding Protein Zeta	0.8
CNTN3	Contactin 3	0.8
CHD6	Chromodomain Helicase DNA Binding Protein 6	0.8
RNF25	Ring Finger Protein 25	0.8
CSMD1	CUB And Sushi Multiple Domains 1	0.8
EHD3	EH Domain Containing 3	0.8
PCYOX1	Prenylcysteine Oxidase 1	0.8
PLEKHH2	Pleckstrin Homology, MyTH4 And FERM Domain Containing H2	0.8
SOX13	SRY-Box Transcription Factor 13	0.8
SNRPG	Small Nuclear Ribonucleoprotein Polypeptide G	0.8
NFKBIL1	NFKB Inhibitor Like 1	0.8
OLFML3	Olfactomedin Like 3	0.8

LCLAT1	Lysocardiolipin Acyltransferase 1	0.8
LAG3	Lymphocyte Activating 3	0.8
TRMT11	TRNA Methyltransferase 11 Homolog	0.8
LIN7C	Lin-7 Homolog C, Crumbs Cell Polarity Complex Component	0.8
SYT14	Synaptotagmin 14	0.8
RCAN3	RCAN Family Member 3	0.8
KATNAL1	Katanin Catalytic Subunit A1 Like 1	0.8
PRSS23	Serine Protease 23	0.8
ZC4H2	Zinc Finger C4H2-Type Containing	0.8
ZNF365	Zinc Finger Protein 365	0.8
CCDC68	Coiled-Coil Domain Containing 68	0.8
FBXO31	F-Box Protein 31	0.8
LPCAT3	Lysophosphatidylcholine Acyltransferase 3	0.8
ARHGAP27	Rho GTPase Activating Protein 27	0.8
ERGIC2	ERGIC And Golgi 2	0.8
ADGRF5	Adhesion G Protein-Coupled Receptor F5	0.8
CRISPLD2	Cysteine Rich Secretory Protein LCCL Domain Containing 2	0.8
HOXD1	Homeobox D1	0.8
CEP70	Centrosomal Protein 70	0.8
CORT	Cortistatin	0.8
HEXIM2	HEXIM P-TEFb Complex Subunit 2	0.8
DHX57	DExH-Box Helicase 57	0.8
MRPS30	Mitochondrial Ribosomal Protein S30	0.8
DNAAF5	Dynein Axonemal Assembly Factor 5	0.8
NAALADL1	N-Acetylated Alpha-Linked Acidic Dipeptidase Like 1	0.8
NAPG	NSF Attachment Protein Gamma	0.8
CDC42EP4	CDC42 Effector Protein 4	0.8
RNF11	Ring Finger Protein 11	0.8
CREBZF	CREB/ATF BZIP Transcription Factor	0.8
IGSF10	Immunoglobulin Superfamily Member 10	0.8
NOP2	NOP2 Nucleolar Protein	0.8
SDK1	Sidekick Cell Adhesion Molecule 1	0.8
RPUSD4	RNA Pseudouridine Synthase D4	0.8
SLC35B3	Solute Carrier Family 35 Member B3	0.8
SPSB2	SplA/Ryanodine Receptor Domain And SOCS Box Containing 2	0.8
TREX2	Three Prime Repair Exonuclease 2	0.8
UBL3	Ubiquitin Like 3	0.8
UBLCP1	Ubiquitin Like Domain Containing CTD Phosphatase 1	0.8
TRMT112	TRNA Methyltransferase Subunit 11-2	0.8
LHX2	LIM Homeobox 2	0.8
STARD8	StAR Related Lipid Transfer Domain Containing 8	0.8

TCF19	Transcription Factor 19	0.8
RBP7	Retinol Binding Protein 7	0.8
TMEM132D	Transmembrane Protein 132D	0.8
ING4	Inhibitor Of Growth Family Member 4	0.8
LAX1	Lymphocyte Transmembrane Adaptor 1	0.8
PAQR8	Progestin And AdipoQ Receptor Family Member 8	0.8
KIF18A	Kinesin Family Member 18A	0.8
ZC3H11A	Zinc Finger CCCH-Type Containing 11A	0.8
ZDHHC7	Zinc Finger DHHC-Type Palmitoyltransferase 7	0.8
ZNF384	Zinc Finger Protein 384	0.8
VGLL4	Vestigial Like Family Member 4	0.8
ZBTB38	Zinc Finger And BTB Domain Containing 38	0.8
VSIG4	V-Set And Immunoglobulin Domain Containing 4	0.8
GNL3L	G Protein Nucleolar 3 Like	0.8
CCDC91	Coiled-Coil Domain Containing 91	0.8
ATG2A	Autophagy Related 2A	0.8
FEV	FEV Transcription Factor, ETS Family Member	0.8
MAGI3	Membrane Associated Guanylate Kinase, WW And PDZ Domain Containing 3	0.8
BBX	BBX High Mobility Group Box Domain Containing	0.8
GADL1	Glutamate Decarboxylase Like 1	0.8
HOXD12	Homeobox D12	0.8
HOXD4	Homeobox D4	0.8
HEMGN	Hemogen	0.8
DHRS7B	Dehydrogenase/Reductase 7B	0.8
RNF145	Ring Finger Protein 145	0.8
PLEKHG6	Pleckstrin Homology And RhoGEF Domain Containing G6	0.8
PHTF1	Putative Homeodomain Transcription Factor 1	0.8
TRAF3IP3	TRAF3 Interacting Protein 3	0.8
TPRG1	Tumor Protein P63 Regulated 1	0.8
TAPBPL	TAP Binding Protein Like	0.8
LBH	LBH Regulator Of WNT Signaling Pathway	0.8
ZHX3	Zinc Fingers And Homeoboxes 3	0.8
ESPNL	Espin Like	0.8
ACRBP	Acrosin Binding Protein	0.8
MYRF	Myelin Regulatory Factor	0.8
IL26	Interleukin 26	0.8
PRM2	Protamine 2	0.8
UNC45A	Unc-45 Myosin Chaperone A	0.8
KIAA0513	KIAA0513	0.8
TCHHL1	Trichohyalin Like 1	0.8

TMCC3	Transmembrane And Coiled-Coil Domain Family 3	0.8
YIPF4	Yip1 Domain Family Member 4	0.8
YIPF6	Yip1 Domain Family Member 6	0.8
GET4	Guided Entry Of Tail-Anchored Proteins Factor 4	0.8
CAPN14	Calpain 14	0.8
EVX2	Even-Skipped Homeobox 2	0.8
FAM118B	Family With Sequence Similarity 118 Member B	0.8
BCL2L15	BCL2 Like 15	0.8
METTL18	Methyltransferase Like 18	0.8
ACOXL	Acyl-CoA Oxidase Like	0.8
AADACL2	Arylacetamide Deacetylase Like 2	0.8
C5orf15	Chromosome 5 Open Reading Frame 15	0.8
C2orf42	Chromosome 2 Open Reading Frame 42	0.8
C17orf80	Chromosome 17 Open Reading Frame 80	0.8
PRRC2C	Proline Rich Coiled-Coil 2C	0.8
IER2	Immediate Early Response 2	0.8
IFFO1	Intermediate Filament Family Orphan 1	0.8
RSBN1	Round Spermatid Basic Protein 1	0.8
NGRN	Neugrin, Neurite Outgrowth Associated	0.8
NIPAL3	NIPA Like Domain Containing 3	0.8
PSMG2	Proteasome Assembly Chaperone 2	0.8
TNP2	Transition Protein 2	0.8
TTLL11	Tubulin Tyrosine Ligase Like 11	0.8
TTLL4	Tubulin Tyrosine Ligase Like 4	0.8
KLHL36	Kelch Like Family Member 36	0.8
ZFPL1	Zinc Finger Protein Like 1	0.8
ZCCHC14	Zinc Finger CCHC-Type Containing 14	0.8
ZNF311	Zinc Finger Protein 311	0.8
ETAA1	ETAA1 Activator Of ATR Kinase	0.8
FAM104A	Family With Sequence Similarity 104 Member A	0.8
FAM110A	Family With Sequence Similarity 110 Member A	0.8
ADGRF1	Adhesion G Protein-Coupled Receptor F1	0.8
C1orf112	Chromosome 1 Open Reading Frame 112	0.8
RMDN2	Regulator Of Microtubule Dynamics 2	0.8
HEATR5B	HEAT Repeat Containing 5B	0.8
HINT3	Histidine Triad Nucleotide Binding Protein 3	0.8
HAUS7	HAUS Augmin Like Complex Subunit 7	0.8
EMC8	ER Membrane Protein Complex Subunit 8	0.8
NRM	Nurim	0.8
SELENON	Selenoprotein N	0.8
ICE2	Interactor Of Little Elongation Complex ELL Subunit 2	0.8

OR2J2	Olfactory Receptor Family 2 Subfamily J Member 2	0.8
OR2J3	Olfactory Receptor Family 2 Subfamily J Member 3	0.8
OR2B3	Olfactory Receptor Family 2 Subfamily B Member 3	0.8
TTC27	Tetratricopeptide Repeat Domain 27	0.8
TBC1D12	TBC1 Domain Family Member 12	0.8
UTP23	UTP23 Small Subunit Processome Component	0.8
USPL1	Ubiquitin Specific Peptidase Like 1	0.8
GALNT17	Polypeptide N-Acetylgalactosaminyltransferase 17	0.8
FAM9B	Family With Sequence Similarity 9 Member B	0.8
CPSF4L	Cleavage And Polyadenylation Specific Factor 4 Like	0.8
CITED4	Cbp/P300 Interacting Transactivator With Glu/Asp Rich Carboxy-Terminal Domain 4	0.8
EFCAB5	EF-Hand Calcium Binding Domain 5	0.8
CCDC34	Coiled-Coil Domain Containing 34	0.8
EMILIN3	Elastin Microfibril Interfacer 3	0.8
SPIN3	Spindlin Family Member 3	0.8
SERTAD4	SERTA Domain Containing 4	0.8
EMSY	EMSY Transcriptional Repressor, BRCA2 Interacting	0.8
POLR1H	RNA Polymerase I Subunit H	0.8
PSORS1C2	Psoriasis Susceptibility 1 Candidate 2	0.8
STPG1	Sperm Tail PG-Rich Repeat Containing 1	0.8
TMEM258	Transmembrane Protein 258	0.8
TMEM26	Transmembrane Protein 26	0.8
ZC3H12B	Zinc Finger CCCH-Type Containing 12B	0.8
METTL11B	Methyltransferase Like 11B	0.8
FAM9A	Family With Sequence Similarity 9 Member A	0.8
CNOT9	CCR4-NOT Transcription Complex Subunit 9	0.8
NCMAP	Non-Compact Myelin Associated Protein	0.8
ITIH6	Inter-Alpha-Trypsin Inhibitor Heavy Chain Family Member 6	0.8
STH	Saitohin	0.8
C6orf47	Chromosome 6 Open Reading Frame 47	0.8
GDPGP1	GDP-D-Glucose Phosphorylase 1	0.8
CCDC81	Coiled-Coil Domain Containing 81	0.8
ARL17A	ADP Ribosylation Factor Like GTPase 17A	0.8
LY6G5B	Lymphocyte Antigen 6 Family Member G5B	0.8
LRRC37A2	Leucine Rich Repeat Containing 37 Member A2	0.8
HCP5	HLA Complex P5	0.8
DEXI	Dexi Homolog	0.8
MRTFB	Myocardin Related Transcription Factor B	0.8
PAGE2B	PAGE Family Member 2B	0.8
PAGE5	PAGE Family Member 5	0.8

PRM3	Protamine 3	0.8
ZNF774	Zinc Finger Protein 774	0.8
MEDAG	Mesenteric Estrogen Dependent Adipogenesis	0.8
ABRAXAS2	Abraxas 2, BRISC Complex Subunit	0.8
TAFA2	TAFA Chemokine Like Family Member 2	0.8
TRMO	TRNA Methyltransferase O	0.8
ZFP92	ZFP92 Zinc Finger Protein	0.8
ARL17B	ADP Ribosylation Factor Like GTPase 17B	0.8
C1orf74	Chromosome 1 Open Reading Frame 74	0.8
SFTA2	Surfactant Associated 2	0.8
PAGE3	PAGE Family Member 3	0.8
PHETA1	PH Domain Containing Endocytic Trafficking Adaptor 1	0.8
KRTAP10-1	Keratin Associated Protein 10-1	0.8
ZBED6	Zinc Finger BED-Type Containing 6	0.8
MEAK7	MTOR Associated Protein, Eak-7 Homolog	0.8
PRR23B	Proline Rich 23B	0.8
SPATA32	Spermatogenesis Associated 32	0.8
HLA-H	Major Histocompatibility Complex, Class I, H (Pseudogene)	0.8
GRAMD2B	GRAM Domain Containing 2B	0.8
KRTAP10-2	Keratin Associated Protein 10-2	0.8
UTP25	UTP25 Small Subunit Processor Component	0.8
C1orf185	Chromosome 1 Open Reading Frame 185	0.8
MUC22	Mucin 22	0.8
TTLL13P	Tubulin Tyrosine Ligase Like 13, Pseudogene	0.8
HCG27	HLA Complex Group 27	0.8
HCG9	HLA Complex Group 9	0.8
PSORS1C3	Psoriasis Susceptibility 1 Candidate 3	0.8
CENPS-CORT	CENPS-CORT Readthrough	0.8
HCG22	HLA Complex Group 22	0.8
LINC00311	Long Intergenic Non-Protein Coding RNA 311	0.8
CYP1B1-AS1	CYP1B1 Antisense RNA 1	0.8
SCARNA12	Small Cajal Body-Specific RNA 12	0.8
SERTAD4-AS1	SERTAD4 Antisense RNA 1	0.8
LINC00303	Long Intergenic Non-Protein Coding RNA 303	0.8
TSPEAR-AS2	TSPEAR Antisense RNA 2	0.8
MTRNR2L10	MT-RNR2 Like 10	0.8
MIR4435-2HG	MIR4435-2 Host Gene	0.8
LINC00322	Long Intergenic Non-Protein Coding RNA 322	0.8
GVQW3	GVQW Motif Containing 3	0.8
IFNG-AS1	IFNG Antisense RNA 1	0.8
SCARNA11	Small Cajal Body-Specific RNA 11	0.8

TEX41	Testis Expressed 41	0.8
LINC01553	Long Intergenic Non-Protein Coding RNA 1553	0.8
ZFHX4-AS1	ZFHX4 Antisense RNA 1	0.8
FGF10-AS1	FGF10 Antisense RNA 1	0.8
MTLN	Mitoregulin	0.8
MIR558	MicroRNA 558	0.8
MIR603	MicroRNA 603	0.8
HCG4B	HLA Complex Group 4B	0.8
CD27-AS1	CD27 Antisense RNA 1	0.8
LINC00111	Long Intergenic Non-Protein Coding RNA 111	0.8
ZNRD1ASP	Zinc Ribbon Domain Containing 1 Antisense, Pseudogene	0.8
LINC01550	Long Intergenic Non-Protein Coding RNA 1550	0.8
ADAM1A	ADAM Metallopeptidase Domain 1A (Pseudogene)	0.8
C1RL-AS1	C1RL Antisense RNA 1	0.8
RORA-AS1	RORA Antisense RNA 1	0.8
PTCSC2	Papillary Thyroid Carcinoma Susceptibility Candidate 2	0.8
TSPEAR-AS1	TSPEAR Antisense RNA 1	0.8
LINC01494	Long Intergenic Non-Protein Coding RNA 1494	0.8
ACOXL-AS1	ACOXL Antisense RNA 1	0.8
CT66	Cancer/Testis Associated Transcript 66	0.8
RMDN2-AS1	RMDN2 Antisense RNA 1	0.8
MIR4478	MicroRNA 4478	0.8
IFITM4P	Interferon Induced Transmembrane Protein 4 Pseudogene	0.8
LINC01117	Long Intergenic Non-Protein Coding RNA 1117	0.8
LINC01107	Long Intergenic Non-Protein Coding RNA 1107	0.8
LINC01424	Long Intergenic Non-Protein Coding RNA 1424	0.8
LINC01563	Long Intergenic Non-Protein Coding RNA 1563	0.8
LINC00845	Long Intergenic Non-Protein Coding RNA 845	0.8
TCF4-AS1	TCF4 Antisense RNA 1	0.8
LINC01623	Long Intergenic Non-Protein Coding RNA 1623	0.8
LINC02610	Long Intergenic Non-Protein Coding RNA 2610	0.8
MICD	MHC Class I Polypeptide-Related Sequence D (Pseudogene)	0.8
MIR4432HG	MIR4432 Host Gene	0.8
MIR4765	MicroRNA 4765	0.8
SLC44A3-AS1	SLC44A3 Antisense RNA 1	0.8
LINC00384	Long Intergenic Non-Protein Coding RNA 384	0.8
MIR4421	MicroRNA 4421	0.8
TH2LCRR	T Helper Type 2 Locus Control Region Associated RNA	0.8
LINC02202	Long Intergenic Non-Protein Coding RNA 2202	0.8
LINC01936	Long Intergenic Non-Protein Coding RNA 1936	0.8
LINC01940	Long Intergenic Non-Protein Coding RNA 1940	0.8

LINC01427	Long Intergenic Non-Protein Coding RNA 1427	0.8
WARS2-AS1	WARS2 Antisense RNA 1	0.8
ATP2C2-AS1	ATP2C2 Antisense RNA 1	0.8
ETF1P1	Eukaryotic Translation Termination Factor 1 Pseudogene 1	0.8
LOC100129603	Uncharacterized LOC100129603	0.8
LOC100131107	Putative UPF0607 Protein ENSP00000383783	0.8
CCDC37-DT	CCDC37 Divergent Transcript	0.8
ENSG00000229618		0.8
ENSG00000248752		0.8
MIR6891	MicroRNA 6891	0.8
LINC01995	Long Intergenic Non-Protein Coding RNA 1995	0.8
LINC02016	Long Intergenic Non-Protein Coding RNA 2016	0.8
LGR4-AS1	LGR4 Antisense RNA 1	0.8
LINC02341	Long Intergenic Non-Protein Coding RNA 2341	0.8
LINC02421	Long Intergenic Non-Protein Coding RNA 2421	0.8
LINC02612	Long Intergenic Non-Protein Coding RNA 2612	0.8
FABP3P2	Fatty Acid Binding Protein 3 Pseudogene 2	0.8
CYCSP38	CYCS Pseudogene 38	0.8
HRAT92	Heart Tissue-Associated Transcript 92	0.8
FOXL3	Forkhead Box L3	0.8
MIR548AP	MicroRNA 548ap	0.8
HNRNPCP4	Heterogeneous Nuclear Ribonucleoprotein C Pseudogene 4	0.8
HDAC9-AS1	HDAC9 Antisense RNA 1	0.8
LOC100287387	Uncharacterized LOC100287387	0.8
RN7SL333P	RNA, 7SL, Cytoplasmic 333, Pseudogene	0.8
ENSG00000236935		0.8
PRKAR1B-AS2	PRKAR1B Antisense RNA 2	0.8
RPSAP4	Ribosomal Protein SA Pseudogene 4	0.8
ENSG00000224228		0.8
RPL3P2	Ribosomal Protein L3 Pseudogene 2	0.8
ENSG00000231424		0.8
ENSG00000231128		0.8
ENSG00000234471		0.8
ENSG00000234810		0.8
ENSG00000234921		0.8
ENSG00000226622		0.8
ENSG00000256967		0.8
ENSG00000258035		0.8
ENSG00000257449		0.8
ENSG00000258365		0.8
LINC00385	Long Intergenic Non-Protein Coding RNA 385	0.8

ENSG00000248975		0.8
STK19B	Serine/Threonine Kinase 19B (Pseudogene)	0.8
ENSG00000255317		0.8
ENSG00000255062		0.8
PCSEAT	Prostate Cancer Expressed EZH2 Associated Transcript	0.8
ENSG00000226375		0.8
ENSG00000259704		0.8
ENSG00000271716		0.8
ENSG00000271989		0.8
ENSG00000272501		0.8
ENSG00000260302		0.8
LINC01681	Long Intergenic Non-Protein Coding RNA 1681	0.8
ENSG00000259212		0.8
ENSG00000259274		0.8
LINC02224	Long Intergenic Non-Protein Coding RNA 2224	0.8
GOT2P2	GOT2 Pseudogene 2	0.8
ALDH7A1P4	Aldehyde Dehydrogenase 7 Family Member A1 Pseudogene 4	0.8
MIR4536-2	MicroRNA 4536-2	0.8
RNU6-474P	RNA, U6 Small Nuclear 474, Pseudogene	0.8
RNU1-103P	RNA, U1 Small Nuclear 103, Pseudogene	0.8
RN7SL381P	RNA, 7SL, Cytoplasmic 381, Pseudogene	0.8
RN7SL391P	RNA, 7SL, Cytoplasmic 391, Pseudogene	0.8
ENSG00000236501		0.8
ENSG00000237311		0.8
ENSG00000238280		0.8
ENSG00000217455		0.8
ENSG00000213994		0.8
SHC1P1	SHC Adaptor Protein 1 Pseudogene 1	0.8
ENSG00000231760		0.8
ENSG00000232080		0.8
ENSG00000212228		0.8
ENSG00000247121		0.8
ENSG00000248373		0.8
ENSG00000255966		0.8
ENSG00000254694		0.8
ENSG00000250421		0.8
ENSG00000249492		0.8
ENSG00000255389		0.8
ENSG00000255135		0.8
ENSG00000254810		0.8
MTCO3P1	MT-CO3 Pseudogene 1	0.8

ENSG00000228334		0.8
ENSG00000226849		0.8
ENSG00000236304		0.8
ENSG00000277726		0.8
ENSG00000270184		0.8
ENSG00000260859		0.8
ENSG00000263033		0.8
ENSG00000263307		0.8
LINC02793	Long Intergenic Non-Protein Coding RNA 2793	0.8
LINC02836	Long Intergenic Non-Protein Coding RNA 2836	0.8
LINC02745	Long Intergenic Non-Protein Coding RNA 2745	0.8
ENSG00000287658		0.8
AURKBP1	Aurora Kinase B Pseudogene 1	0.8
lnc-CCDC68-5		0.8
ENSG00000286084		0.8
RN7SL614P	RNA, 7SL, Cytoplasmic 614, Pseudogene	0.8
RNU6-351P	RNA, U6 Small Nuclear 351, Pseudogene	0.8
DDX39BP2	DEAD-Box Helicase 39B Pseudogene 2	0.8
RPL24P7	RPL24 Pseudogene 7	0.8
RNU6-929P	RNA, U6 Small Nuclear 929, Pseudogene	0.8
ENSG00000227486		0.8
ENSG00000239665		0.8
ENSG00000223598		0.8
ENSG00000205037		0.8
ENSG00000219410		0.8
NRBF2P4	Nuclear Receptor Binding Factor 2 Pseudogene 4	0.8
ENSG00000231078		0.8
ENSG00000229528		0.8
ENSG00000254733		0.8
ENSG00000257880		0.8
PUDPP2	Pseudouridine 5'-Phosphatase Pseudogene 2	0.8
KATNBL1P2	Katanin Regulatory Subunit B1 Like 1 Pseudogene 2	0.8
ENSG00000247853		0.8
PPP1R11P2	Protein Phosphatase 1 Regulatory Inhibitor Subunit 11 Pseudogene 2	0.8
ENSG00000257176		0.8
RPL21P62	Ribosomal Protein L21 Pseudogene 62	0.8
ENSG00000225214		0.8
ENSG00000233755		0.8
ENSG00000253796		0.8
ENSG00000271382		0.8

ENSG00000271737		0.8
ENSG00000271811		0.8
ENSG00000272109		0.8
ENSG00000272221		0.8
ENSG00000272540		0.8
LINC01916	Long Intergenic Non-Protein Coding RNA 1916	0.8
ENSG00000261285		0.8
ENSG00000261468		0.8
ENSG00000262020		0.8
ENSG00000265554		0.8
ENSG00000259314		0.8
ENSG00000282246		0.8
ENSG00000264876		0.8
lnc-C17orf80-6		0.8
lnc-DFFA-7		0.8
CBX1P3	Chromobox 1 Pseudogene 3	0.8
lnc-CCDC68-2		0.8
lnc-CCDC68-4		0.8
lnc-SCNN1A-1		0.8
BMI1P1	BMI1 Proto-Oncogene, Polycomb Ring Finger Pseudogene 1	0.8
LOC112268270	Translation Initiation Factor IF-2-Like	0.8
lnc-RNF145-1		0.8
lnc-CPSF4L-1		0.8
RN7SL592P	RNA, 7SL, Cytoplasmic 592, Pseudogene	0.8
lnc-HLA-C-2		0.8
MTND1P31	MT-ND1 Pseudogene 31	0.8
LOC100420680	Coiled-Coil Domain Containing 90B Pseudogene	0.8
NANOGP9	Nanog Homeobox Pseudogene 9	0.8
RNU6-1047P	RNA, U6 Small Nuclear 1047, Pseudogene	0.8
piR-35448		0.8
ENSG00000228064		0.8
SALL4P5	Spalt Like Transcription Factor 4 Pseudogene 5	0.8
RPSAP25	Ribosomal Protein SA Pseudogene 25	0.8
LOC105369246	Uncharacterized LOC105369246	0.8
RPL21P24	Ribosomal Protein L21 Pseudogene 24	0.8
ENSG00000234862		0.8
ENSG00000235972		0.8
ENSG00000230552		0.8
ENSG00000207411		0.8
LOC105369781	Uncharacterized LOC105369781	0.8
ENSG00000248701		0.8

LOC107984859	Uncharacterized LOC107984859	0.8
ENSG00000249852		0.8
PGAM1P13	Phosphoglycerate Mutase 1 Pseudogene 13	0.8
ENSG00000226861		0.8
lnc-NDRG1-7		0.8
lnc-PEX14-7		0.8
ENSG00000258379		0.8
ENSG00000278716		0.8
ENSG00000270322		0.8
ENSG00000271581		0.8
ENSG00000272054		0.8
ENSG00000273118		0.8
ENSG00000260118		0.8
ENSG00000261349		0.8
ENSG00000267101		0.8
ENSG00000269256		0.8
YWHAZP7	Tyrosine 3-Monooxygenase/Tryptophan 5-Monooxygenase Activation Protein Zeta Pseudogene 7	0.8
ENSG00000287787		0.8
CCNYL5	Cyclin Y Like 5 (Pseudogene)	0.8
lnc-THNSL1-1		0.8
RF00017-7879		0.8
piR-57133-098		0.8
lnc-CCDC88B-1		0.8
lnc-BRAP-1		0.8
lnc-RUNX3-2		0.8
RF00017-5977		0.8
lnc-CITED4-1		0.8
lnc-SRPRA-7		0.8
lnc-ATP2B3-2		0.8
lnc-BBOX1-10		0.8
lnc-BCL6-9		0.8
RF00017-4207		0.8
ENSG00000286046		0.8
lnc-CRTC3-1		0.8
piR-49657		0.8
RF00017-8481		0.8
RF00017-8483		0.8
piR-49294		0.8
lnc-GDPGP1-5		0.8
CTBP2P2	CTBP2 Pseudogene 2	0.8

lnc-VWA8-7		0.8
lnc-CA5A-8		0.8
lnc-CA5A-9		0.8
lnc-TNFRSF1A-1		0.8
lnc-TNFSF18-1		0.8
lnc-FAM92B-1		0.8
lnc-ULBP1-2		0.8
lnc-FAM19A2-2		0.8
MN298114-200		0.8
piR-50534		0.8
lnc-ICE2-2		0.8
lnc-ICE2-4		0.8
lnc-IL2RA-5		0.8
LOC100128362	Protein Phosphatase 2 Catalytic Subunit Alpha Pseudogene	0.8
lnc-USPL1-2		0.8
lnc-UTP23-11		0.8
MN309431		0.8
MN309432		0.8
NONHSAG043472.2		0.8
ENSG00000237669		0.8
IFITM3P5	IFITM3 Pseudogene 5	0.8
RUNX3-AS1	RUNX3 Antisense RNA 1	0.8
HSALNG0096135		0.8
piR-45035-151		0.8
lnc-LHX2-3		0.8
piR-53177-032		0.8
lnc-KCNQ4-7		0.8
HSALNG0073495		0.8
lnc-JPH3-8		0.8
ENSG00000184115		0.8
lnc-KRTAP10-12-2		0.8
piR-47864		0.8
lnc-PPA2-10		0.8
lnc-PRDM1-7		0.8
lnc-PRKAR1B-1		0.8
ENSG00000252888		0.8
RF00017-1272		0.8
lnc-PLEKHG6-4		0.8
L13713-231		0.8
ENSG00000254755		0.8
RF00017-107		0.8

L13304-031		0.8
piR-52740		0.8
RSL24D1P7	Ribosomal L24 Domain Containing 1 Pseudogene 7	0.8
lnc-NDRG1-5		0.8
ENSG00000253801		0.8
ENSG00000278215		0.8
ENSG00000271018		0.8
ENSG00000271128		0.8
ENSG00000271396		0.8
ENSG00000273890		0.8
piR-40666-013		0.8
lnc-SNRPE-3		0.8
RF00017-6267		0.8
lnc-CCDC34-6		0.8
ENSG00000288583		0.8
ENSG00000287949		0.8
ENSG00000287563		0.8
FJ601684-136		0.8
lnc-CLIC4-2		0.8
5MWI_A-080		0.8
LOC112267915	Uncharacterized LOC112267915	0.8
LOC112268088	Uncharacterized LOC112268088	0.8
ENSG00000286147		0.8
piR-33338		0.8
RF00017-8487		0.8
lnc-GALNT17-6		0.8
piR-34749		0.8
RF00998-117		0.8
lnc-VGLL4-2		0.8
lnc-VEZT-10		0.8
piR-42741		0.8
piR-50444-431		0.8
lnc-TRMT11-2		0.8
lnc-EHD3-4		0.8
lnc-ZNF800-7		0.8
HSALNG0021699		0.8
HSALNG0122037		0.8
piR-38120		0.8
piR-38051-139		0.8
PPIAP67	Peptidylprolyl Isomerase A Pseudogene 67	0.8
NONHSAG028935.2		0.8

ENSG00000224981		0.8
piR-61532-025		0.8
HSALNG0109576		0.8
NONHSAG042527.2		0.8
ENSG00000213620		0.8
NONHSAG020184.2-002		0.8
piR-36830		0.8
lnc-LIN28B-11		0.8
HSALNG0133560		0.8
HSALNG0133399		0.8
HSALNG0131102		0.8
LOC105369780	Uncharacterized LOC105369780	0.8
RF00017-178		0.8
lnc-PPA2-9		0.8
ENSG00000256378		0.8
lnc-PRDX5-1		0.8
lnc-PREP-5		0.8
lnc-PRDM1-9		0.8
TRMT10BP1	TRNA Methyltransferase 10B Pseudogene 1	0.8
LOC107984526	Uncharacterized LOC107984526	0.8
ENSG00000251473		0.8
LOC107984500	Uncharacterized LOC107984500	0.8
piR-30444-140		0.8
piR-52680-323		0.8
HSALNG0014097		0.8
piR-43106-108		0.8
piR-52008-011		0.8
piR-54575		0.8
NONHSAG055547.2		0.8
lnc-NDRG1-3		0.8
piR-38051-325		0.8
ENSG00000270838		0.8
ENSG00000264131		0.8
ENSG00000280154		0.8
piR-31210-010		0.8
ENSG00000259608		0.8
piR-32214-114		0.8
lnc-CASZ1-4		0.8
piR-57133-593		0.8
EOS	Eosinophilia, Familial	0.8
piR-39858-296		0.8

RF00017-5252		0.8
RF00017-5981		0.8
lnc-SERPINB12-1		0.8
piR-32810-086		0.8
piR-57176-560		0.8
5MWI_A-082		0.8
5MWI_A-131		0.8
5MWI_A-192		0.8
ENSG00000286383		0.8
ENSG00000286764		0.8
lnc-BIRC6-3		0.8
lnc-SUCNR1-4		0.8
lnc-CENPS-CORT-4		0.8
lnc-CENPS-CORT-5		0.8
piR-57133-629		0.8
piR-51671-059		0.8
lnc-ZC4H2-1		0.8
lnc-ZC3H12B-15		0.8
RF00994-1030		0.8
lnc-TRMT11-5		0.8
lnc-EMC2-8		0.8
lnc-EPG5-10		0.8
piR-42085-004		0.8
RF00994-258		0.8
lnc-EHD3-7		0.8
LOC100533843	Karyopherin Subunit Alpha 2 Pseudogene	0.8
LOC100509370	39S Ribosomal Protein L21, Mitochondrial Pseudogene	0.8
piR-50308-096		0.8
lnc-TMEM74-2		0.8
lnc-ZNRD1-3		0.8
MN298114-179		0.8
LOC101928096	Uncharacterized LOC101928096	0.8
ENSG00000243016		0.8
HSALNG0122056		0.8
LOC105372813	Uncharacterized LOC105372813	0.8
piR-45693		0.8
HSALNG0119956		0.8
piR-38051-285		0.8
SNODB804		0.8
LOC105377944	Uncharacterized LOC105377944	0.8
ENSG00000216781		0.8

ENSG00000213148		0.8
lnc-LAS1L-5		0.8
piR-45012-450		0.8
piR-45012-575		0.8
ENSG00000232765		0.8
LOC105374875	Uncharacterized LOC105374875	0.8
hsa-miR-5095-290		0.8
piR-55194-088		0.8
piR-46501-096		0.8
piR-61945-117		0.8
HSALNG0131108		0.8
piR-36971		0.8
piR-46932		0.8
piR-30831-002		0.8
L13714-378		0.8
ENSG00000257360		0.8
piR-39098-157		0.8
LOC107984830	Uncharacterized LOC107984830	0.8
piR-30048-046		0.8
LOC107985712	Uncharacterized LOC107985712	0.8
piR-38344-035		0.8
lnc-EIF3E-1		0.8
HSALNG0036854		0.8
Hsa-Mir-544_3p-001		0.8
ENSG00000237971		0.8
ENSG00000276929		0.8
ENSG00000278104		0.8
ENSG00000279205		0.8
ENSG00000279499		0.8
ENSG00000270422		0.8
ENSG00000274075		0.8
ENSG00000236852		0.8
LOC105371689	Uncharacterized LOC105371689	0.8
ENSG00000218175		0.8
piR-61945-538		0.8
ENSG00000231356		0.8
LOC107986047	Uncharacterized LOC107986047	0.8
LOC107985966	Uncharacterized LOC107985966	0.8
ENSG00000253262		0.8
ENSG00000226820		0.8
ENSG00000233710		0.8

ENSG00000278319		0.8
ABCB1	ATP Binding Cassette Subfamily B Member 1	0.73
TUBB	Tubulin Beta Class I	0.73
ESR2	Estrogen Receptor 2	0.7
AKR1C4	Aldo-Keto Reductase Family 1 Member C4	0.67
DGAT1	Diacylglycerol O-Acyltransferase 1	0.67
PTGFR	Prostaglandin F Receptor	0.67
ARNTL	Aryl Hydrocarbon Receptor Nuclear Translocator Like	0.67
BAX	BCL2 Associated X, Apoptosis Regulator	0.59
TXNRD1	Thioredoxin Reductase 1	0.59
LIFR	LIF Receptor Subunit Alpha	0.59
AQP1	Aquaporin 1 (Colton Blood Group)	0.59
IL6ST	Interleukin 6 Signal Transducer	0.59
TIRAP	TIR Domain Containing Adaptor Protein	0.59
LAMA5	Laminin Subunit Alpha 5	0.59
FGF18	Fibroblast Growth Factor 18	0.59
SVIL	Supervillin	0.59
SHROOM3	Shroom Family Member 3	0.59
CGB5	Chorionic Gonadotropin Subunit Beta 5	0.59
MIR203A	MicroRNA 203a	0.59
LOC107880064	IVL Promoter Region	0.59
TYMS	Thymidylate Synthetase	0.59
DHODH	Dihydroorotate Dehydrogenase (Quinone)	0.42
CDK4	Cyclin Dependent Kinase 4	0.41
SMO	Smoothened, Frizzled Class Receptor	0.41
CTBP1	C-Terminal Binding Protein 1	0.41
COL4A1	Collagen Type IV Alpha 1 Chain	0.41
VCAN	Versican	0.41
PKP2	Plakophilin 2	0.41
ANGPT2	Angiopoietin 2	0.41
HSD3B1	Hydroxy-Delta-5-Steroid Dehydrogenase, 3 Beta- And Steroid Delta-Isomerase 1	0.41
LIF	LIF Interleukin 6 Family Cytokine	0.41
SRD5A3	Steroid 5 Alpha-Reductase 3	0.41
OSM	Oncostatin M	0.41
GNRH1	Gonadotropin Releasing Hormone 1	0.41
IL11	Interleukin 11	0.41
TGFB1I1	Transforming Growth Factor Beta 1 Induced Transcript 1	0.41
CTF1	Cardiotrophin 1	0.41
DSC1	Desmocollin 1	0.41
TMEM135	Transmembrane Protein 135	0.41

GSDMA	Gasdermin A	0.41
MPZL3	Myelin Protein Zero Like 3	0.41
HSD17B13	Hydroxysteroid 17-Beta Dehydrogenase 13	0.41
IFNL1	Interferon Lambda 1	0.41
MIR22	MicroRNA 22	0.41
PDGFRB	Platelet Derived Growth Factor Receptor Beta	0.33
AREG	Amphiregulin	0.33
CDK2	Cyclin Dependent Kinase 2	0.27
CBS	Cystathionine Beta-Synthase	0.27
ABCC1	ATP Binding Cassette Subfamily C Member 1	0.27
ATIC	5-Aminoimidazole-4-Carboxamide Ribonucleotide Formyltransferase/IMP Cyclohydrolase	0.27
DCK	Deoxycytidine Kinase	0.27
SHMT1	Serine Hydroxymethyltransferase 1	0.27
FPGS	Folylpolyglutamate Synthase	0.27
MAP2	Microtubule Associated Protein 2	0.27
DPP8	Dipeptidyl Peptidase 8	0.27
HSD3BP4	Hydroxy-Delta-5-Steroid Dehydrogenase, 3 Beta, Pseudogene 4	0.27
HGF	Hepatocyte Growth Factor	0.19
DPYD	Dihydropyrimidine Dehydrogenase	0.19
ESRRB	Estrogen Related Receptor Beta	0.19
ITGB1	Integrin Subunit Beta 1	0.19
DHFR	Dihydrofolate Reductase	0.19
TUBB3	Tubulin Beta 3 Class III	0.19
F10	Coagulation Factor X	0.19
PRLR	Prolactin Receptor	0.19
GLI2	GLI Family Zinc Finger 2	0.19
MC1R	Melanocortin 1 Receptor	0.19
ECHS1	Enoyl-CoA Hydratase, Short Chain 1	0.19
UBE2D3	Ubiquitin Conjugating Enzyme E2 D3	0.19
CASP4	Caspase 4	0.19
BAD	BCL2 Associated Agonist Of Cell Death	0.19
TRPV1	Transient Receptor Potential Cation Channel Subfamily V Member 1	0.19
CD151	CD151 Molecule (Raph Blood Group)	0.19
F13A1	Coagulation Factor XIII A Chain	0.19
ABCC8	ATP Binding Cassette Subfamily C Member 8	0.19
DBI	Diazepam Binding Inhibitor, Acyl-CoA Binding Protein	0.19
NPY	Neuropeptide Y	0.19
HTR3A	5-Hydroxytryptamine Receptor 3A	0.19
NGFR	Nerve Growth Factor Receptor	0.19
TJP2	Tight Junction Protein 2	0.19

PCSK1	Proprotein Convertase Subtilisin/Kexin Type 1	0.19
STAMBP	STAM Binding Protein	0.19
CCNA2	Cyclin A2	0.19
FDXR	Ferredoxin Reductase	0.19
TCN2	Transcobalamin 2	0.19
CGA	Glycoprotein Hormones, Alpha Polypeptide	0.19
CD33	CD33 Molecule	0.19
PGF	Placental Growth Factor	0.19
UGT1A9	UDP Glucuronosyltransferase Family 1 Member A9	0.19
CRHR2	Corticotropin Releasing Hormone Receptor 2	0.19
GGH	Gamma-Glutamyl Hydrolase	0.19
GSTA1	Glutathione S-Transferase Alpha 1	0.19
GSTM1	Glutathione S-Transferase Mu 1	0.19
SRF	Serum Response Factor	0.19
PCSK2	Proprotein Convertase Subtilisin/Kexin Type 2	0.19
DLX3	Distal-Less Homeobox 3	0.19
FDX1	Ferredoxin 1	0.19
CD58	CD58 Molecule	0.19
DPP7	Dipeptidyl Peptidase 7	0.19
CRHBP	Corticotropin Releasing Hormone Binding Protein	0.19
PCSK6	Proprotein Convertase Subtilisin/Kexin Type 6	0.19
SOD3	Superoxide Dismutase 3	0.19
NTS	Neurotensin	0.19
KCNK4	Potassium Two Pore Domain Channel Subfamily K Member 4	0.19
CD160	CD160 Molecule	0.19
GSDMD	Gasdermin D	0.19
SCG5	Secretogranin V	0.19
ASIP	Agouti Signaling Protein	0.19
CKAP4	Cytoskeleton Associated Protein 4	0.19
SOCS4	Suppressor Of Cytokine Signaling 4	0.19
CARD16	Caspase Recruitment Domain Family Member 16	0.19
OVOL2	Ovo Like Zinc Finger 2	0.19
OVOL1	Ovo Like Transcriptional Repressor 1	0.19
GPR137	G Protein-Coupled Receptor 137	0.19
GSTT1	Glutathione S-Transferase Theta 1	0.19
CEP43	Centrosomal Protein 43	0.19
LOC108228200	Xe1 Enhancer Downstream Of PAX1	0.19
LOC108228202	PEC7 Enhancer Downstream Of PAX1	0.19
